# A multicomponent intervention to improve maternal infection outcomes

**DOI:** 10.1056/NEJMoa2512698

**Published:** 2025-11-19

**Authors:** David Lissauer, Luis Gadama, Catriona Waitt, Sonia Whyte, Girvan Burnside, Aiswarya Anilkumar, Regina Makuluni, Peace Okwaro, Liu Yang, Peter Waitt, Owen Musopole, Rosemary Bilesi, Bertha Maseko, Joel Lwasa, Richard Mugahi, Charles Olaro, Mohammed Lamorde, Mirriam Makuta, Chimwemwe Kachiwaya, Tionge Mkandawire, Adrian Malunga, Nyadani Chitsulo, Prisca Abitimo, Tabitha Ayabo, Andrew Weeks, James Martin, Karla Hemming, Ioannis Gallos, Edward J M Monk, Jennifer Riches, Chikondi Chapuma, Judith Nanyondo, Fabiana Lorencatto, Mark Monahan, Benedetta Allegranzi, Catherine Dunlop, Lou Atkins, Anna Rosala-Hallas, Tracy Roberts, Carrol Gamble, Address Malata, Nicola Desmond, Edward Kommwa, Abi Merriel, Wiliam Parry Smith, Rebecca Smith, Ivy Ndumu, Eleanor Williams, Bob Faque, Gertrude Banda, Alinane L Nyondo-Mipando, Adelline Twimukye, Tim Chater, Aristotelis Diplas, Vanessa Brizuela, Joao Paulo Souza, Jamie Rylance, James Cheshire, Lydia Hawker, Arri Coomarasamy, Mercedes Bonet

**Affiliations:** Institute of Life Course and Medical Sciences, University of Liverpool, Liverpool Women’s Hospital, Crown St, Liverpool, UK, L8 7SS; Malawi Liverpool Wellcome Research Programme, Chipatala Avenue P.O. Box 30096 Chichiri, Blantyre 3, Malawi; Kamuzu University of Health Sciences, Private Bag 360, Chichiri, Blantyre 3, Malawi; Institute of Life Course and Medical Sciences, University of Liverpool, Liverpool Women’s Hospital, Crown St, Liverpool, UK, L8 7SS; Infectious Diseases Institute, Makerere University College of Health Sciences, Kampala, Uganda; Institute of Life Course and Medical Sciences, University of Liverpool, Liverpool Women’s Hospital, Crown St, Liverpool, UK, L8 7SS; Institute of Population Health, University of Liverpool AND Liverpool Clinical Trials Centre, University of Liverpool; Liverpool Clinical Trials Centre, University of Liverpool, Liverpool, L69 3BX; Malawi Liverpool Wellcome Research Programme, Chipatala Avenue P.O. Box 30096 Chichiri, Blantyre 3, Malawi; Infectious Diseases Institute, Makerere University College of Health Sciences, Kampala, Uganda; Great Ormond Street Institute of Child Health, University College London, UK, WC1N 1EH; Infectious Diseases Institute, Makerere University College of Health Sciences, Kampala, Uganda; Institute of Life Course and Medical Sciences, University of Liverpool, AND Wirral University Teaching Hospital NHS Foundation Trust; Malawi Ministry of Health, Box 30377, Lilongwe; Malawi Liverpool Wellcome Research Programme, Chipatala Avenue P.O. Box 30096 Chichiri, Blantyre 3, Malawi; Kawempe National Referral Hospital, Kampala, Uganda; Ministry of Health, P.O Box 7272, Kampala, Uganda; Malawi Liverpool Wellcome Research Programme, Chipatala Avenue P.O. Box 30096 Chichiri, Blantyre 3, Malawi; Infectious Diseases Institute, Makerere University college of health sciences, Kampala, Uganda; Institute of Life Course and Medical Sciences, University of Liverpool, Liverpool Women’s Hospital, Crown St, Liverpool, UK, L8 7SS; Department of Applied Health Sciences, University of Birmingham, Birmingham, B15 2TT, UK; UNDP/UNFPA/UNICEF/WHO/World Bank Special Programme of Research, Development and Research Training in Human Reproduction (HRP), Department of Sexual, Reproductive, Maternal, Child, Adolescent Health and Ageing, World Health Organization, Geneva, Switzerland; London School of Hygiene and Tropical Medicine, Keppel Street, London WC1E 7HT, United Kingdom; Institute of Life Course and Medical Sciences, University of Liverpool, Liverpool Women’s Hospital, Crown St, Liverpool, UK, L8 7SS; Malawi Liverpool Wellcome Program; Palladium, Kampala, Uganda; Centre for Behaviour Change, University College London, London, UK, WC1E 7HB; Department of Applied Health Sciences, University of Birmingham, Birmingham, B15 2TT, UK; World Health Organization, Eastern Mediterranean Regional Office, Cairo, Egypt; Department of Metabolism and Systems Sciences, University of Birmingham, Birmingham, B15 2TT, UK; Centre for Behaviour Change, University College London, London, UK, WC1E 7HB; Liverpool Clinical Trials Centre, University of Liverpool, Liverpool, L69 3BX; Department of Applied Health Sciences, University of Birmingham, Birmingham, B15 2TT, UK; Liverpool Clinical Trials Centre, University of Liverpool, Liverpool, L69 3BX; Malawi University of Science and Technology, P.O. Box 5196, Limbe; London School of Hygiene and Tropical Medicine, London WC1E 7HT, UK; Health-Sierra Leone; Institute of Life Course and Medical Sciences, University of Liverpool, Liverpool Women’s Hospital, Crown St, Liverpool, UK, L8 7SS; Keele University, School of Medicine, Keele, Staffordshire ST5 5BG and Shrewsbury; Telford Hospitals NHS Trust, Princess Royal Hospital, Apley Castle, Telford, Shropshire, Tf1 6TF; Institute of Life Course and Medical Sciences, University of Liverpool, Liverpool Women’s Hospital, Crown St, Liverpool, UK, L8 7SS; Department of Applied Health Sciences, University of Birmingham, Birmingham, B15 2TT, UK; Department of Applied Health Sciences, University of Birmingham, Birmingham, B15 2TT, UK; Malawi Liverpool Wellcome Research Programme, Chipatala Avenue P.O. Box 30096 Chichiri, Blantyre 3, Malawi; Malawi Liverpool Wellcome Research Programme, Chipatala Avenue P.O. Box 30096 Chichiri, Blantyre 3, Malawi; Institute of Life Course and Medical Sciences, University of Liverpool, Liverpool, UK, L7 8TX; Infectious Diseases Institute, College of Health Sciences, Makerere University, Kampala, Uganda; Liverpool Clinical Trials Centre, University of Liverpool, Liverpool, UK; UNDP/UNFPA/UNICEF/WHO/World Bank Special Programme of Research, Development and Research Training in Human Reproduction (HRP), Department of Sexual, Reproductive, Maternal, Child, Adolescent Health and Ageing, World Health Organization, Geneva, Switzerland; Department of Social Medicine, Ribeirao Preto Medical School, University of Sao Paulo, Ribeirao Preto, Brazil; World Health Emergencies Programme, WHO, Geneva, Switzerland; Department of Metabolism and Systems Sciences, University of Birmingham, Birmingham, B15 2TT, UK; Institute of Life Course and Medical Sciences, University of Liverpool, Liverpool Women’s Hospital, Crown St, Liverpool, UK, L8 7SS; Nuffield Department of Women’s and Reproductive Health, University of Oxford, Oxford; UNDP/UNFPA/UNICEF/WHO/World Bank Special Programme of Research, Development and Research Training in Human Reproduction (HRP), Department of Sexual, Reproductive, Maternal, Child, Adolescent Health and Ageing, Geneva, Switzerland

## Abstract

**Background:**

Maternal infection and sepsis remain major causes of maternal death and severe morbidity worldwide, particularly in low- and middle-income countries. Inconsistent implementation of evidence-based recommendations for infection prevention and management and delays in detection and treatment of maternal sepsis contribute to preventable deaths.

**Methods:**

We conducted a cluster-randomized trial to assess a multicomponent intervention, the Active Prevention and Treatment of Maternal Sepsis (APT-Sepsis) program. This program supported healthcare providers to achieve three goals; 1) compliance with the WHO 5 moments of hand hygiene, 2) adoption of evidence-based practices for maternal infection prevention and management and 3) early detection of sepsis and use of the FAST-M treatment bundle (**f**luids, **a**ntibiotics, **s**ource control, **t**ransfer if required and **m**onitoring). The control group had usual care, with passive guideline dissemination. The primary outcome was a composite of maternal infection-related mortality or severe morbidity (infection-related maternal near miss, deep surgical or perineal site infection, or body-cavity infection) among pregnant or recently pregnant women.

**Results:**

A total of 59 hospitals across Malawi and Uganda, with 431,394 women giving birth during the trial, were randomly assigned to intervention (n=30 clusters) or control groups (n=29). The incidence of maternal infection–related mortality or severe morbidity was 1.4% in the intervention group and 1.9% in the usual care group (risk ratio, 0.68; 95% confidence interval, 0.55 to 0.83; P<0.001). This effect was generally consistent across countries and facility size and sustained over time.

**Conclusions:**

Implementing the APT-Sepsis intervention reduced a composite of maternal infection-related mortality and severe morbidity, compared to usual care.

(Funded by the Joint Global Health Trials scheme and others; APT-Sepsis ISRCTN number, ISRCTN42347014.)

Maternal infection is an important cause of maternal morbidity and mortality worldwide, contributing to as many as half of in-hospital maternal deaths, with the greatest burden observed in low- and middle-income countries.^1–3^ Infections during and after pregnancy are also associated with long-term morbidity for women and with adverse perinatal outcomes, such as stillbirth and neonatal death.^4–8^ Outcomes are particularly poor for maternal sepsis.^9^

Global initiatives have identified the prevention and management of maternal infection and sepsis as a priority.^10–13^ Several upstream deficiencies in care are critical contributors to maternal deaths from sepsis, including inconsistent adherence to infection-prevention practices, inappropriate antibiotic use and delays in the recognition and treatment of infection and sepsis.^3,14–16^ These shortcomings are compounded by systemic constraints, including inadequate staffing, overcrowded facilities, and limited supplies of key resources.^17, 18^

The World Health Organization (WHO) has issued recommendations on adherence to hand-hygiene standards and evidence-based practices to prevent and treat maternal infections.^19–21^ Yet compliance with these recommendations is suboptimal.^3,14^ Structured tools and bundles of care have been shown to improve recognition, timely treatment and outcomes for other obstetric emergencies such as postpartum hemorrhage, even in resource constrained health facilities.^22^ Moreover, sepsis bundles are widely used in high-income countries, particularly in the non-maternity population.^23,24^ A maternal sepsis bundle has been developed specifically for low resource settings, but its effect on maternal outcomes has not been established.^25,26^

The **A**ctive **P**revention and **T**reatment of Maternal Sepsis (APT-Sepsis) program was designed to address these deficiencies through an integrated, facility-level multicomponent intervention. The program seeks to help health workers achieve three goals: improved hand-hygiene compliance, improved maternal infection prevention and management, and early recognition and bundled treatment of sepsis.^25,26^ We conducted a large cluster-randomized trial in Malawi and Uganda to evaluate whether implementation of the APT-Sepsis program in health facilities would reduce infection-related maternal mortality and severe morbidity.

## Methods

### Trial design and oversight

The APT-Sepsis trial was a multi-country, two-arm parallel cluster-randomized trial with a baseline control phase. The intervention was delivered at the health facility level (clusters), targeting the behaviors of healthcare providers and systems within the facilities.

There was a baseline phase of at least 6 months in all participating facilities, during which they provided usual care. At completion of the baseline phase, facilities were randomly assigned in a 1:1 ratio to receive the trial intervention or continue providing usual care for 12 months. A transition period of 3 months allowed for training of champions and embedding of the intervention into hospital systems; data from this period were not included in the effectiveness analysis. Malawi sites were randomized between November 6, 2023 and January 8, 2024, and Uganda sites between January 8, 2024 and March 4, 2024.

A minimization algorithm generated by an independent statistician was used to ensure balance between facilities allocated to the intervention and control groups within each country. Minimization factors were the number of live births per cluster per week (categorized in tertiles) and the proportion of births with the composite primary outcome (categorized by median split) during the baseline phase. Facilities were allocated sequentially. A random element was incorporated so each facility was given the allocation that would minimize the imbalance with 90% probability, or the other allocation with 10% probability. A mixed-methods process evaluation explored additional implementation outcomes of acceptability, feasibility, mechanisms of change and cost (as part of a formal economic evaluation).

The trial was approved by the University of Liverpool, the WHO Ethics Review Committee, the College of Medicine Research Ethics Committee in Malawi, the Infectious Diseases Institute Research Ethics Committee and Uganda National Council for Science and Technology in Uganda. Patients were not individually consented as the intervention was delivered to healthcare providers, and the components were considered best practice. Agreement for participation was obtained at a national and facility level.

Trial oversight and monitoring were provided by a trial steering committee and an independent data monitoring committee. Patient and Public Involvement groups provided advice on trial design and materials, how best to engage the public and on study-related messaging. The first and last author, and the trial statisticians from the Liverpool Clinical Trials Centre vouch for the accuracy and completeness of the data and for the fidelity of the trial to the protocol, available with the full text of this article at NEJM.org.

### Participating hospitals

Health facilities in Malawi and Uganda, with at least 1500 births per year, providing comprehensive obstetric care (able to perform cesarean births and provide blood transfusions) were eligible. Participation was subject to a trial-specific readiness assessment process, that ensured the availability of basic site prerequisites such as a water supply and electricity. Facilities were widely spread geographically in both countries.

### APT-Sepsis intervention and usual care

**Figure 1:**
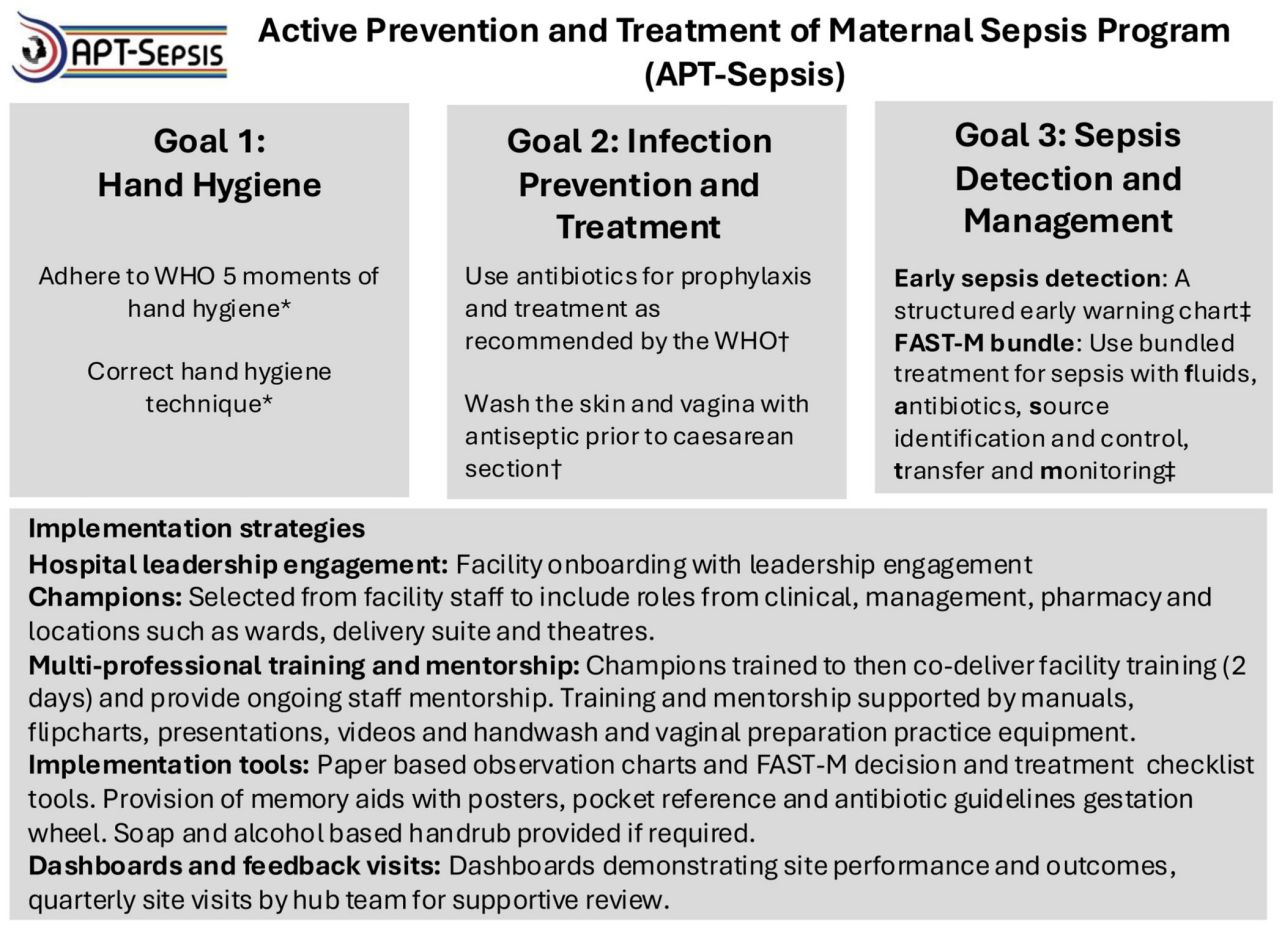
The Active Prevention and Treatment of Maternal Sepsis (APT-Sepsis) Intervention This multi-component intervention enabled healthcare workers to meet 3 goals, with implementation supported by a strategy designed to promote behavioural change. *Healthcare workers should handwash with soap or cleanse with alcohol based handrub their hands: 1) Before touching the woman or newborn, 2) Before a clean or aseptic procedure, 3) After body fluid exposure risk, 4) After touching a woman or newborn, 5) After touching a woman or newborn’s surroundings. Effective technique is required including appropriate glove use. † Use antibiotic prophylaxis in: preterm-prelabour rupture of membranes, manual removal of the placenta, abortion or miscarriage surgery, operative vaginal birth, 3^rd^ or 4th degree tears and prior to caesarean section. Antibiotic prophylaxis should not be used in: uncomplicated pregnancy or birth, pre-term labour with intact membranes, meconium stained amniotic fluid, episiotomy. Caesarean section: Use antiseptic solution for preparing skin and vagina. ‡ Detect sepsis early: Maternal vital signs measured at least daily. Recorded on colour coded early warning chart. Red flag abnormalities + infection triggers use of the FAST-M bundle. FAST-M bundle: To be completed within 1 hour. Fluids - 500ml crystalloid bolus, repeated if hypotension persists. Antibiotics - According to source or if unknown ceftriaxone 2g IV OD and metronidazole 500mg IV TDS (or 400mg PO TDS) with additional singe dose gentamicin 5mg/kg IV if haemodynamically unstable. Source: Identify and remove/treat the source Transfer: Transfer if required to a different hospital or location that can provide higher level of care. Monitoring: Repeat maternal observation every 30 minutes until stable, neonatal monitoring and review if required.

APT-Sepsis is a multi-component intervention delivered as an integrated program (Figure 1), aiming to help providers achieve three goals: 1) perform hand-hygiene according to the WHO 5 moments of hand hygiene approach, with the correct technique,^19^ 2) follow WHO recommendations on infection prevention and management during and after pregnancy, including the evidence-based use of antibiotics for prophylaxis and treatment of common maternal infections, and the correct preparation of the skin and vagina with antiseptic solution prior to cesarean surgery,^20^ and 3) detect sepsis early and initiate the FAST-M treatment bundle (**f**luids, **a**ntibiotics, **s**ource identification and control, assessment of the need for **t**ransfer to a higher level of care, and **m**onitoring of the woman and baby) when sepsis is suspected. A standardized observation chart that provided clear thresholds for triggering the bundle was used for all patients in the intervention facilities. Intervention components were derived using an iterative process of evidence synthesis, international expert consensus and optimization through multi-site pilot studies and mixed-methods evaluation.^25–27^

Implementation strategies were developed from a behavior change perspective^28^ and include the following key components: hospital leadership engagement; program champions selected from existing facility staff; multi-disciplinary training with comprehensive training materials; implementation tools (eg FAST-M checklist); and performance feedback with dashboards and quarterly visits (Figure 1; details in the Supplementary Appendix). Key resources such as antibiotics were obtained by both the intervention and control sites through their usual procurement pathways. Additional soap and alcohol hand rub were provided to intervention group facilities if needed. A small number of thermometers and blood pressure machines were also supplied on one occasion to any site if not adequate at the site readiness assessment.

The control facilities continued with usual care and were provided with the relevant WHO and national guidelines on hand hygiene and maternal infection prevention and treatment that informed the APT-Sepsis program (passive guideline dissemination). After the study ended, the APT-Sepsis intervention was rolled out to all the sites that had been allocated to usual care.

### Outcome measures

The primary outcome was infection-related maternal death or severe morbidity. This was defined as a composite of infection-related maternal mortality, infection-related maternal “near-miss” events, and severe infection (deep surgical-site, deep perineal or body-cavity infection) during pregnancy, childbirth or within 42 days of pregnancy ending, at any time up to 28 days of discharge (whichever occurred first). Outcome definitions are provided in the Supplementary appendix. WHO criteria for near-miss were modified to ensure ascertainment would not be influenced by the intervention. Modified CDC criteria were used to define deep surgical site infection, deep perineal/labial/vaginal tear infection, and reproductive tract or body cavity infection within 30 days after the procedure or birth. For maternal mortality and near-miss events, attribution to maternal infection was assessed based on review of the full clinical record by the site-based clinical data collector and by the central country clinical team. If there was discordance in assessments, or if uncertainty in the causation was recorded by either group, the case was adjudicated by a separate case classification committee blinded to site allocation.

Secondary outcomes included: individual components of the primary composite outcome; stillbirth; early neonatal death (infection-related and total); maternal mortality (any cause); maternal near-miss (any cause); and maternal severe acute respiratory infections. Clinical outcomes were recorded by study staff, independent of the implementation team. Clinical areas were monitored at each site and objective, structured reporting conducted daily in the baseline and post-randomization phases; identification of outcomes involved active case finding, chart review and site records assessment.

Additional secondary outcomes, related to Implementation, included: compliance with hand hygiene; correct antibiotic prophylaxis at cesarean section; complete vital sign recording; and compliance with maternal sepsis management bundle. These were measured quarterly during the intervention phase in both intervention and usual care facilities.

### Statistical analysis

We calculated that at least 60 clusters (a minimum of 30 in Malawi and 30 in Uganda) would be required for the trial to have 95% power, with 2 sided p < 0.05, to detect a 25% relative reduction in the composite primary outcome from 3% to 2.25%. This calculation adjusted for clustering, (assuming an intra-cluster correlation (ICC) of 0.03 (range: 0.001 to 0.05) and variation in clustering over time, assuming a cluster autocorrelation of 0.97 (range: 0.9 to 1.0). The original sample size calculations were based on an intervention period of 20 months. A pre-specified re-estimation of the sample size was conducted once the intra-cluster correlation, baseline event rate and number of participants per cluster was known from the baseline phase. This demonstrated an ICC of 0.02 and a larger than expected number of participants per cluster. Based on these findings, shortening the intervention was expected to have minimal effect on power, and the independent data monitoring committee and trial steering committee recommended a revised intervention period of 12 months. A full sample-size justification is provided in the trial protocol.

All analyses were performed according to the intention-to-treat principle. There were no interim analyses during the trial.

In the primary analysis, we used generalized linear mixed effects models incorporating a constrained baseline approach. For this, both baseline and post-randomization timepoints were included as outcomes, but the treatment effect was constrained to be zero in the baseline phase. We used binomial distribution and logit link, with robust standard errors, followed by marginal standardization to estimate risk ratios and risk differences. Cluster and cluster by period were included as random effects, with country, and the categorical minimization factor of facility size included as covariates. The second minimization factor (proportion of births with the composite primary outcome) was not included as it was already in the model as the outcome variable.

We analyzed the treatment effect on the primary outcome in prespecified subgroups, according to country, facility size, and months post-implementation. Subgroup analyses were carried out by including a treatment group by subgroup interaction parameter in the regression model and reporting adjusted treatment effects with 95% confidence intervals [CI].

Secondary outcomes were analyzed using the same methods as the primary outcome. Implementation outcomes from the quarterly visits in each facility were analyzed using mixed effect repeated measures linear regression with country and facility size included as covariates. There was no prespecified plan to adjust for multiplicity in tests of secondary outcomes. The widths of the 95% confidence intervals around point estimates have not been adjusted for multiplicity and should not be used to infer definitive treatment effects.

## Results

### Hospital and participant characteristics

A total of 83 health facilities were identified and underwent initial assessment for eligibility (Fig. S1). Of these, 33 facilities in Malawi and 38 facilities in Uganda proceeded to a readiness assessment and baseline data collection. Three facilities in Malawi and nine in Uganda were then excluded for the following reasons: they did not manage patients with severe infections or sepsis within the facility, they no longer met the eligibility criteria or, in Malawi, the maximum number of facilities were already included (Fig. S1).

A total of 59 facilities underwent randomization, with 30 assigned to the intervention group (15 in Malawi and 15 in Uganda) and 29 to usual care (15 and 14, respectively). Following randomization, all sites received the planned intervention, and completed the study. There were a total of 431,394 women with live births during the study (190,500 in the baseline phase and 240,894 in the intervention phase, Table 1.)

Facility characteristics and resource availability appeared generally similar between intervention and control groups at baseline; availability was low for some key resources. (Table 1). Table S1 summarizes the representativeness of the study population.

### Outcomes

**Figure 3:**
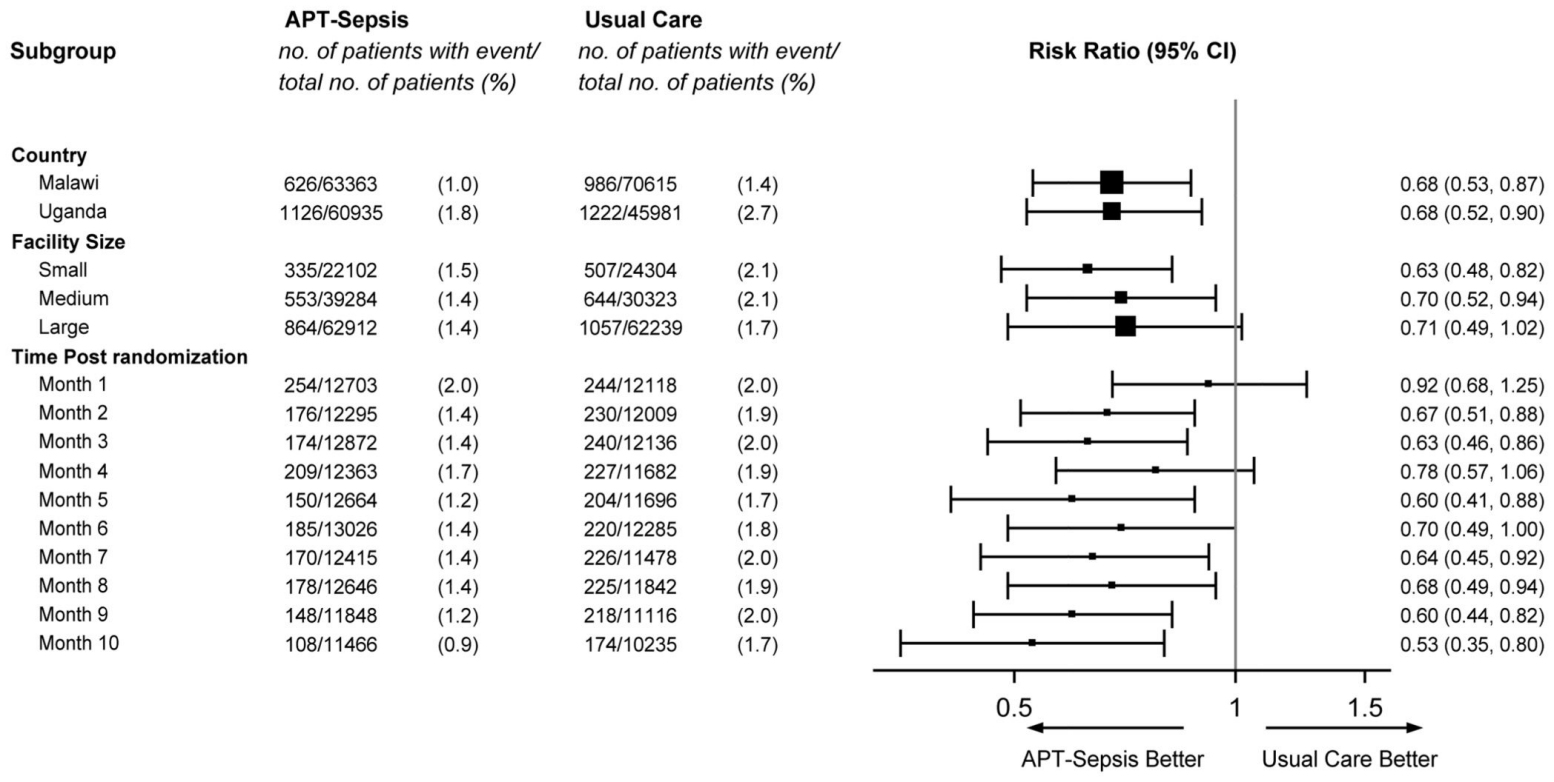
Primary Outcome According to Prespecified Subgroups. Shown are forest plots of the risk ratios for the primary outcome; a composite of maternal infection-related mortality or severe morbidity (infection-related maternal near miss, deep surgical or perineal site infection, or body-cavity infection) among pregnant or recently pregnant women, according to prespecified subgroups. These were country, facility size that was defined by tertile at the point of randomization, within each country and time point in months post randomization. The size of the square represents the relative numbers in each subgroup, with bars representing 95% confidence intervals. The width of the confidence intervals for the subgroup analysis have not been adjusted for multiplicity and cannot be used to infer treatment effects.

The primary outcome occurred in 1752 of 124,298 women (1.4%) in the intervention group and in 2208 of 116,596 (1.9%) in the usual care group (risk ratio, 0.68; 95% CI, 0.55 to 0.83; P<0.001) (Table 2 and S2). This effect appeared generally consistent between countries (Fig. 3 and Table S4) and across small, medium and large facilities. The incidence of the primary outcome in the intervention group progressively decreased following randomization, with a mean rate of 2.4% at baseline, 2% in the first month following implementation and completion of the transition phase, and 0.9% in the final month of the study. The effect size correspondingly increased from the first month (risk ratio, 0.92; 95% CI, 0.68 to 1.25) to the final month of the intervention phase (risk ratio, 0.53; 95% CI, 0.35 to 0.80) (Fig. 3, and Table S4).

Components of the primary outcome are shown in Table 2. The reduction in the primary composite outcome appeared largely driven by the effect of the intervention on severe infection-related morbidity (incidence 1.35% in the intervention group and 1.80% in the usual care group; risk ratio, 0.68; 95% CI, 0.55 to 0.84).

Stillbirth was recorded in 2.13% of total births in the intervention group and 1.95% in the usual care group (risk ratio, 0.90; 95% CI, 0.73 to 1.10) Neonatal deaths were recorded in 2.16% and 2.37%, respectively (risk ratio, 0.88; 95% CI, 0.73 to 1.04); these were attributable to infection in 0.66% and 0.53%, respectively (risk ratio, 0.86; 95% CI, 0.57 to 1.30)(Tables 2 and S2).

The intervention was associated with improvements in the prespecified implementation outcomes targeted to the three goals of the intervention (Table 2). Mean hand hygiene compliance (Goal 1) was 33% in the intervention sites compared to 15% in the usual care sites (mean difference 14%; 95% CI 10% to 19%).

Appropriate antibiotic prophylaxis was appropriately administered prior to cesarean section (Goal 2) in 74% and 58% of patients, respectively (mean difference 15%; 95% CI 4% to 26%). Several measures related to Goal 3 also supported primary outcomes findings; for example, complete observations at admission in 48% vs 15%, respectively (mean difference 32%; 95% CI 25% to 40%) and, among patients with suspected sepsis, antibiotics administered within 1 hour in 44% vs 38%, respectively (mean difference 8%; 95% CI -3% to 19%).

## Discussion

In this cluster randomized trial, implementation of the Active Prevention and Treatment of Maternal Sepsis (APT-Sepsis) program resulted in a significant reduction in infection-related maternal mortality or severe morbidity compared to usual care in pregnant or recently pregnant women. This benefit was driven by the reduction in deep surgical or perineal site, or body cavity infection with the intervention, was consistent across countries and facility size, and was sustained throughout the trial.

The scale of the trial, including government and non-government facilities of different sizes, supports the generalizability of the findings to health facilities providing comprehensive obstetric care. The intervention also covered the continuum of pregnancy-related infections, including in early pregnancy or following abortions. The intervention involved the provision of relatively few additional resources beyond what was generally available within the hospital systems, suggesting feasibility beyond the trial setting. For example, minimal equipment was provided, and site champions were not paid for this extra role.

Results for implementation outcomes suggested improvement at intervention sites across all three program goals. However, adherence was incomplete; this was not unexpected given that the program did not address broader health system challenges (eg staffing shortages, inadequate sinks for handwashing, limited antibiotic availability). The observed clinical benefit despite modest changes in some implementation measures may reflect the simultaneous targeting of multiple points in the pathway to maternal sepsis and adverse outcomes.

A recent trial demonstrated that azithromycin prophylaxis before all vaginal births reduced maternal sepsis,^29^ but this practice has not been routinely implemented owing to concerns regarding antimicrobial resistance.^30^ Our intervention reduced maternal infection with targeted use of antibiotics together with non-pharmacological changes in care.

The trial has several limitations. The multicomponent nature of the intervention precludes attribution of the effect to individual elements. Microbiological data were not available, preventing pathogen-specific diagnosis or resistance profiling. As study staff identifying outcomes were not blinded to group allocation, and there is subjectivity in identifying the relatedness of outcomes to infection, bias is possible. However, clinical outcomes were identified using objective criteria and collected daily by trained staff not involved in implementation. Identification of outcomes after hospital discharge required that the patient return for care, so underreporting is possible, but less likely given the serious nature of these outcomes. Extending this intervention to other countries and setting may require partnering with national ministries of health, as we did, to facilitate uptake of the intervention, and adapting materials and processes to ensure they are culturally and contextually appropriate. Further work is needed to evaluate patient and provider experience, behavior changes and cost-effectiveness of the intervention.

In summary, implementation of the APT-sepsis program substantially reduced the risk of the primary outcome of infection-related mortality and severe morbidity.

Disclosure forms provided by the authors are available with the full text of this article at NEJM.org.

## Supplementary Material

supplement

## Figures and Tables

**Figure 2:**
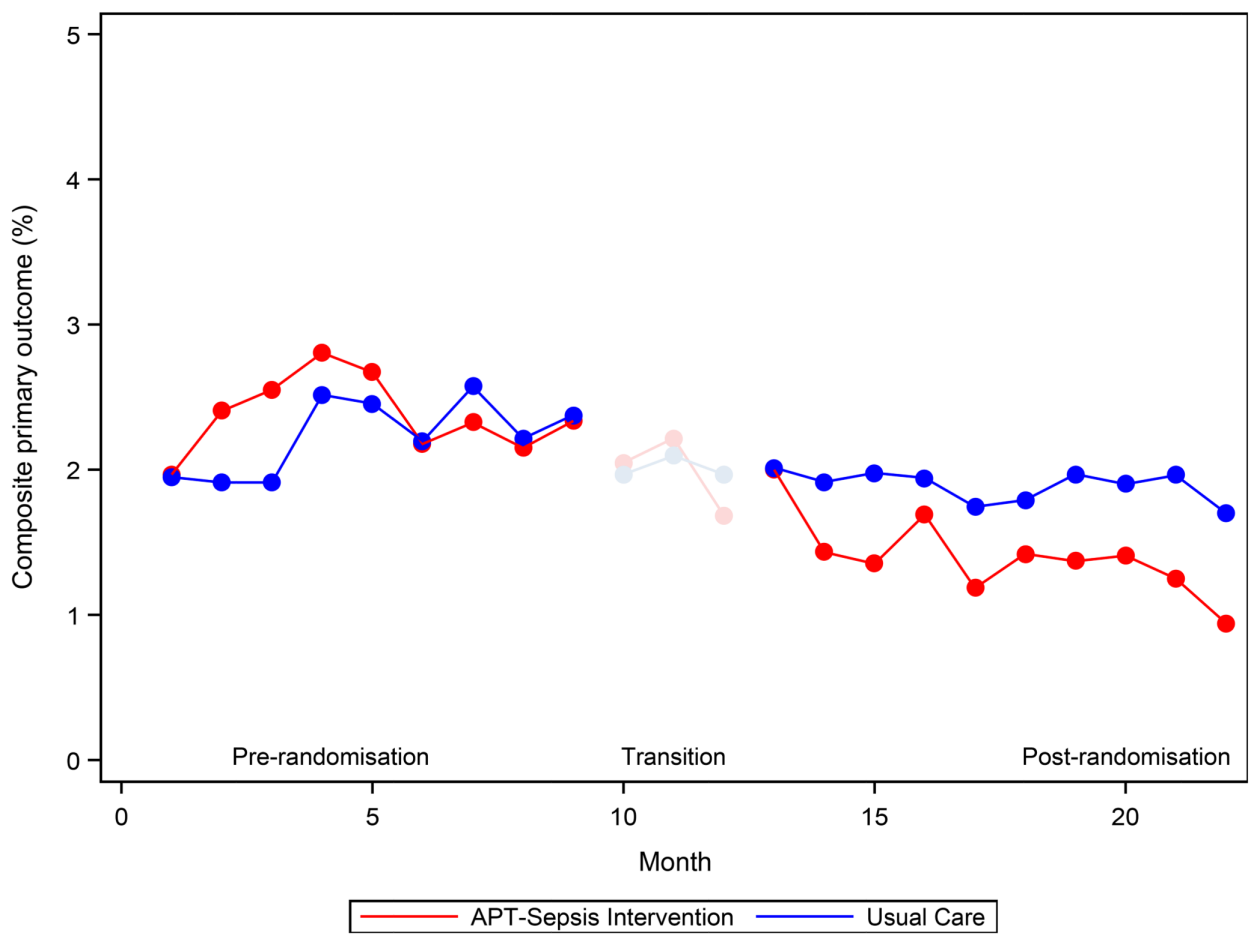
Patients with the Primary Outcome during the Baseline, Transition and Implementation periods. The primary outcome was a composite of maternal infection-related mortality or severe morbidity (infection-related maternal near miss, deep surgical or perineal site infection, or body-cavity infection) among pregnant or recently pregnant women. The transition phase, a 3 month period involving training of champions and embedding of the intervention into hospital systems, was not included in the analysis.

**Table 1:** Characteristics of participating facilities and resource availability at baseline*

Characteristic	Intervention (30 clusters)	Usual Care (29 clusters)
Live births – no.	94,730	95,770
Early pregnancy loss – no.	9,656	9,358
Stillbirths – no.	2,267	2,054
Neonatal deaths – no. (%)	2,334 (2.5)	2,456 (2.6)
Vaginal births – no. (%)^†^	67,343 (71.1)	70,512 (73.6)
Forceps or vacuum births – no. (%)^†^	1,346 (1.4)	1,564 (1.6)
Cesarean section births – no. (%)^†^	24,154 (25.5)	21,205 (22.1)
Vaginal breech delivery births – no. (%)^†^	1,346 (1.4)	1,564 (1.6)
Born before arrival – no. (%)^†^	1259 (1.4)	1575 (1.6)
Postpartum hemorrhage (>1 liter) – no. (%)^†^	1,680 (1.8)	1,355 (1.4)
Severe pre-eclampsia or eclampsia – no. (%)^†^	1,201 (1.3)	1,262 (1.3)
Median availability of key resources - % of weeks where available (LQ-UQ)		
Functioning autoclave^‡^	100 (97.1-100)	100 (96.0-100)
Running water^‡^	83.7 (8.8-97.1)	85.4 (35.3-96.9)
Thermometers^‡^	44.4 (4.0-80.5)	19.5 (5.6-71.9)
Blood pressure devices^‡^	6.8 (0-16.7)	2.6 (0-19.5)
Soap^‡^	80.9 (43.8-96.9)	71.9 (38.2-94.1)
Alcohol based hand rub^‡^	66.1 (31.7-88.9)	68.8 (36.1-92.7)
Oxygen concentrators^‡^	54.6 (7.3-83.3)	44.4 (2.6-84.4)
Bottle or piped oxygen^‡^	9.9 (0-46.9)	0 (0-25.0)
IV fluids (crystalloid) ^‡^	84.7 (61.0-95.1)	81.6 (50.0-96.9)
IV Cephalosporin^‡^	66.6 (33.3-92.7)	75.6 (58.8-87.5)
IV Metronidazole^‡^	60.6 (36.6-82.4)	63.2 (44.1-81.3)
IV Gentamycin^‡^	72.1 (50.0-82.9)	65.9 (55.6-93.8)

* Hospitals in the intervention phase implemented the APT-Sepsis intervention. This was a cluster randomised trial with baseline phase. The characteristics reported here are from the baseline phase, prior to randomization. LQ denotes lower quartile. UQ denotes upper quartile. IV denotes intravenous.

† Percentages were calculated from the number of live births.

‡ Availability of key resources were recorded weekly from key clinical areas as either available, limited or not available, in each facility. Here the median percentage of weeks for which the resource was available in all areas assessed is reported.

**Table 2:** Primary and secondary outcomes.*

Outcome	Intervention (N=124,298)	Usual Care (N=116,596)	Risk Ratio or Mean Difference (95% CI)
**Primary outcome**			*Risk Ratio* *(95% CI)*
Composite of infection-related maternal mortality, or infection-related near-miss, or deep surgical/perineal site infection or body cavity infection. (%)^†^	1752/124,298 (1.4)	2208/116,596 (1.9)	0.68 (0.55 to 0.83) ^‡^
**Components of the primary outcome** ^§^			
Infection-related maternal mortality	90/124,298 (0.07)	77/116,596 (0.07)	0.96 (0.69 to 1.32)
Infection-related near-miss	119/124,298 (0.10)	141/116,596 (0.12)	0.82 (0.54 to 1.25)
Deep surgical/perineal site infection or body cavity infection	1672/124,298 (1.35)	2102/116,596 (1.80)	0.68 (0.55 to 0.84)
**Secondary outcomes**			
Stillbirth^¶^	2708/127,006 (2.13)	2314/118,910 (1.95)	0.90 (0.73 to 1.10)
Neonatal death^||^	2691/124,298 (2.16)	2761/116,596 (2.37)	0.88 (0.73 to 1.04)
Neonatal death (infection-related)	819/124,298 (0.66)	622/116,596 (0.53)	0.86 (0.57 to 1.30)
Maternal mortality (any cause)	288/124,298 (0.23)	235/116,596 (0.20)	0.96 (0.74 to 1.24)
Maternal near miss (any cause)	771/124,298 (0.62)	609/116,596 (0.52)	0.90 (0.73 to 1.10)
Maternal severe acute respiratory infection^~^	10/124,298 (0.01)	7/116596 (0.01)	1.04 (0.45 to 2.39)
**Implementation outcomes** ^#^			*Mean Difference* *(95% CI)*
Hand hygiene compliance – mean % (SD)	32.9 (19.3)	15.1(10.5)	14.48 (10.1 to 18.9)
Correct cesarean section antibiotic prophylaxis – mean % (SD)	73.7 (32.5)	57.7 (36.4)	15.0 (4.0 to 26.0)
Complete vital signs recorded at admission – mean % (SD)	48 (29)	14.5 (19.5)	32.4 (24.5 to 40.4)
Patients with suspected sepsis with complete vital signs recorded – mean % (SD)	59.9 (32.6)	33.9 (39.0)	27.7 (15.2 to 40.2)
Patients with suspected sepsis given IV fluids within 1 hour – mean % (SD)	32.9 (33.6)	21.9 (24.5)	13.4 (4.8 to 22.0)
Patients with suspected sepsis given antibiotics within 1 hour – mean % (SD)	43.6 (37.5)	38.4 (37.7)	8.2 (-2.7 to 19.0)

* The width of the confidence intervals for secondary outcomes have not been adjusted for multiplicity and should not be used to infer treatment effects.

† The primary outcome was infection-related maternal death or severe morbidity. This was defined as a composite of infection-related maternal mortality, infection-related maternal near-miss (adapted WHO criteria) and severe infection-related morbidity (adapted CDC definition of deep surgical site infection or body cavity infection) during pregnancy, childbirth or within 42 days of pregnancy ending, at any time up to 28 days of discharge (whichever occurred first). The denominator is live births. The intracluster correlation for the primary outcome was 0.004 (95% CI, 0.003 to 0.005). The cluster auto-correlation for the primary outcome was 0.398. These were estimated by fitting a mixed-effects linear effects model to the data with random effects for cluster and for a cluster-period interaction.

‡ P<0.001

§ An individual could be included in more than one component of the primary outcome, but would only have been counted once when the primary outcome was calculated

¶ A baby that died before of during birth after a gestational age of 28 weeks, with gestational age as determined by the facility medical team. The denominator includes livebirths and stillbirths.

|| Death of a live-born infant within the first 28 completed days of life, only deaths occurring within the health facility were reported.

~ Defined as deaths or near miss events due to maternal severe acute respiratory infection

# Implementation outcomes were measured during quarterly site assessment visits, and are reported as a mean proportion of opportunities or cases in which there was compliance. The unit of analysis was the facility-quarter rather than the participant. Mean differences and 95% confidence intervals are compared between intervention and control.
