# Supplementary material for: A multicomponent intervention to improve maternal infection outcomes
Source: N Engl J Med. Author manuscript; Available in PMC 2026 Apr 30. (PMC7618407; doi:10.1056/NEJMoa2512698)
Supplement: supplement [file EMS210352-supplement-supplement.pdf]

Randomized trial of a multicomponent intervention to improve maternal infection outcomes

Supplementary Appendix

Table of Contents

List of Investigators 2
Additional Acknowledgements 6
Details of the APT-Sepsis Intervention and Implementation Strategy 13
Study Outcome Definitions 25
Figure S1: Randomization of Health Facilities in the Cluster-Randomized Trial 28
Table S1: Table on the representativeness of study participants 29
Table S2: Primary and secondary outcomes including pre-randomization period and absolute risk differences. 30
Table S3: Post-hoc sensitivity analyses of primary outcome 31
Table S4: Prespecified Subgroup Analysis of the primary outcome, including pre-randomization phase 32
Table S5: Primary cause of severe infection 33
Table S6: Characteristics of patients pre- and post-randomization 34
Table S7: Characteristics of women who died, had a near miss, or had a severe infection 35
Table S8 Estimates of intracluster correlation and cluster autocorrelation 37
Table S9: Health facility operating theatre and sterilization characteristics 38
Table S10: Health facility diagnostic equipment availability 39
Table S11: Health facility human resource availability 44
Table S12: Equipment availability over all areas in health facility 45
Table S13: Water and handwashing availability over all areas in facility 52
Table S14: Pharmacy supply availability – oral antibiotics 55
Table S15: Pharmacy supply availability – Intravenous antibiotics 61
Table S16: Availability of other pharmacy supplies 69
Table S17: Labor ward equipment availability 70
References 77

List of Investigators

David Lissauer, Ph.D., Institute of Life Course and Medical Sciences, University of Liverpool, Liverpool Women's Hospital, Crown St, Liverpool, UK, L8 7SS; Malawi Liverpool Wellcome Research Programme, Chipatala Avenue P.O. Box 30096 Chichiri, Blantyre 3, Malawi;

Luis Gadama, M.Med., Kamuzu University of Health Sciences, Private Bag 360, Chichiri, Blantyre 3, Malawi;

Catriona Waitt, Ph.D., Institute of Life Course and Medical Sciences, University of Liverpool, Liverpool Women's Hospital, Crown St, Liverpool, UK, L8 7SS; Infectious Diseases Institute, Makerere University College of Health Sciences, Kampala, Uganda;

Sonia Whyte, M.Sc., Institute of Life Course and Medical Sciences, University of Liverpool, Liverpool Women's Hospital, Crown St, Liverpool, UK, L8 7SS;

Girvan Burnside, Ph.D., Institute of Population Health, University of Liverpool AND Liverpool Clinical Trials Centre, University of Liverpool;

Aiswarya Anilkumar, M.Sc., Liverpool Clinical Trials Centre, University of Liverpool, Liverpool, L69 3BX;

Regina Makuluni, M.Sc., Malawi Liverpool Wellcome Research Programme, Chipatala Avenue P.O. Box 30096 Chichiri, Blantyre 3, Malawi;

Peace Okwaro, B.Sc., Infectious Disease Institute, Makerere University College of Health Sciences, Kampala, Uganda ;

Liu Yang, Ph.D., Great Ormond Street Institute of Child Health, University College London, UK, WC1N 1EH;

Peter Waitt, MB.Ch.B., Infectious Disease Institute, Makerere University College of Health Sciences, Kampala, Uganda; Institute of Life Course and Medical Sciences, University of Liverpool, AND Wirral University Teaching Hospital NHS Foundation Trust;

Owen Musopole, M.Sc., Malawi Ministry of Health, Box 30377, Lilongwe;

Rosemary Bilesi, M.Sc., Malawi Ministry of Health, Box 30377, Lilongwe;

Bertha Maseko, M.Sc., Malawi Liverpool Wellcome Research Programme, Chipatala Avenue P.O. Box 30096 Chichiri, Blantyre 3, Malawi;

Joel Lwasa, M.Med., Kawempe National Referral Hospital, Kampala, Uganda ;

Richard Mugahi, M.Sc., Ministry of Health, P.O Box 7272, Kampala, Uganda;

Charles Olaro, M.Med., Ministry of Health, P.O Box 7272, Kampala, Uganda;

Mohammed Lamorde, Ph.D., Resolve to Save Lives;

Mirriam Makuta, B.Sc., Malawi Liverpool Wellcome Research Programme, Chipatala Avenue P.O. Box 30096 Chichiri, Blantyre 3, Malawi;

Chimwemwe Kachiwaya, B.Sc., Malawi Liverpool Wellcome Research Programme, Chipatala Avenue P.O. Box 30096 Chichiri, Blantyre 3, Malawi;

Tionge Mkandawire, B.Sc., Malawi Liverpool Wellcome Research Programme, Chipatala Avenue P.O. Box 30096 Chichiri, Blantyre 3, Malawi;

Adrian Malunga, B.Sc., Malawi Liverpool Wellcome Research Programme, Chipatala Avenue P.O. Box 30096 Chichiri, Blantyre 3, Malawi;

Nyadani Chitsulo, B.Sc., Malawi Liverpool Wellcome Research Programme, Chipatala Avenue P.O. Box 30096 Chichiri, Blantyre 3, Malawi;

Prisca Abitimo, Dip. Midwifery, Infectious Diseases Institute, Makerere University college of health sciences, Kampala Uganda;

Tabitha Ayabo, B.Sc., Infectious Diseases Institute, Makerere University college of health sciences, Kampala Uganda;

Andrew Weeks, M.D., Institute of Life Course and Medical Sciences, University of Liverpool, Liverpool Women's Hospital, Crown St, Liverpool, UK, L8 7SS;

James Martin, Ph.D., Department of Applied Health Sciences, University of Birmingham, Birmingham, B15 2TT, UK;

Karla Hemming, Ph.D., Department of Applied Health Sciences, University of Birmingham, Birmingham, B15 2TT, UK;

Ioannis Gallos, M.D., UNDP/UNFPA/UNICEF/WHO/World Bank Special Programme of Research, Development and Research Training in Human Reproduction (HRP), Department of Sexual and Reproductive Health and Research, World Health Organization, Geneva, Switzerland;"

Edward J M Monk, M.Sc., London School of Hygiene and Tropical Medicine, Keppel Street, London WC1E 7HT, United Kingdom;

Jennifer Riches, MB.Ch.B., Institute of Life Course and Medical Sciences, University of Liverpool, Liverpool Women's Hospital, Crown St, Liverpool, UK, L8 7SS;

Chikondi Chapuma, M.P.H. Malawi Liverpool Wellcome Program;

Judith Nanyondo, M.P.H., Palladium, Kampala, Uganda;

Fabiana Lorencatto, Ph.D., Centre for Behaviour Change, University College London, London, UK, WC1E 7HB;

Mark Monahan, Ph.D., Department of Applied Health Sciences, University of Birmingham, Birmingham, B15 2TT, UK;

Benedetta Allegranzi, M.D., World Health Emergencies Programme, WHO, Geneva, Switzerland;

Catherine Dunlop, Ph.D., Department of Metabolism and Systems Sciences, University of Birmingham, Birmingham, B152TT, UK;

Lou Atkins, Ph.D., Centre for Behaviour Change, University College London, London, UK, WC1E 7HB;

Anna Rosala-Hallas, M.Sc., Liverpool Clinical Trials Centre, University of Liverpool, Liverpool, L69 3BX;

Tracy Roberts, Ph.D., Department of Applied Health Sciences, University of Birmingham, Birmingham, B15 2TT, UK;

Carrol Gamble, Ph.D., Liverpool Clinical Trials Centre, University of Liverpool, Liverpool, L69 3BX;

Address Malata, Ph.D., Malawi University of Science and Technology, P.O. Box 5196, Limbe;

Nicola Desmond, Ph.D., London School of Hygiene and Tropical Medicine, London, WC1E 7HT, UK;

Edward Kommwa, M.Med., Partners in Health-Sierra Leone;

Abi Merriel, Ph.D., Institute of Life Course and Medical Sciences, University of Liverpool, Liverpool Women's Hospital, Crown St, Liverpool, UK, L8 7SS;

Wiliam Parry Smith, M.D., Keele University, School of Medicine, Keele, Staffordshire ST5 5BG and Shrewsbury; Telford Hospitals NHS Trust, Princess Royal Hospital, Apley Castle, Telford, Shropshire, Tf1 6TF;

Rebecca Smith, MB.Ch.B., Institute of Life Course and Medical Sciences, University of Liverpool, Liverpool Women's Hospital, Crown St, Liverpool, UK, L8 7SS;

Ivy Ndumu, M.Sc., Department of Applied Health Sciences, University of Birmingham, Birmingham, B15 2TT, UK;

Eleanor Williams, M.Sc., Department of Applied Health Sciences, University of Birmingham, Birmingham, B15 2TT, UK;

Bob Faque, Malawi Liverpool Wellcome Research Programme, Chipatala Avenue P.O. Box 30096 Chichiri, Blantyre 3, Malawi;

Gertrude Banda, Malawi Liverpool Wellcome Research Programme, Chipatala Avenue P.O. Box 30096 Chichiri, Blantyre 3, Malawi;

Alinane L Nyondo-Mipando, Ph.D., Institute of Life Course and Medical Sciences, University of Liverpool, Liverpool, UK, L7 8TX;

Adelline Twimukye, M.A., Infectious Diseases Institute, College of Health Sciences, Makerere University, Kampala, Uganda;

Tim Chater, B.Sc., Liverpool Clinical Trials Centre, University of Liverpool, Liverpool, UK;

Aristotelis Diplas, M.Sc., Liverpool Clinical Trials Centre, University of Liverpool, Liverpool, UK;

Vanessa Brizuela, Dr.PH., UNDP/UNFPA/UNICEF/WHO/World Bank Special Programme of Research, Development and Research Training in Human Reproduction (HRP), Department of Sexual and Reproductive Health and Research, World Health Organization, Geneva, Switzerland;

Joao Paulo Souza, M.D., Ph.D., Department of Social Medicine, Ribeirao Preto Medical School, University of Sao Paulo, Ribeirao Preto, Brazil;

Jamie Rylance, Ph.D., World Health Emergencies Programme, WHO, Geneva, Switzerland;

James Cheshire, Ph.D., Department of Metabolism and Systems Sciences, University of Birmingham, Birmingham, B152TT, UK;

Lydia Hawker, MB.Ch.B., Institute of Life Course and Medical Sciences, University of Liverpool, Liverpool Women's Hospital, Crown St, Liverpool, UK, L8 7SS;

Arri Coomarasamy, Ph.D., Nuffield Department of Women's and Reproductive Health, University of Oxford, Oxford;

Mercedes Bonet, Ph.D., UNDP/UNFPA/UNICEF/WHO/World Bank Special Programme of Research, Development and Research Training in Human Reproduction (HRP), Department of Sexual and Reproductive Health and Research, World Health Organization, Geneva, Switzerland.

Additional Acknowledgements

We would like to acknowledge the contributions of the following people to the APT-Sepsis trial:

Trial Steering Committee (TSC) Members: Professor Rebecca Reynolds (CHAIR) University Edinburgh, Dr. Ann Phoya ONSE, Dr. Lisa Noguchi Jhpiego, Johns Hopkins University, Baltimore, Ms Edna Umali Public representative Dr Bejoy Nambiar UNICEF, Dr. Fanny Kachale Ministry of Health Malawi, Professor Kamija Phir, Professor Elizabeth Turner Duke University.

Independent Data Monitoring Committee (IDMC) Members: Professor Marian Knight (CHAIR)NPEU Oxford, UK, Professor Ed Juszczak University of Nottingham, UK, Professor Dharmintra Pasupathy University of Sydney, Australia, Dr. Chisale Mhango President Association Obstetrics and Gynaecology Malawi.

Classification Panel Members: Dr. Abi Merriel University of Liverpool , Professor William Parry-Smith The Shrewsbury and Telford NHS Trust, Dr. Edward Kommwa Koidu Government Hospital, Dr. Rebecca Smith University of Liverpool

PPIE Acknowledgement: We would like to sincerely thank all the Patient and Public Involvement and Engagement (PPIE) contributors who supported this study. Their insights, experience and feedback were invaluable in shaping the study, refining research materials, and ensuring the relevance and accessibility of our findings. Their contributions have greatly enriched the quality and impact of this work.

Supporting Organisations: We would like to acknowledge and thank the following organisations and individuals for their support with the APT-Sepsis study: **University of Liverpool** Sponsors office, Claire Hutchinson and Karen Wilding; Liverpool Clinical Trials Centre (LCTC) Quality Assurance Team; Research Support Office, **UNDP/UNFPA/UNICEF/WHO/World Bank Special Programme of Research, Development and Research Training in Human Reproduction (HRP), Department of Sexual and Reproductive Health and Research, World Health Organization, Geneva, Switzerland;** Dr Fernando Althabe; **World Health Emergencies Programme, WHO, Geneva, Switzerland;** Dr Miranda Deeves; **Infectious Diseases Institute (IDI) Uganda**; **Malawi-Liverpool-Wellcome Clinical Research Programme (MLW**); **Kireli creative design** studio and team; **Ministry of Health Malawi and facility leaders:** Dr Owen Musopole Deputy director, MoH; Rosemary Bilesi MPDSR national coordinator; Chikondi Nyson Principal Nursing and Midwifery Officer Facilitator, Supervisor; Clophat Baleti Clinical associate Facilitator, Supervisor; Emma Tsegula Nurse Midwife Facilitator, Supervisor; Juma Singano Clinical Officer Facilitator, Supervisor; Doreen Mwazani Nursing and Midwifery Officer Facilitator, Supervisor; George Lucius Clinical Officer Facilitator, Supervisor; John Munyimbiri Principal Nursing and Midwifery Officer Facilitator, Supervisor; Patrick Matchaya Anaesthetic clinician Facilitator, Supervisor; Eric Thuthuwa Anaesthetic clinician Facilitator, Supervisor; James Mbewe Medical Doctor Facilitator, Supervisor; Emmie Jingini Nursing and Midwifery Officer Facilitator, Supervisor; Brian Nyasulu Dental technician Facilitator, Supervisor; **Ministry of Health Uganda and Facility leaders:** Dr. Richard Mugahi Co-investigator; Dr. Charles Olaro Co-investigator; Dr. Joel

Njagala Calls Lwasa Ass. Consultant Obgyn; Dr. Jackie Akello Consultant Obgyn; Ms. Pasca Akurut Registered Midwife; Ms. Winnie Bamukisa Registered Midwife; Mr. Ronald Kaima Registered Nurse; Ms. Hadijah Nakatudde Technical Officer - Midwifery; Ms. Ritah Niwamanya Registered Midwife; Ms. Anitah Kusaasira Registered Midwife;

HUB Teams:

Malawi: Associate Prof Luis Gadama Principal Investigator; Regina Makuluni Trial Coordinator; Nyadani Chitsulo Data Manager; Chimwemwe Kachiwaya Research Nurse Coordinator; James Kasenda Research Clinician Coordinator; Mirriam Makuta Research Nurse Coordinator; Adrian Malunga Research Nurse Coordinator; Tionge Mkandawire Research Nurse Coordinator;

Uganda: Professor Catriona Waitt Principal Investigator; Dr Mohammed Lamorde Principal Investigator; Ms Peace Okwaro Trial Coordinator; Dr Peter Waitt Capacity Building Specialist; Ms Judith Nanyondo PhD scholar; Ms Tabitha Ayabo Project Officer; Ms Prisca Abitimo Research Midwife; Ms Diana Letasi Research Midwife; Ms Susan Akao Research Midwife; Mr Emmanuel Nkurunziza Data Officer; Ms Janet Nabisere Finance and Administration; Ms Adelline Twimukye Qualitative Analyst;

UK: Professor David Lissauer Chief investigator, Professor Carroll Gamble Director Liverpool Clinical Trials Centre, Dr. Girvan Burnside Lead Statistician, Mrs Sonia Whyte Senior Trial Manager, Dr Liu Yang Trial Manager, Miss Aishwarya Anilkumar Statistician; Mr Tim Chater Information systems Manager; Mrs Michelle Girvan Senior Data Manager; Mr Aristotelis Diplas Data Manager; Mrs Anna Rosala- Hallas Statistician;

Project Officers - Responsible for trial data collection

Malawi, Project Officers: Annie Banda Nurse Midwife; Dalitso Banda Clinical Associate; Nyasha Banda Nurse Midwife; Demobly Chagomerana Clinical Technician; Collins Chanza Clinical Technician; Mathews Chanza Clinical Technician; Chrispine Chapusa Clinical Technician; James Chauluka Nurse Midwife; Clement Chibwana Clinical Technician; Agness Chibwe Nurse Midwife; Patrick Chidanti Clinical Technician; Fatsani Chidule Clinical Technician; Mirriam Chikafalimani Nurse Midwife; Moses Chilongo Clinical Officer; Jessie Chima Nurse Midwife; Spain Chimaliro Nurse Midwife; Frank Chimbanga Nurse Midwife; George Chimenya Nurse Midwife; Blessings Chimgunya Nurse Midwife; Joseph Chimombo Clinical Associate; Melanie Chimtali Nurse Midwife; Levi Chirambo Clinical Associate; Paul Chirambo Clinical Officer; Fatsani Chisale Nurse Midwife; Solomon Chisale Nurse Midwife; Tracy Dinga Nurse Midwife; Aaron Dzombe Clinical Associate; Shadreck Gadama Clinical Technician; Phiphter Gangu Clinical Technician; Stanley Gombe Clinical Technician; Eves Hara Nurse Midwife; Peter Hara Clinical Technician; Francis House Clinical Technician; Thokozani Jizale Clinical Technician; Snoria Joseph Nurse Midwife; Jack Kachoka Nurse Midwife; Geaorge Kafumbi Clinical Technician; Euvancio Kambalame Nurse Midwife; Mervis Kambale Nurse Midwife; Dickson Kanowa Clinical Technician; Elita Kavala Nurse Midwife; Mtendere Kawaza Nurse Midwife; Charity Khobwe Nurse Midwife; Mirriam Khoka Nurse Midwife; Arthur Kondowe Clinical Technician; Sam Kuliana Clinical Technician; George Lawrence Clinical Technician; Tiyakhulenji ligomeka Nurse Midwife; Joane Lumbe Nurse Midwife; Catherine Mafanizo Nurse Midwife; Lloyd Mathewe Clinical Associate; Madalitso Mbewe Clinical Technician; Charles Mhone Clinical Technician; Ruth Mjuweni Clinical Technician; Tabwelako Mndungu Clinical

Technician; Witness Moveya Nurse Midwife; Maurice Mponda Clinical Associate; Esther Msonthi Nurse Midwife; Borniface Mtambalika Nurse Midwife; Maureen Mtambo Nurse Midwife; Lusungu Mtika Clinical Technician; Mirriam Mumba Nurse Midwife; Linda Mwafulirwa Nurse Midwife; Bongani Mwamlima Clinical Technician; Vincent Mwangonde Clinical Technician; Mike Mwape Clinical Technician; Peter Nankhonya Clinical Technician; Geoffrey Ndovie Nurse Midwife; Jonathan Ngwata Nurse Midwife; Ethel Njirazafa Nurse Midwife; Owen Nkungula Clinical Technician; Feliya Nyirenda Nurse Midwife; Frank Nyirenda Clinical Technician; Duncan Nyirongo Nurse Midwife; Timothy Phiri Clinical Technician; Chitsanzo Priscilla Kwawilira Medical Doctor; Majorobera Ramarikhoane Clinical Associate; Geoffrey Saizi Clinical Technician; Phoebe Sangano Nurse Midwife; Tiyanjane Singin Nurse Midwife; Janet Wister Nurse Midwife; Ritta Zamawa Nurse Midwife;

Uganda, Project Officers: Ms. Laura Avako; Ms. Margrate Kyazike; Ms. Mastura Nagginda; Ms. Jacinta Karuhanga; Ms. Redempta Tumwine; Dr. Augustine Ssemujju; Ms. Zulfa Ngonzi; Ms. Juliet Namwebya; Ms. Annet Naisanga; Ms. Sarah Nandudu; Ms. Hellen Onzia; Ms. Prossy Joy; Kalembe; Ms. Cissy Nabaterega; Ms. Edith Akello; Ms. Sylivia Kende; Ms. Ketty Atube Anena; Ms. Zaituna Kalinaki; Ms. Irene Nambozo; Dr. Martha Ajojo; Ms. Dorcus Imede; Ms. Betty Acen; Dr. Samuel Munduni; Ms. Caroline Muzaki; Ms. Evelyn Etulia; Ms. Joyce Kugonza; Ms. Mary Asiimwe; Ms. Ketty Akullo; Ms. Christine Atugonza; Ms. Lilian Kimuli; Ms. Jully Adokorach; Ms. Samuel Oboko Mugisa; Ms. Mary Andrutiru Asumpta; Ms. Viola Chebet; Ms. Immaculate Awoyo; Ms. Molly Eceru; Ms. Flavia Lindrio; Ms. Florence Namuddu; Ms. Lydia Nakitende; Ms. Violet Nakaweesi; Ms. Eleth Namulemo; Ms. Mary Anyango; Ms. Babrah Apedo; Ms. Peace Katushabe; Ms. Rebecca Birungyi; Ms. Florence Nakyambadde; Ms. Daphine Mukunde; Ms. Rhoda Mesiku; Ms. Lizzy Baako; Dr. Malon Isaac Atama; Ms. Mariam Shaban; Ms. Hellen Ayikoru; Ms. Sauda Musa; Ms. Agnes Busingye; Ms. Esther Alate; Ms. Sarah Adong; Ms. Judith Iculet; Ms. Proscovia Aisu; Ms. Phoebe Ojelel; Ms. Grace Amuge; Ms. Aisha Nassuna; Ms. Rashida Nalutaaya; Ms. Betty Nakamatte; Ms. Justine Jennifer Naggirinya; Ms. Rebecca Acakara; Ms. Esther Adeke;

Malawi Champions: Joyce Kanzingeni Hospital Attendant; Ernest Chipale Anaesthesia Clinical Technician; Jimmy Njiragoma Anaesthesia Clinical Technician; Kasiya Bauden Anaesthesia Clinical Technician; Emerson Masese Anaesthetic Technician; Patrick Chalirana Anaesthetic Technician; Paul Moyala Anaesthetic Technician; Rex Kusiwa Anaesthetic Technician; Rosina Banda Anaesthetic Technician; Themba Muthemba Anaesthetic Technician; Thom Chinguwo Anaesthetic Technician; Zione Alimasi Anaesthetic Technician; Everblessings Kamanga Anaesthetist; Gelevazio Kennedy Anaesthetist; Kingsley Malimusi Anaesthetist; Alice Mwandira Anesthetic Clinician; Eleson Kumbwemba Anesthetic Clinician; Alex Sembo Clinical Associate; Clophat Baleti Clinical Associate; Enock Chitokoto Clinical Associate; Enock Nakaole Clinical Associate; Samson Matandala Clinical Associate; Wongani Zgambo Clinical Associate; Aaron Chikuti Clinical Technician; Akuzike Chikoko Clinical Technician; Anthony Mpeya Clinical Technician; Emmanuel Nkonde Clinical Technician; Faith Nsonga Clinical Technician; Francisco Makuwira Clinical Technician; Fredrick Kapinga Clinical Technician; Grace Kanyenga Clinical Technician; James Kachingwe Clinical Technician; Kelvin Bowa Clinical Technician; Kondwani Lutepo Clinical Technician; Kumbukani Phiri Clinical Technician; Leonard Banda Clinical Technician; Luka Kwame Gondwe Clinical Technician; Mariam Chitwaga Clinical Technician; Michelle Kamwendo Clinical Technician; Mwayi Luka Clinical Technician;

Olhakalhalha Nayupe Clinical Technician; Onesimus Chipula Clinical Technician; Prince Katchika Clinical Technician; Rhitta Mwale Clinical Technician; Richard Nyirenda Clinical Technician; Rijasi Baloyi Clinical Technician; Shaliah Phiri Clinical Technician; Steve Dimba Clinical Technician; Tendai Simwaka Clinical Technician; Vincent Banda Clinical Technician; Yohane Chisavu Clinical Technician; Alicy Khonje District Medical Officer; Angelo Mwabungulu District Medical Officer; Dr Dennis Solomon District Medical Officer; Grace Chikonga District Medical Officer; Steria Mlepo District Medical Officer; Victor Kumfunda District Medical Officer; William Chimsanga District Medical Officer; Yakobe Machira District Medical Officer; Beatrice Kaluwa District Nursing and Midwifery Officer; Ndindase Kamanga District Nursing and Midwifery Officer; Patricia Kapena District Nursing and Midwifery Officer; Phyllis Baluwa District Nursing and Midwifery Officer; Tinamwabi Msiska District Nursing and Midwifery Officer; Aidah Banda Hospital Attendant; Alice Madeya Hospital Attendant; Annie Thom Hospital Attendant; Beatrice Mphuka Hospital Attendant; Cecelia Kalitsiro Hospital Attendant; Charity Mkandawire Hospital Attendant; Charles Mbenderana Hospital Attendant; Chrissy Chitsulo Hospital Attendant; Eggley Chimula Hospital Attendant; Elizabeth Masala Hospital Attendant; Evelyn Zgambo Hospital Attendant; Gertrude Chimlambe Hospital Attendant; Gloria Banda Hospital Attendant; Judith Banda Hospital Attendant; Licy Sichinga Hospital Attendant; Rhoda Mkandawire Hospital Attendant; Teleza Chigona Hospital Attendant; Raphael Mzengo Laboratory Assistant; Alinafe Kulemeka Laboratory Technician; Chimango Chuma Laboratory Technician; Davis Kotakota Laboratory Technician; Felix Mkwinda Laboratory Technician; Frazer Malata Laboratory Technician; Herbert Chiumia Laboratory Technician; Jones Kadewere Laboratory Technician; Mphatso Mafunga Laboratory Technician; Patrick Banda Laboratory Technician; Steven Gonthi Laboratory Technician; Wellington Gillimon Laboratory Technician; Ndaipamo Khoviwa Laboratory Technologist; Mercy Sandras Maternity Attendant; Wamaka Msopole Medical Doctor; Chisomo Mhura Medical Officer; Henry Mwakalinga Medical Officer; Leonard Naphazi Medical Officer; Stanley Ndhlov Medical Officer; Alepher Khombe Nurse Midwife Technician; Babra Mpingen Nurse Midwife Technician; Brenda Nekhoma Nurse Midwife Technician; Catherine Mohani Nurse Midwife Technician; Cecelia Nakhwema Nurse Midwife Technician; Christopher Mkaka Nurse Midwife Technician; Felix Mbamila Nurse Midwife Technician; Fwasani Chavula Nurse Midwife Technician; Jeromy Mwera Nurse Midwife Technician; Jotex Gambatula Nurse Midwife Technician; Karren Phillip Nurse Midwife Technician; Limbani Kadzuwa Nurse Midwife Technician; Lucy Masebo Nurse Midwife Technician; Lydia Mkandawire Nurse Midwife Technician; Maureen Hausi Nurse Midwife Technician; Mervis Mazemgera Nurse Midwife Technician; Mike Chiwanda Nurse Midwife Technician; Patricia Mwanja Nurse Midwife Technician; Sekanawo Kandindi Nurse Midwife Technician; Susan Kapalamula Nurse Midwife Technician; Ulemu Jere (Posthumously) Nurse Midwife Technician; Wezzie Kamanga Nurse Midwife Technician; Ethel Yassin Nurse Midwife; Grace Chipanda Nurse Midwife; Iron Poloto Nurse Midwife; Loveness Tchereni Nurse Midwife; Nelie Mwale Nurse Midwife; Pemphero Kafukiwe Nurse Midwife; Phonia Kavalo Nurse Midwife; Sella Shaba Nurse Midwife; Alex Chibaya Nursing and Midwifery Officer; Blessings Gondwe Nursing and Midwifery Officer; Chancy Chirwa Nursing and Midwifery Officer; Chimwemwe Kamuyalo Nursing and Midwifery Officer; Christina Bizwick Nursing and Midwifery Officer; Dalitso Zolowere Nursing and Midwifery Officer; Elizabeth Amini Nursing and Midwifery Officer; Gift Masiye Nursing and Midwifery Officer; Innocent Chirwa Nursing and Midwifery Officer; Janet Wister Nursing and Midwifery Officer; Kain Nyambalo Nursing and Midwifery Officer;

Lucia Jonasi Nursing and Midwifery Officer; Lydia Lipende Nursing and Midwifery Officer; Mathews Mtemang'ombe Nursing and Midwifery Officer; Morton Baloyi Nursing and Midwifery Officer; Naomi Thauzeni Nursing and Midwifery Officer; Philomeena Muragijimuna Nursing and Midwifery Officer; Raisa Likhaluwe Nursing and Midwifery Officer; Ruth Makwinja Nursing and Midwifery Officer; Syna Mposa Nursing and Midwifery Officer; Tabitha Mikeka Nursing and Midwifery Officer; Tamara Katumbi Nursing and Midwifery Officer; Tausley Kondowe Nursing and Midwifery Officer; Thandizo Baleti Nursing and Midwifery Officer; Thokozani Masiye Nursing and Midwifery Officer; Treazer Jere Nursing and Midwifery Officer; Ulemi Munthali Nursing and Midwifery Officer; Vaida Chiwaya Nursing and Midwifery Officer; Valentina Nkhoma Nursing and Midwifery Officer; Vitumbiko Mukhola Nursing and Midwifery Officer; Catherine Papalawo Nursing Auxilliary; Gladson Chima Nursing Officer; Isaac Zeka Nursing Officer; Maureen Chilikutali Nursing Officer; Norah Maloya Nursing Officer; Stella Chizumila Nursing Officer; Aidah Luwale Patient Attendant; Lucia Abraham Patient Attendant; Mercy Mkwezalamba Patient Attendant; Modester Njiragoma Patient Attendant; Rabecca Moyo Patient Attendant; Chikondi Harrison Pharmacist; Redson Dauya Pharmacist; Eckton Kumundayayi Pharmacy Assistant; Lewis Shafi Pharmacy Assistant; Peggie Gome Pharmacy Assistant; Sheriff Masiano Pharmacy Assistant; Andy Kishombe Pharmacy Technician; Charles Kalajira Pharmacy Technician; Chikondi Mkandawire Pharmacy Technician; Emmanuel Phiri Pharmacy Technician; Faith Simkonda Pharmacy Technician; Gift Chathyoka Pharmacy Technician; Jackson Mumba Pharmacy Technician; Lusungu Chiumia Pharmacy Technician; Mustafa Kim Pharmacy Technician; Clara Chikhawo Principal Nursing and Midwifery Officer; Enelesi Chingapa Principal Nursing and Midwifery Officer; Brave Mhera Registered Nurse Midwife; Christopher Mulolo Registered Nurse Midwife; Gift Mwandira Registered Nurse Midwife; James Phata Registered Nurse Midwife; Maureen Chisokola Registered Nurse Midwife; Patrick Ganizani Registered Nurse Midwife; Richard Abdul Senior Clinician; Patricia Magwaya Senior Nurse Midwife Technician; Barbara Ussein Thembakako Senior Nursing and Midwifery Officer; Esme Kamaliza Senior Nursing and Midwifery Officer; Grace Zakeyu Senior Nursing and Midwifery Officer; Ignasio Jowasi Senior Nursing and Midwifery Officer; Loveness Yuda Senior Nursing and Midwifery Officer; Madalitso Mwamlima Senior Nursing and Midwifery Officer; Mercy Kulanga Senior Nursing and Midwifery Officer; Patrick Baluwa Senior Nursing and Midwifery Officer; Silvester Mzumalo Senior Nursing and Midwifery Officer; Tamara Banda Senior Nursing and Midwifery Officer; Marriam Kalembo Ward Attendant;

Uganda Champions: Dr. Ponsiano Kiwanuka Consultant Obgyn; Dr. Joseph Beinomugisha Consultant Obgyn; Dr. Odur Andrew Consultant Obgyn; Dr. Acan Lily Rose Consultant Obgyn; Dr. Sarah Nakivumbi Consultant Obgyn; Dr. Jude Muloowa Consultant Obgyn; Dr. Kenneth Mugabe Consultant Obgyn; Dr. Emmanuel Onapa Consultant Obgyn; Dr. Alfred Francis Ogwang Consultant Obgyn; Dr. Kenneth Ekakoro Consultant Obgyn; Dr. Shaffi Dr.Hamuza Consultant Obgyn; Ms. Kauma Zaina Health Inspector; Mr. Ronald Wotti Hospital Administrator; Mr. Ramathan Gaboli Hospital Administrator; Mr. Peter Ochom Olaki Hospital Administrator; Ms Linda Shabera Ntongo Hospital Administrator; Mr. Robert Lukenge Hospital Administrator; Mr. Christopher Ongom Hospital Administrator; Mr. Robert Gatama IPC Focal person; Mr. David Kyobe IPC Focal person; Mr. Yorkman Ochom IPC Focal person; Dr. Frednald Byamugisha IPC Focal person; Ms Ann Aluo IPC Focal person; Dr. Allan Joel Okidi Medical Officer; Dr. Christopher Edwin Isiko Medical Officer; Dr. Hamisi Nsubuga Medical Officer; Dr. Pius Otim

Medical Officer; Dr. Joseph Katergga Medical Officer; Dr. Emmanuel Ssekyeru Medical Officer; Dr. Lambert Eragu Medical Officer; Dr. Clare Amen Medical Officer; Dr. Angella Margret Opus Medical Officer; Dr. Everest Tukamushaba Medical Officer; Dr. Simon Baguma Medical Officer; Dr. Miriam Patience Nakawoye Medical Officer; Dr. Lydia Namono Wataka Medical Officer; Dr. Godfery Muyomba Medical Officer; Dr. Thomas Ochar Medical Officer; Dr. Augustus Ogutu Medical Officer; Dr. Grace Namulondo Medical Officer; Mr. Christopher Amandu Microbiologist; Mr. Jerome Opio Pharmacy technician; Mr. Silvio Abiriga Principal Nursing Officer; Ms Jane Manano Principal Nursing Officer; Ms Ann Mary Nabwire Principal Nursing Officer; Ms Stella Agembi Principal Nursing Officer; Mr. Mark Anthony Omoding QI Focal person; Ms. Rose Aguparu Registered Midwife; Ms. Margret Obizuyo Registered Midwife; Ms. Knight Anicia Registered Midwife; Ms. Grace Adiru Registered Midwife; Ms. Laura Avako Diku Registered Midwife; Ms. Lydia Fungaro Registered Midwife; Ms. Jackyln Apoo Registered Midwife; Ms. Grace Oduru Registered Midwife; Ms. Deborah Anyait Registered Midwife; Ms. Cissy Atiang Registered Midwife; Ms. Sarah Nasirumbi Registered Midwife; Ms. Caroline Nanyondi Registered Midwife; Ms. Joseline Cheptengan Registered Midwife; Ms. Joyce Mukebezi Registered Midwife; Ms. Angella Anyu Registered Midwife; Ms. Petra Babirye Registered Midwife; Ms. Christine Muganzi Registered Midwife; Ms. Debrah Kyosiimire Registered Midwife; Ms. Hanifa Babirye Registered Midwife; Ms. Cissy Amoo Registered Midwife; Ms. Nangobi Phoebe Registered Midwife; Ms. Immaculate Ndyamuhaki Registered Midwife; Ms. Berine Acen Registered Midwife; Ms. Mariam Mbeiza Registered Midwife; Ms. Florence Matama Registered Midwife; Ms. Bena Emima Iyebu Registered Midwife; Ms. Sarah Nandudu Registered Midwife; Ms. Lydia Bangi Registered Midwife; Ms. Peninah Nabaasa Registered Midwife; Ms Jovia Nakasaga Registered Midwife; Ms Betty Nagudi Registered Midwife; Ms Christine Nakiggude Registered Midwife; Ms Sylvia Nakato Registered Midwife; Ms Mary Nagujja Registered Midwife; Ms Victoria Namutebi Registered Midwife; Ms Joyce Atim Registered Midwife; Ms Joyce Imalingat Registered Midwife; Ms Zaituni Namulumba Registered Midwife; Ms Allen Mudoola Registered Midwife; Ms Florence Nanyonjo Registered Midwife; Ms Monica Akello Registered Midwife; Ms Abongo Evelyne Registered Midwife; Ms Ajambo Catherine Registered Midwife; Ms Agnes Bwekembe Registered Midwife; Ms Grace Atim Registered Midwife; Ms Lucy Nyadoi Registered Midwife; Ms Susan Nakate Registered Midwife; Ms Florence Mbazalidde Registered Midwife; Ms Ruth Nakawungu Registered Midwife; Ms Beatrice Mirembe Registered Midwife; Ms Mary Gorreti Acipa Registered Midwife; Ms Jane Akello Registered Midwife; Ms Tabitha Asago Registered Midwife; Ms Yudaya Alunga Registered Midwife; Ms Hadijjah Nnalukwago Registered Midwife; Ms Rebecca Wanyana Registered Midwife; Ms Joyce Namayanja Registered Midwife; Ms Margaret Nambi Registered Midwife; Ms Christine Namutebi Registered Midwife; Ms Harriet Nakanaabi Registered Midwife; Ms Betty Apio Registered Midwife; Ms Christine Gasi Registered Midwife; Ms Susan Nakami Registered Midwife; Ms Judith Ayuru Registered Midwife; Ms Irene Abeja Acheka Registered Midwife; Ms Betty Nakacwa Registered Midwife; Ms Sarah Ogobi Registered Midwife; Ms Nuulu Nalubega Registered Midwife; Ms Fatuma Ramadhan Noha Registered Midwife; Ms Mabuya Sinan Registered Midwife; Ms Halima Nalunga Registered Midwife; Ms Vivian Ntongo Registered Midwife; Ms Dorothy Namazzi Registered Midwife; Ms Zaitun Nyanjura Registered Midwife; Ms Susan Adoch Registered Midwife; Ms Gloria Ogwal Registered Midwife; Ms Agnes Akello Registered Midwife; Ms Penlope Kabasomi Registered Midwife; Ms Majory Mbabazi Registered Midwife; Ms Winifred Namuswe Registered Midwife; Ms Rukia Akello Registered Midwife; Ms

Sarah Nandutu Registered Midwife; Ms Lukia Kabitanya Registered Midwife; Ms Florence Mutyembu Registered Midwife; Ms RoseMary Lunyoro Registered Midwife; Ms Elizabeth Alibo Registered Midwife; Ms Elizabeth Adiba Registered Midwife; Ms Edith Kamisya Registered Midwife; Ms Edith Mutuka Registered Midwife; Ms Christine Janet Mugala Registered Midwife; Ms Harriet Atimango Registered Midwife; Ms Charity Pikwo Registered Midwife; Ms Mercy Lamwaka Registered Midwife; Ms Agnes Akumu Registered Midwife; Ms Florence Drateru Registered Midwife; Ms Juliet Ademun Registered Midwife; Ms Magdalene Iraat Registered Midwife; Ms Nancy Lanyero Registered Midwife; Ms Jackline Atikuru Registered Midwife; Ms Harriet Milly Akoth Registered Midwife; Ms Kevin Isiru Registered Midwife; Ms Stella Chelangati Registered Midwife; Ms Nancy Acan Registered Midwife; Ms Jamila Candiru Registered Midwife; Mr. Kizito Mugaya Registered Nurse; Mr. Joel Bogere Registered Nurse; Mr. Ronald Kibira Registered Nurse; Ms. Nangobi Phoebe Registered Nurse; Mr. Francis Musayi Registered Nurse; Mr. Francis Omach Registered Nurse; Ms. Susan Rose Alero Registered Nurse; Mr. Arthur Masika Registered Nurse; Mr. Isaac Ecungo Registered Nurse; Mr. Allan Paul Nsubuga Registered Nurse; Mr. Herbert Kyamanywa Registered Nurse; Ms Dina Kadondi Registered Nurse; Mr. Gerald Gundu Registered Nurse; Mr. Peter Ojok Registered Nurse; Mr. Godfrey Tuyisenge Registered Nurse; Mr. Rodgers Maraka Registered Nurse; Mr. Stephen Adiga Registered Nurse; Mr. Angelo Drate Registered Nurse.

Details of the APT-Sepsis Intervention and Implementation Strategy

The Active Prevention and Treatment of Maternal Sepsis (APT-Sepsis) program is a multicomponent intervention that seeks to support healthcare providers to achieve three central goals that will reduce maternal infection-related adverse outcomes.

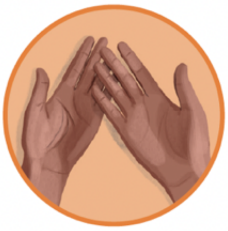

Goal 1:

Hand Hygiene

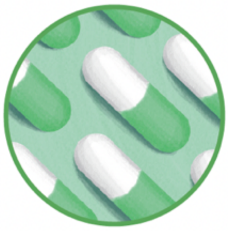

Goal 2:

Infection Prevention and Treatment

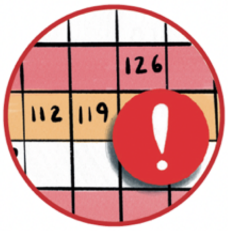

Goal 3:

Sepsis Detection and Management

The APT-Sepsis goals

This appendix provides additional information on the intervention content, the implementation strategy, and the rationale underpinning the program's design.

All materials and additional implementation details are available online at:

www.apt-sepsis.org

Or through the WHO Global Maternal and Neonatal Sepsis Initiative at:

www.srhr.org/sepsis/apt-sepsis-programme/

Intervention description:

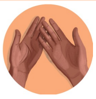

Goal 1: Hand hygiene

- Adhere to WHO 5 moments of hand hygiene
- Correct hand hygiene technique

Healthcare workers should handwash with soap or cleanse with alcohol based handrub their hands:

1) Before touching the woman or newborn,
2) Before a clean or aseptic procedure,
3) After body fluid exposure risk,
4) After touching a woman or newborn,
5) After touching a woman or newborn's surroundings.

When not to use handrub:
- Caring for patients with diarrhoea
- Having visibly soiled hands
- After personal toilet use

- Effective handwashing and handrub following the WHO technique
    - Handwash for 40-60 seconds, using 6 movements
    - Handrub for 20-30 seconds, using 6 movements

- When to use standard/sterile gloves
- Training included an ultraviolet handrub/lightbox practical to practice and feedback on technique

Working environment materials:
- WHO hand hygiene posters in key positions in the working environment:
    - All clinical areas
    - Next to handwashing stations
    - In toilet facilities
- Handrub and soap supported in facilities with low supply
- Simple bucket and tap based handwash stations installed in critical areas when absent

Example WHO posters used for APT-Sepsis Goal 1

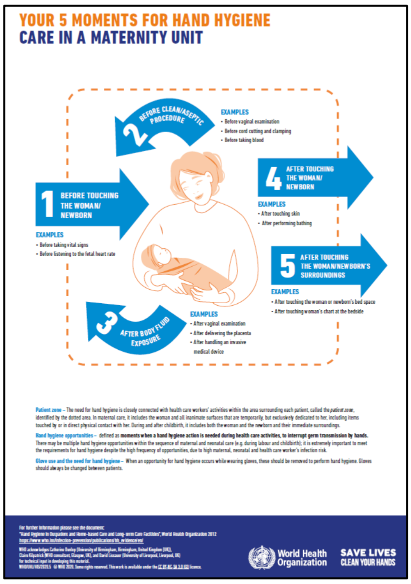

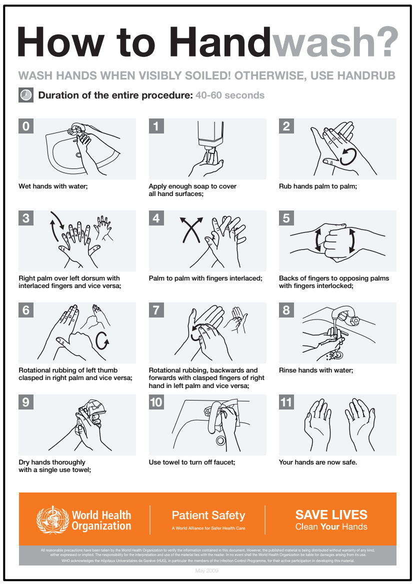

Goal 2: Infection Prevention and Treatment

- Use antibiotics for prophylaxis and treatment as recommended by the WHO
  - Wash the skin and vagina with antiseptic prior to caesarean section

- Appropriate use of antibiotics for prophylaxis
- WHO recommendations for safe clinical practice
- Prompt diagnosis of infection
- National guidelines for infection treatment

When to use prophylaxis:
- Preterm pre-labour rupture of membranes
- Manual removal of the placenta
- Abortion or miscarriage surgery
- Operative vaginal birth
- 3rd or 4th degree perineal tears.
- Prior to caesarean section
- Vaginal Group B *Streptococcus* colonisation

When not to use prophylaxis:
- Uncomplicated pregnancy or birth
- Pre-term labor with intact membranes
- Meconium-stained amniotic fluid
- Episiotomy

- **Caesarean section prophylaxis:** Single does of cephalosporin, prior to skin incision. No routine antibiotics after surgery.

- **WHO recommendations for safe clinical practice**:
  - Skin preparation with alcohol-based chlorhexidine gluconate where available
  - Vaginal preparation with povidone-iodine or chlorhexidine gluconate immediately prior to caesarean section
  - Limit vaginal examination in the first stage of active labour and do not routinely shave the pubic area before delivery

- Training included a vaginal preparation practical with gynaecological mannequins

Working environment materials:
- Antibiotic treatment recommendations according to national guidelines were provided on the APT-Sepsis gestation wheel
- Posters were visible in key positions in the working environment:
  - Clinical areas and dispensaries (antibiotic treatment guidelines)
  - Labour ward and delivery suite (vaginal preparation instructions)

Example poster for APT-Sepsis Goal 2. Describing technique of vaginal preparation

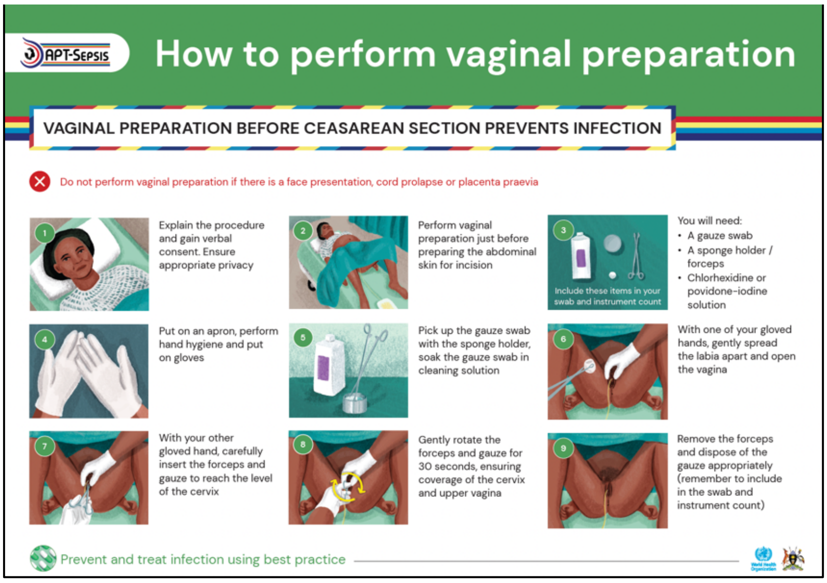

Goal 3: Sepsis Detection and Management

“Suspect sepsis, start FAST-M”

Early sepsis detection:
A structured modified early obstetric warning system (MEOWS) chart
FAST-M bundle:
Use bundled treatment for sepsis with **f**luids, **a**ntibiotics, **s**ource identification and control, **t**ransfer and **m**onitoring

Early sepsis detection:
- Staff trained to complete MEOWS chart for every patient, minimum daily use

Red flags include - Respiratory rate; 25 or more; Heart rate 120 or more; Systolic blood pressure; 89mmHg or less; Diastolic blood pressure 39mmHg or less; Urine; Less than 0.5ml per hour; Altered mental state.

Suspected infection + Reg flag = Triggers FAST-M bundle

- Decision tool to guide escalation of care following red and yellow flags

- **FAST-M Bundle**: How and when to initiate the FAST-M bundle of care:
  - Fluids : 500ml crystalloid bolus, repeated if hypotension persisted
  - Antibiotics: according to source; or if unknown then ceftriaxone 2g IV OD and metronidazole 500mg IV TDS/400mg PO TDS with an additional singe dose of gentamicin 5mg/kg IV if haemodynamically unstable
  - Source: Identify and remove/treat source of infection
  - Transfer: Consider if a different hospital or location is required
  - Monitoring: repeat maternal observations every 30 minutes until stable, with neonatal monitoring and review as required

Working environment materials:
- Posters were visible in key positions in the working environment:
  - All clinical areas (decision tool and FAST-M bundle of care)
- Paper MEOWS charts were provided for all patients
- Paper-based decision tools and paper FAST-M bundle checklists were available in all clinical settings

Example posters for APT-Sepsis Goal 3

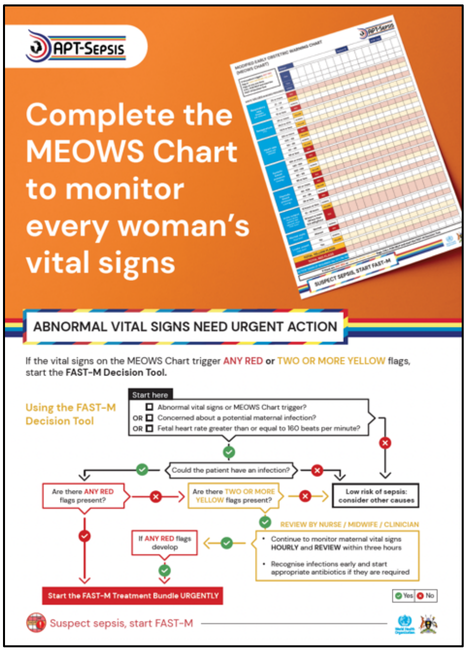

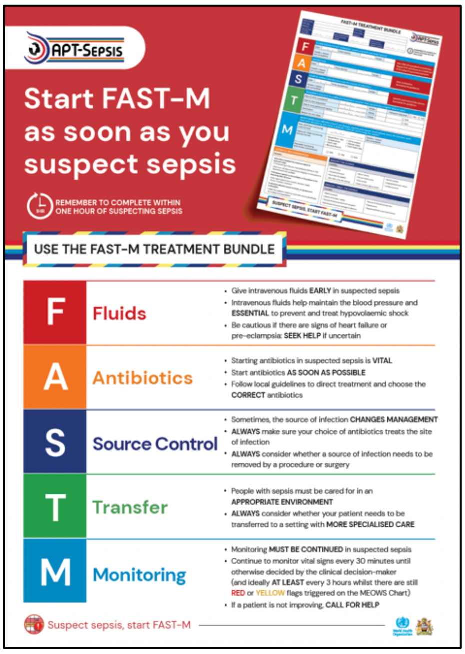

MODIFIED EARLY OBSTETRIC WARNING SYSTEM CHART (MEOWS CHART)

APT-Sepsis

If the patient triggers ANY RED or TWO OR MORE YELLOW flags at any one time, CALL FOR HELP and start the FAST-M Decision Tool.

Patient		Patient ID		DOB / Age	

WRITE VALUES IN BOXES PROVIDED

		Date															
		Time															
		Initials															
Respiratory rate (breaths per minute)	25 or more	RED															
	21 - 24	YELLOW															
	11 - 20	NORMAL															
	10 or less	RED															
Temperature (°C)	38 or more	YELLOW															
	36.0 to 37.9	NORMAL															
	35.9 or less	YELLOW															
Heart rate (beats per minute)	120 or more	RED															
	100 - 119	YELLOW															
	50 - 99	NORMAL															
	40 - 49	YELLOW															
	39 or less	RED															
Systolic blood pressure (mmHg)	160 or more	RED															
	140 -159	YELLOW															
	100 -139	NORMAL															
	90 - 99	YELLOW															
	89 or less	RED															
Diastolic blood pressure (mmHg)	110 or more	RED															
	90 -109	YELLOW															
	40 - 89	NORMAL															
	39 or less	RED															
Urine output Hours since patient last passed urine (tick box)	12 hours or less	NORMAL															
	12 - 18 hours	YELLOW															
	18 hours or more OR less than 0.5 ml/kg/hour	RED															
Mental state (tick box)	Normal	NORMAL															
	Altered	RED															
Looks unwell (tick box)	No	NORMAL															
	Yes	YELLOW															
TOTAL YELLOW FLAGS																	
TOTAL RED FLAGS																	
ACTION TAKEN (IF REQUIRED) Yes (Y) / No (N)																	

If there are ANY RED or TWO OR MORE YELLOW flags, CALL FOR HELP and start the FAST-M Decision Tool.

SUSPECT SEPSIS, START FAST-M

World Health Organization

Modified Early Obstetric Warning System (MEOWS) chart

(Malawian Ministry of Health Logo Version)

FAST-M DECISION TOOL

APT-SEPSIS

Patient name		Staff name	
DOB / Age		Role / Cadre	
Patient ID		Signature	
Date	_____ /_____ /_____	Time	______ : ______

START HERE

- ☐ Abnormal vital signs or MEOWS Chart trigger?
  (Respiratory rate / Temperature / Heart rate / Blood pressure / Urine output / Mental state / Looks unwell)
- OR ☐ Concerned about a potential maternal infection?
- OR ☐ Fetal heart rate of 160 beats per minute or more

COULD THE PATIENT HAVE AN INFECTION?

PELVIS	ABDOMEN	CHEST	WOUND	OTHER
☐ Offensive vaginal discharge<br>☐ Vaginal bleeding<br>☐ Delay in uterine involution	☐ Abdominal pain<br>☐ Urinary symptoms<br>☐ Vomiting / diarrhoea	☐ Cough / shortness of breath / sore throat<br>☐ Breast erythema / pain	☐ Discharging wound / wound dehiscence<br>☐ Swollen / painful cannula site	☐ Fever / rigors / malaise<br>☐ Headache / neck stiffness / rash<br>☐ Other:

ARE ANY SEPSIS RED FLAGS PRESENT?

- ☐ **Respiratory rate** 25 breaths per minute or more
- ☐ **Heart rate** 120 beats per minute or more
- ☐ **Systolic blood pressure** 89 mmHg or less
- ☐ **Diastolic blood pressure** 39 mmHg or less
- ☐ **Not passed urine** in over 18 hours (less than 0.5 ml/kg/hr if catheterised)
- ☐ **Mental state** Not alert

ARE TWO OR MORE SEPSIS YELLOW FLAGS PRESENT?

- ☐ **Respiratory rate** 21 - 24 breaths per minute
- ☐ **Temperature** 35.9 °C or less OR 38°C or more
- ☐ **Heart rate** 100–119 beats per minute
- ☐ **Systolic blood pressure** 90 - 99 mmHg
- ☐ **Last passed urine** 12 - 18 hours ago
- ☐ **Looks unwell**

REVIEW BY NURSE / MIDWIFE / CLINICIAN

Continue to monitor maternal vital signs HOURLY and REVIEW the patient within three hours

Review taken place within three hours? ☐ YES ☐ NO

Date ___ /___ /___ Time ____ : ____

Recognise infections EARLY and start appropriate antibiotics.

Are antibiotics required? ☐ YES ☐ NO

IF ANY RED FLAGS DEVELOP

START FAST-M TREATMENT BUNDLE NOW

Urgent review by nurse / midwife / clinician and take action within ONE HOUR

LOW RISK OF SEPSIS

- Review and manage appropriately: treat non-severe infections early to prevent sepsis.
- Continue to monitor inpatients using the MEOWS Chart.
- Educate patients on warning signs of infection when discharged.

SUSPECT SEPSIS, START FAST-M

Version 1.1
19/DEC/2023

World Health Organization

APT-Sepsis FAST-M decision tool (Malawian Ministry of Health Logo Version)

FAST-M TREATMENT BUNDLE

APT-Sepsis

Patient name		Staff name	
DOB / Age		Role / Cadre	
Patient ID		Signature	

Date and time of red flag observation	___/___/___ ___:___	Date & time FAST-M Treatment Bundle started	___/___/___ ___:___	Date & time of review by nurse / midwife / clinician	___/___/___ ___:___

REMEMBER TO COMPLETE THESE ACTIONS WITHIN ONE HOUR

F — FLUIDS (caution in pre-eclampsia, severe anaemia and heart failure)

Date	___/___/___	Time started	____:____	Initials		Give 500 ml crystalloid immediately. Repeat 500 ml boluses to a maximum of 30 ml/kg if hypotension persists
Details / reason not completed						

A — ANTIBIOTICS

Date	___/___/___	Time started	____:____	Initials		Give antibiotics. See below for guidance
Details / reason not completed						

S — SOURCE control (identify and treat the source of infection)

Date	___/___/___	Time considered	____:____	Initials		Identify and control the source. See below for guidance
Details / reason not completed						

T — TRANSFER if required (to a different hospital or location that can provide a higher level of care)

Date & time considered	___/___/___ ___:___	Initials		Transport required	☐ YES ☐ NO
Date & time requested	___/___/___ ___:___	Initials		☐ N/A	
Date & time patient left facility	___/___/___ ___:___	Initials		☐ N/A	
Destination					
Reason for any delay					

M — MONITORING (start MEOWS Chart if not already started and repeat observations every 30 minutes, until otherwise decided by the nurse / midwife / clinician performing the review)

Date and time monitoring commenced:	___/___/___ ____:____	Details / reason not completed
Maternal / fetal monitoring should include:	• Respiratory rate • Temperature • Heart rate • Blood pressure • Urine output • Mental state • Fetal heart rate	
Neonatal monitoring and review commenced:	☐ YES ☐ NO ☐ N/A	

ANTIBIOTIC RECOMMENDATION

Consider:

Immediate treatment for maternal sepsis of unknown origin:

- Ceftriaxone 2g IV OD plus metronidazole 500mg IV TDS
- Add a one-off dose of gentamicin 5mg/kg IV if the patient is haemodynamically unstable

If the above regimen is not available or the patient is not improving after 48 hours:

- Seek urgent advice from a senior decision-maker (nurse / midwife / clinician)

If maternal infection source is known, or as soon as it is identified:

- Adapt the antibiotic choice to cover that source specifically, according to local guidelines

IDENTIFY THE SOURCE

Consider:

- Clinical history
- Clinical examination
- Blood tests (if available) (FBC, U&Es, LFTs, CRP, clotting)
- Blood cultures
- HIV and malaria tests
- Urine sample
- Swabs (wound, vagina, throat)
- Sputum sample
- Imaging (abdominal / chest)
- Lumbar puncture

REMOVE / TREAT THE SOURCE

Consider:

- Malaria treatment
- Delivery of the baby / babies
- Removal of retained products of conception
- Debridement of wound / drainage of collection
- Removal of infected cannula / line
- Hysterectomy
- Targeted antibiotics once source known

SUSPECT SEPSIS, START FAST-M

World Health Organization

APT-Sepsis FAST-M bundle (Malawian Ministry of Health Logo Version)

Implementation strategy:

The implementation approaches were developed from a behavior change perspective, to target the capabilities, opportunities and motivation to improve intervention uptake (COM-B).[15] Key components were: hospital leadership engagement; program champions selected from existing facility staff; multi-disciplinary, on-site training with comprehensive training materials (manuals, flipcharts, presentations, videos and handwash and vaginal preparation practice equipment); paper based tools (observation charts, decision aids and a FAST-M checklist); and performance feedback with dashboards and quarterly visits. Its design reflects extensive formative research, stakeholder engagement, and a commitment to sustainability and scalability.

Hospital leadership engagement

The delivery of the APT-Sepsis program utilized teams at health facility-level. Local facility leadership team engagement was important to ensure they understood, endorsed and facilitated systems change, creating a supportive environment for implementation. Leadership continued to be engaged at quarterly site visits. Each intervention facility's local leadership identified their Champions according to a description of the role and characteristics required.

Central Champion training

APT-Sepsis utilized a train-the-trainer design, with facility-level Champions first being trained by the study's country-level hub teams, who then supported Champions to deliver multi-professional APT-Sepsis training and mentorship to their facility's healthcare staff.

Each facility selected 5-12 champions based on the facility size and needs. Champions were selected from a diverse range of cadres including nurses, midwives, doctors, clinical officers, pharmacists, and laboratory personnel. They came from the key clinical areas requiring implementation including wards, delivery suite and theatres. They did not receive additional payments for this role.

The training of APT-Sepsis Champions was designed to equip Champions with the knowledge, skills and tools needed to deliver the APT-Sepsis training to maternal health staff at their own facilities. Champions were invited to a centralized four day event, led by the country's hub team. Each Champion received the APT-Sepsis manual, describing the program and the facility-level training structure in detail, and a resource guide, which described their role, the intervention materials provided by the program and how each of these fed into achieving APT-Sepsis's goals. If new Champions were identified during the course of the program's delivery, they were trained by local Champions or the hub team.

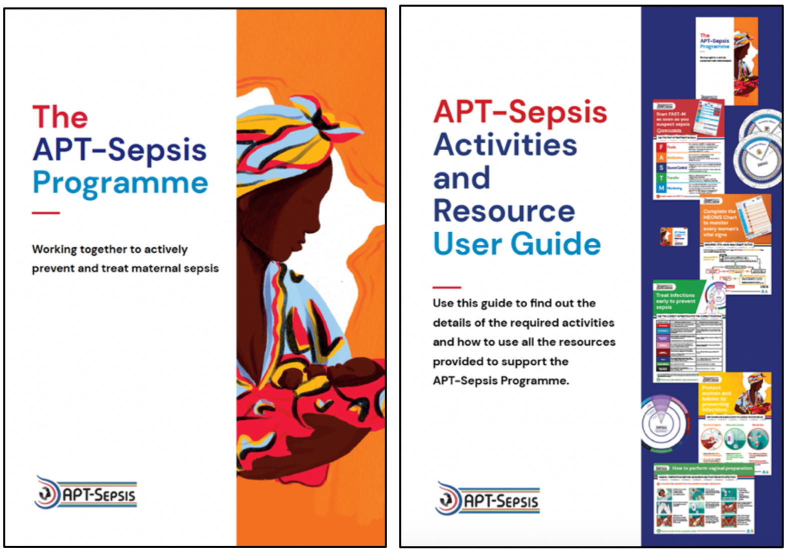

APT-Sepsis manual and activities and resource guide received by Champions

Facility-level training and mentorship

After the centralized Champion training, the train-the trainers-model ensured it was their responsibility to cascade APT-Sepsis training to their home facility's maternal health team.

The Champions led a two day training event for staff at facility-level. This training was run twice during the same week to ensure as many staff as possible had the opportunity to attend without disrupting normal clinical staffing. Training was offered to all staff who provided care for women during or after pregnancy. The training was led by the local team of APT-Sepsis Champions with the support of their local hub team, and this activity occurred during the 3 month transition phase of the trial. During the two-day course, eight modules were delivered, each supported by presentations which could be delivered by computer based presentations or flipcharts, and videos. There were practical sessions for hand hygiene and vaginal preparation.

Following this training, attendees all received a certificate of attendance endorsed by the Ministry of Health (MoH) in each country which contributed to their Personal Development Plans (PDP) for self-development.

Staff were each issued with a pocket guide which provided key summary information and an obstetric gestation wheel with a reverse that contained a guide to the use of antibiotics as per the local MoH requirements. Training also included task-shifting strategies, which were training patient attendants to monitor vital signs and empower nurse-midwifes to initiate the FAST-M bundle based on the decision aid algorithm without needing to wait for escalation.

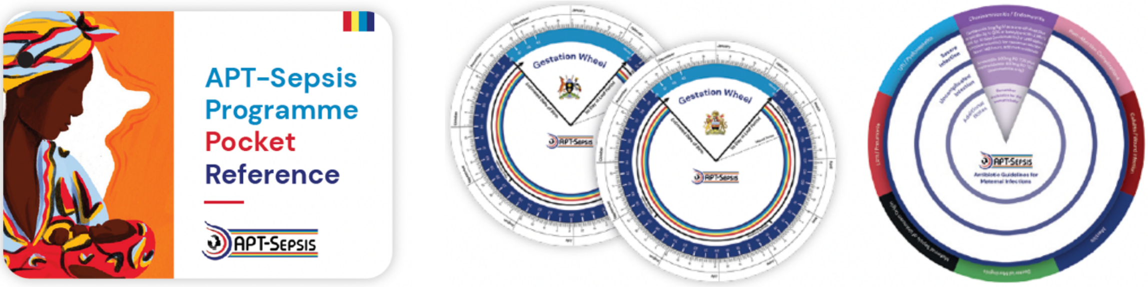

APT-Sepsis pocket reference and gestation wheel received by healthcare staff.

After the training, champions offered ongoing mentorship, coaching and performance monitoring, as well as onboarding for new staff during the program's delivery. This approach supported sustained implementation given ongoing staff turnover, as whole facility training was not repeated. The champions served as APT-Sepsis role models and advocates, and provided leadership for continuous improvement. Champions did not receive additional payment for this role.

Implementation tools

In addition to the materials for training and for healthcare providers, each intervention facility was provided with a package of APT-Sepsis materials for the working environment to support daily practice, listed previously in the intervention content (posters, MEOWS charts, decision tools and FAST-M bundle checklist). Soap and alcohol based hand rub was provided where sites did not have an adequate supply, resupply was provided if required.

Other site support

A small number of thermometers and blood pressure machines were supplied to all sites where they were needed, but only on one occasion. Key consumables, such as antibiotics and IV fluids, remained locally sourced through regular procurement. The trial did not provide additional staffing resources. Patient transport to higher level facilities was provided through the standard health system approaches.

Dashboards and feedback visits

Sites were provided with monthly and quarterly dashboard sheets showing their local implementation performance across the three goals. This was reinforced at quarterly hub team review visits. The hub team had access to a centralized electronic dashboard that showed all sites.

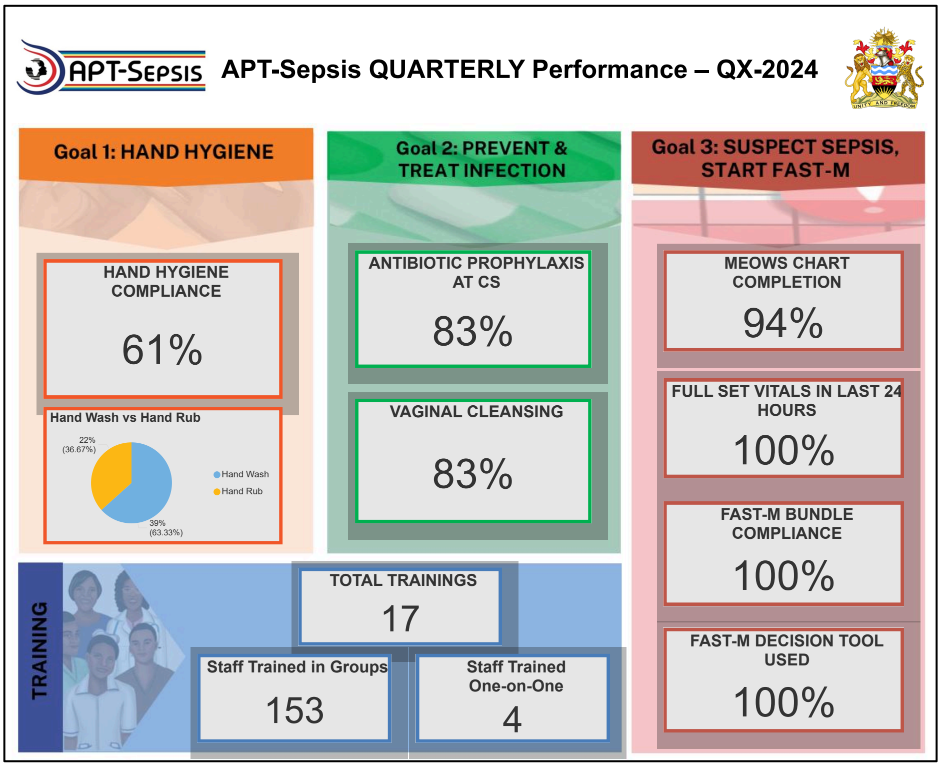

APT-Sepsis dashboard example.

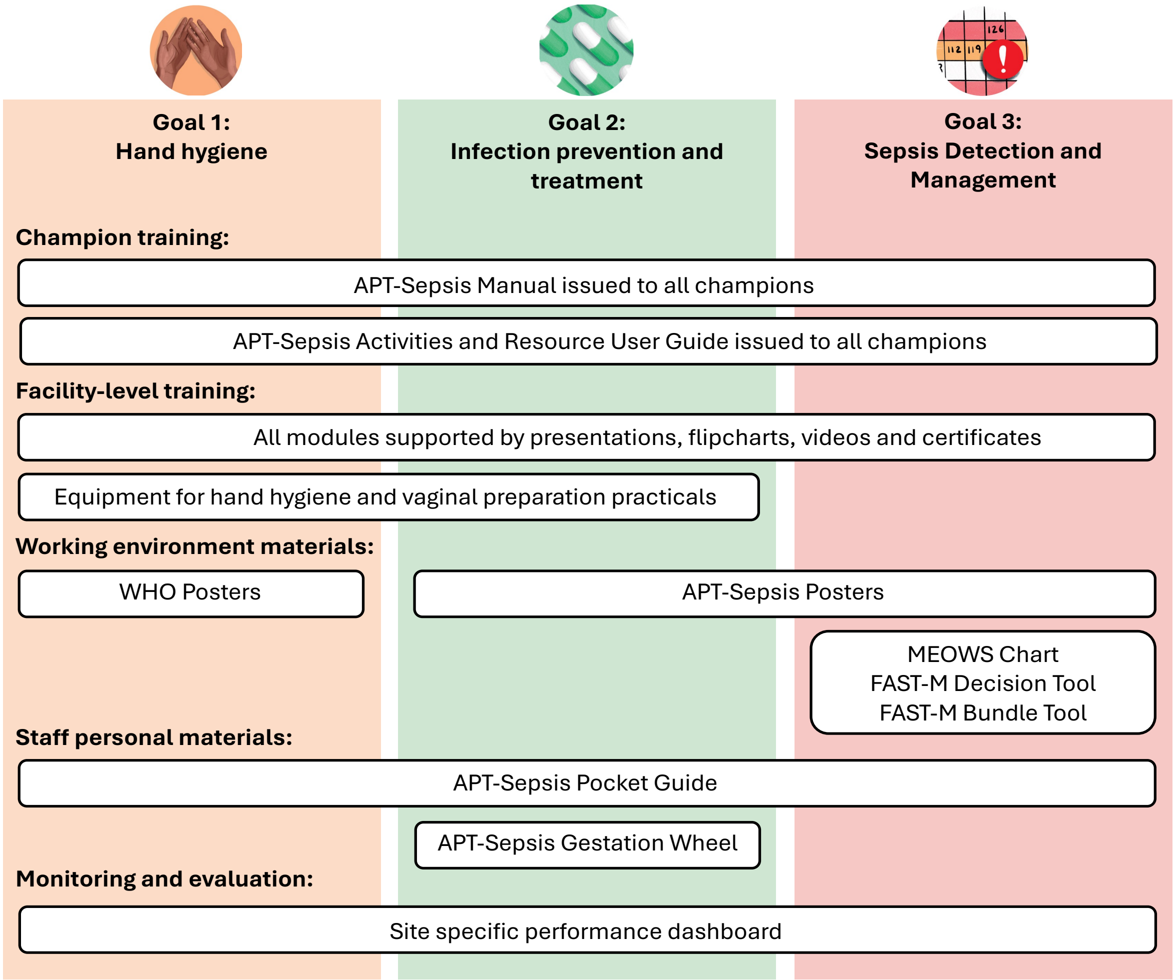

APT-Sepsis materials summary, mapped to the three goals.

<u>Design rationale:</u>

Goal 1

Adaptation of the WHO hand hygiene toolkit: we worked with the WHO Infection Prevention and Control Global Unit, conducting mixed-methods research to adapt the WHO hand hygiene posters and the WHO multi-modal hand hygiene improvement strategy for the maternity setting.[1-3]

Hand hygiene pilot studies in Malawi and Uganda: we conducted a pilot study across three health facilities in Malawi (these hospitals were not included in the APT-sepsis study) testing the introduction of the adapted WHO multi-modal hand hygiene improvement strategy alongside the other components of APT-sepsis. This allowed optimization of the materials and implementation strategy.[4]

Goal 2

Infection prevention and management evidence synthesis: a diverse group of experts convened by the WHO identified key questions and interventions for preventing and treating maternal peripartum infections. The most important interventions were examined through 24

systematic reviews, These provide the evidence base for 20 WHO recommendations in the "WHO recommendations for prevention and treatment of maternal peripartum infections",[5] with four updated recommendations subsequently issued as new evidence emerged.[6-9] These recommendations form the key evidence base for goal 2 of the intervention.

Operationalization of WHO recommendations: co-designed training materials and tools were developed to support implementation and compliance with the recommendations from the WHO peripartum infection guidelines.[5] Specific elements include pocket sized aide memoires, an adapted gestation wheel tool to improve rational antibiotic prescribing and reminder posters. These materials were piloted and optimized alongside the other components of APT-Sepsis in three hospitals in Malawi using a mixed-methods approach.[4]

Goal 3

Development of a bundle of care (FAST-M) for initial management of maternal sepsis: a robust approach was taken to develop a maternal sepsis bundle (the FAST-M bundle), optimizing it specifically for a maternal population in a low resource setting. The development process consisted of evidence synthesis to identify possible bundle components, followed by a modified Delphi approach, and an international expert panel. Three rounds of consultations were undertaken, then combined with the feedback from in-person meetings of the WHO Maternal Sepsis Working Group and a concluding workshop in Malawi. A clear consensus was reached through this process on the appropriate bundle components (fluids, antibiotics, source control, transfer and monitoring), which were subsequently summarized with the acronym FAST-M to assist practitioner recall.[10]

Feasibility study of sepsis identification tools and the FAST-M bundle: the FAST-M bundle has been evaluated in Malawi. This evaluation involved a multi-center, mixed-methods, before and after design feasibility study, conducted in 15 health facilities (which were not included in APT-Sepsis). The sites demonstrated improvements in the detection and management of maternal sepsis but the effect on clinical outcomes remains uncertain.[11] A qualitative evaluation (35 semi-structured interviews and nine focus groups) provided insights into the process of implementation, that were integrated into the APT-sepsis program. There was further learning as FAST-M was also adapted and implemented in Pakistan.[12-14]

APT-Sepsis program co-design

Following these formative processes, the integration and refinement of materials was undertaken through a further co-design process. This included regular team meetings including both Malawian and Ugandan clinicians and researchers and presentations to key stakeholders including representatives of the MoH in both Malawi and Uganda, the WHO, and the APT-Sepsis Trial Management Group. Each set of materials underwent three review cycles prior to production.

There was additional engagement and advice at multiple consultations before and during the trial from healthcare providers and service users and community representative through patient and public involvement and engagement groups at the Malawi Liverpool Wellcome Research Programme, Malawi and the Infectious Diseases Institute, Uganda.

Study Outcome Definitions

Adapted WHO Near Miss Criteria

Maternal near miss: A woman who nearly died but survived a complication that occurred during pregnancy, childbirth or postpartum up to 42 days.

The WHO near-miss criteria have been adapted for the purposes of the APT-Sepsis trial to ensure their ascertainment will not be influenced by the intervention. Criteria which are reliant on appropriate completion of vital sign observations, diagnostic tests or treatments that are susceptible to variability based on site practices, performance and treatment thresholds have been excluded to reduce measurement bias.

The criteria were unchanged throughout the study.

Individuals in whom these signs / symptoms or outcomes are not identified or reported were assumed not to have had a ‘near miss’.

Operational definitions

The following criteria define a maternal near-miss if they occur during pregnancy, childbirth or within 42 days of pregnancy ending (including birth, abortion or miscarriage).

If one or more criteria are met and the woman survives the case will be counted as a near-miss event.

If the woman subsequently dies during the reporting period, then the case will be classified as a maternal death and not a near-miss event.

Medical events that are considered as near-miss:

Cardiac:

- Cardiac Arrest

(Sudden absence of pulse and loss of consciousness)

- Cardiopulmonary resuscitation

(A set of emergency procedures including chest compressions and lung ventilation applied in cardiac arrest victims)

Clotting:

- Failure to form clots

(The clinical inability to form clots/disseminated intravascular coagulation. Clinically, absence of clotting from the IV site or suture after 7–10 minutes.)

Respiratory:

- Gasping

(A terminal respiratory pattern. The breath is convulsively and audibly caught.)

- Cyanosis

(A bluish color of the skin and mucous membranes due to hypoxaemia (insufficient oxygen being carried in the blood).

- Need for invasive ventilation (not due to anesthesia)

(Requirement for invasive ventilation (mechanical ventilation in which positive pressure is applied to the patient's lungs via an artificial airway device), this does not include provision of oxygen or non-invasive ventilation alone)

Liver:

- Jaundice

(Clinically observed yellowing of the skin or sclera (whites of the eyes), raised bilirubin levels do not require laboratory confirmation)

Brain:

- Unconsciousness (not induced by anesthesia/sedation)

(Any loss of consciousness lasting more than 12 hours, involving complete or almost complete lack of responsiveness to external stimuli. A state compatible with Coma Glasgow Scale <10)

- Stroke

(Rapidly developing clinical signs of focal or global disturbance of cerebral function, lasting more than 24 hours)

- Paralysis

(The complete or partial paralysis of both sides of the body)

- Uncontrollable fit

(Refractory, persistent convulsions. Status epilepticus).

Surgery:

- Hysterectomy

(In the maternal near-miss context, surgical removal of the uterus following infection or hemorrhage)

- Emergency laparotomy

(Requirement for an emergency surgical incision into the abdominal cavity, other than for a primary procedure to carry out a caesarean section (irrespective of fetal viability) or for treatment of suspected or confirmed ectopic pregnancy)

Severe infection-related morbidity

Deep surgical site or deep perineal/labial/vaginal tear infection

The event must occur within 30 days after the operative procedure or birth related injury (where day 1 = the procedure or birth date)

AND involve **deep** soft tissues of the incision or tear (for example, fascial and muscle layers)

AND the patient has at least one of the following:

- purulent drainage from the deep incision or wound.
- a deep incision or tear that spontaneously opens, or is deliberately opened or aspirated by a surgeon, physician or clinician/midwife

AND patient has at least one of the following signs or symptoms:

- fever or localized pain or tenderness.
- an abscess or other evidence of infection involving the deep incision or tear that is detected on gross anatomical or histopathologic exam, or imaging test

Deep reproductive tract or body cavity infection-related to birth

The event must occur within 30 days after the operative procedure or birth (where day 1 = the procedure or birth date)

AND

involve any part of the body deeper than the fascial/muscle layers that is opened or manipulated during the operative procedure, or is suspected to have been injured as a consequence of the birth process

AND

The patient has at least ***one*** of the following:

a) purulent drainage from a drain or aspiration procedure, or through the vagina or abdominal incision from the organ/space.

OR

b) an abscess or other evidence of infection involving the organ/space (including the ovaries, fallopian tubes or uterus or abdominal cavity) that is detected on gross anatomical or histopathologic exam, or imaging test evidence suggestive of infection.

AND

Patient has at least ***two*** of the following signs or symptoms: fever or pain or tenderness (uterine or abdominal), or purulent vaginal discharge.

Figure S1: Randomization of Health Facilities in the Cluster-Randomized Trial

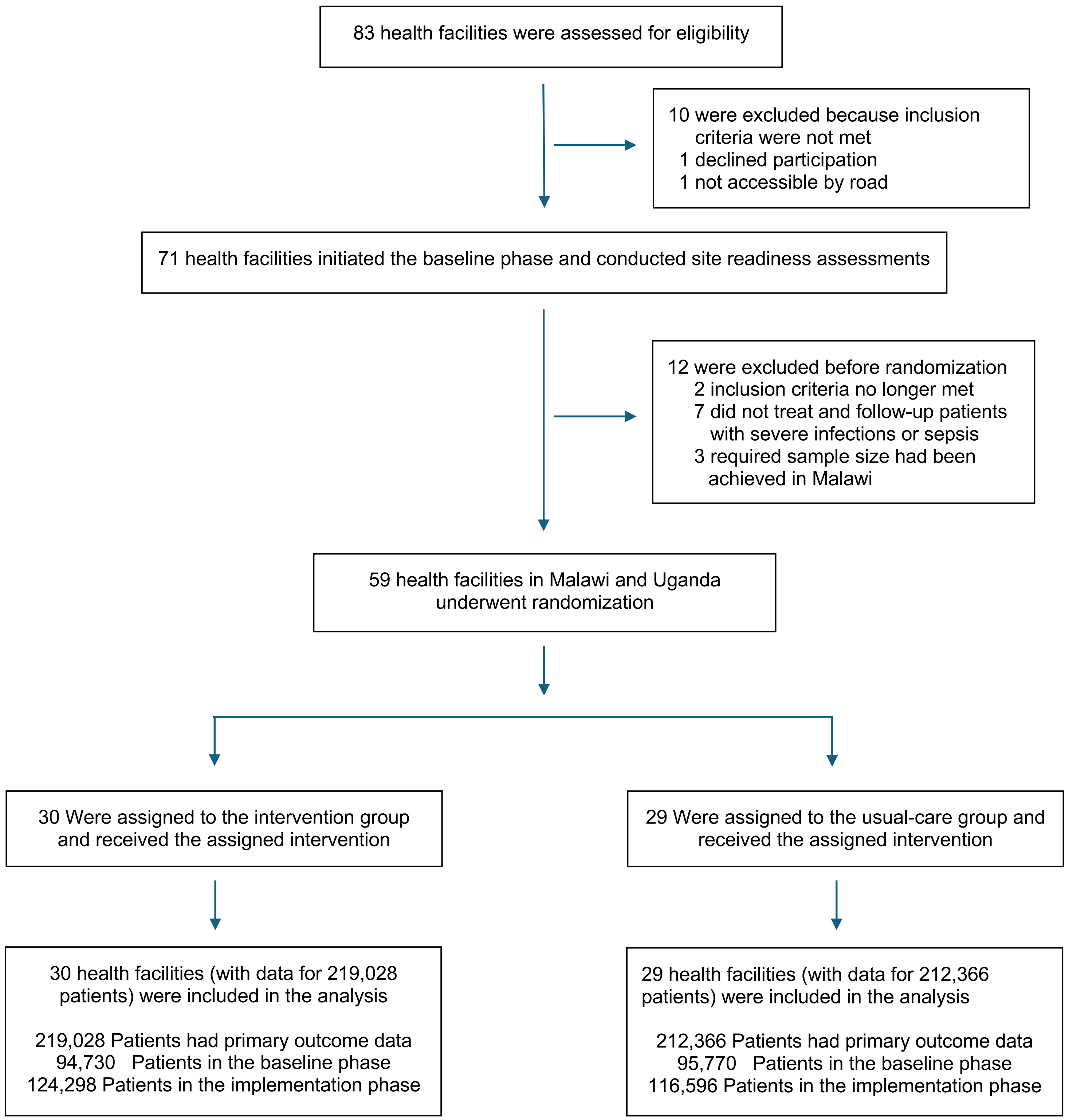

All the participating health facilities entered at least a 6-month baseline period in which they provided usual care for maternal infections and sepsis. In each country as facilities completed the baseline phase they were randomly assigned in a 1:1 ratio to provide the trial intervention (the APT-Sepsis program) or continue providing usual care with passive guideline dissemination for 12 months.
Hospitals were widely geographically distributed across both Malawi and Uganda. In Malawi, this included district hospitals, rural hospitals and facilities operated through the Christian Health Association of Malawi. In Uganda, the facilities included regional referral hospitals, general hospitals and level 4 health centers.

Table S1: Table on the representativeness of study participants

Category	Example
Disease, problem, or condition under investigation	Maternal infections and sepsis
Special considerations related to	
Sex and gender	Those at risk include women who are pregnant or have recently been pregnant.
Age	Both young age at pregnancy and older age (>35 years) are risk factors for maternal sepsis and infection related adverse outcomes.
Race or ethnic group	The risk of maternal sepsis and infection related mortality and morbidity is higher for black women.
Geography	Maternal deaths from sepsis are highest in Sub-Saharan Africa (estimated 70% of deaths) and Central and Southern Asia (17%).
Other considerations	Malawi and Uganda were chosen as they are high burden countries, with resource limited health systems. More than 99% of maternal sepsis deaths occur in low- and middle-income countries.
Overall representativeness of this trial	There was no individual patient consent, with outcomes obtained from all women receiving care after birth or pregnancy loss, irrespective of age or socio-economic status. Sites were widely distributed across Malawi and Uganda and represent a broad range of facility types. The participants are likely to be highly representative of the study population across Malawi and Uganda, and results generalizable to settings outside of these countries, especially other high burden countries which have facilities that operate in resource limited health systems.

Table S2: Primary and secondary outcomes including pre-randomization period and absolute risk differences.

Outcome	Pre-randomization		Post-randomization			
**Primary outcome**	**Intervention**	**Usual Care**	**Intervention**	**Usual Care**	**Risk Ratio (95% CI)***	**Risk difference (95% CI)***
Composite of infection-related maternal mortality, or infection-related near-miss, or deep surgical site/perineal infection or body cavity infection. (%)	2284/94730 (2.41)	2159/95770 (2.25)	1752/124298 (1.41)	2208/116596 (1.89)	0.68 (0.55 to 0.83)	-0.69 (-1.02 to -0.35)
**Components of the primary outcome§**						
Infection-related maternal mortality	79/94730 (0.08)	70/95770 (0.07)	90/124298 (0.07)	77/116596 (0.07)	0.96 (0.69, 1.32)	-0.003 (-.026, 0.20)
Infection-related near-miss	170/94730 (0.18)	188/95770 (0.20)	119/124298 (0.10)	141/116596 (0.12)	0.82 (0.54, 1.25)	-0.03 (-0.80, 0.26)
Severe infection-related morbidity	2184/94730 (2.31)	2037/95770 (2.13)	1672/124298 (1.35)	2102/116596 (1.80)	0.68 (0.55, 0.84)	-0.66 (-0.99, -0.33)
**Secondary outcomes**						
Stillbirth ¶	2267/96997 (2.34)	2054/97824 (2.10)	2708/127006 (2.13)	2314/118910 (1.95)	0.90 (0.73, 1.10)	-0.22 (-0.62,0.17)
Neonatal death ||	2334/94730 (2.46)	2456/95770 (2.56)	2691/124298 (2.16)	2761/116596 (2.37)	0.88 (0.73,1.04)	-0.34 (-0.79, 0.11)
Neonatal death (infection-related)	887/94730 (0.94)	622/95770 (0.65)	819/124298 (0.66)	622/116596 (0.53)	0.96 (0.74,1.24)	-0.01 (-0.66, 0.47)
Maternal mortality (any cause)	249/94730 (0.26)	222/95770 (0.23)	288/124298 (0.23)	235/116596 (0.20)	0.86 (0.57,1.30)	-0.11 (-0.40, 0.18)
Maternal near miss (any cause)	985/94730 (1.04)	772/95770 (0.81)	771/124298 (0.62)	609/116596 (0.52)	0.90 (0.67, 1.21)	-0.07 (-0.28, 0.13)
Maternal severe acute respiratory infection~	15/94730 (0.02)	10/95770 (0.01)	10/124298 (0.01)	7/116596 (0.01)	1.04 (0.45,2.39)	0.0003 (-0.01, 0.01)

*The width of the confidence intervals for secondary outcomes have not been adjusted for multiplicity and cannot be used to infer treatment effects.

§ An individual could be included in more than one component of the primary outcome, but would only have been counted once when the primary outcome was calculated

¶ A baby that died before of during birth after a gestational age of 28 weeks, with gestational age as determined by the facility medical team. The denominator includes livebirths and stillbirths.

||Death of a live-born infant within the first 28 completed days of life, only deaths occurring within the health facility were reported.

~ Defined as deaths or near miss events due to maternal severe acute respiratory infection

Table S3: Post-hoc sensitivity analyses of primary outcome

	Pre-randomization		Post-randomization			
	Intervention	Usual Care	Intervention	Usual Care	Risk Ratio (95% CI)*	Risk difference (95% CI)*
**Primary analysis**	2284/94730 (2.4)	2159/95770 (2.3)	1752/124298 (1.4)	2208/116596 (1.9)	0.68 (0.55 to 0.83)	-0.69 (-1.02 to -0.35)
**Sensitivity analysis including still birth and early pregnancy loss in denominator**	2284/106553 (2.1)	2159/107182 (2.0)	1752/138048 (1.3)	2208/129831 (1.7)	0.69 (0.56 to 0.84)	-0.60 (-0.89 to -0.31)
**Sensitivity analysis including deaths and near misses where it could not be determined whether they were infection-related**	2294/94730 (2.4)	2169/95770 (2.3)	1756/124298 (1.4)	2211/116596 (1.9)	0.68 (0.55 to 0.84)	-0.69 (-1.02 to -0.35)

*The width of the confidence intervals for secondary outcomes have not been adjusted for multiplicity and cannot be used to infer treatment effects.

Table S4: Prespecified Subgroup Analysis of the primary outcome, including pre-randomization phase

	Pre-randomization		Post-randomization		
**Subgroup**	**Intervention**	**Usual Care**	**Intervention**	**Usual Care**	**Risk Ratio***
	no. with event/total no. (%)		*no. with event/total no. (%)*		*(95% CI)*
Country					
Malawi	979/53230 (1.8)	1093/62252 (1.8)	626/63363 (1.0)	986/70615 (1.4)	0.68 (0.53 to 0.87)
Uganda	1305/41500 (3.1)	1066/33518 (3.2)	1126/60935 (1.8)	1222/45981 (2.7)	0.68 (0.52 to 0.90)
Facility Size					
Small§	468/15822 (3.0)	538/18235 (3.0)	335/22102 (1.5)	507/24034 (2.1)	0.63 (0.48 to 0.82)
Medium§	758/31111 (2.4)	604/25386 (2.4)	553/39284 (1.4)	644/30323 (2.1)	0.70 (0.52 to 0.94)
Large§	1058/47797 (2.2)	1017/52149 (1.9)	864/62912 (1.4)	1057/62239 (1.7)	0.71 (0.49 to 1.02)
Time post randomisation					
Pre-randomisation	2284/94730 (2.4)	2159/95770 (2.3)			
Month 1			254/12703 (2.0)	244/12117 (2.0)	0.92 (0.68 to 1.25)
Month 2			176/12295 (1.4)	230/12009 (1.9)	0.67 (0.51 to 0.88)
Month 3			174/12872 (1.4)	240/12136 (2.0)	0.63 (0.46 to 0.86)
Month 4			209/12363 (1.7)	227/11682 (1.9)	0.78 (0.57 to 1.06)
Month 5			150/12664 (1.2)	204/11696 (1.7)	0.60 (0.41 to 0.88)
Month 6			185/13026 (1.4)	220/12285 (1.8)	0.70 (0.49 to 1.00)
Month 7			170/12415 (1.4)	226/11478 (2.0)	0.64 (0.45 to 0.92)
Month 8			178/12646 (1.4)	225 /11842 (1.9)	0.68 (0.49 to 0.94)
Month 9			148/11848 (1.3)	218/11116 (2.0)	0.60 (0.44 to 0.82)
Month 10			108/11466 (0.9)	174 /10235 (1.7)	0.53 (0.35 to 0.80)

* The width of the confidence intervals for secondary outcomes have not been adjusted for multiplicity and cannot be used to infer treatment effects.

§ Facility size was defined by tertile at the point of randomization, within each country.

Table S5: Primary cause of severe infection

	Pre-randomization		Post-randomization	
	**Intervention (n=30 clusters)**	**Usual Care (n=29 clusters)**	**Intervention (n=30 clusters)**	**Usual Care (n=29 clusters)**
**Patients with severe infection related morbidity**	n=2175	n=2027	n=1660	n=2089
**Primary cause of severe infection**				
Deep surgical site infection	736 (33.8)	778 (38.4)	767 (46.2)	957 (45.8)
Deep perineal wound or episiotomy infection	235 (10.8)	188 (9.3)	126 (7.6)	169 (8.1)
Deep reproductive tract or body cavity infection	1204 (55.4)	1061 (52.3)	767 (46.2)	963 (46.1)

Table S6: Characteristics of patients pre- and post-randomization

	Pre-randomization		Post-randomization	
	Intervention	Usual Care	Intervention	Usual Care
Live births – no.	94730	95770	124298	116596
Early pregnancy loss – no.	9656	9358	11042	10921
Cesarean section births – no. (%)†	24,154 (25.5)	21,205 (22.1)	32,856 (26.4)	28,390 (24.3)
Vaginal births – no. (%)†	67,343 (71.1)	70,512 (73.6)	87,667 (70.5)	83,786 (71.9)
Born before arrival – no. (%)†	1259 (1.4)	1575 (1.6)	1480 (1.3)	1915 (1.3)
Postpartum hemorrhage (>1 Litre) – no. (%)†	1,680 (1.8)	1,355 (1.4)	2,062 (1.7)	1,761 (1.5)
Severe pre-eclampsia or eclampsia – no. (%)†	1,201 (1.3)	1,262 (1.3)	1,381 (1.1)	1,433 (1.2)
Forceps or vacuum births – no. (%)†	628 (0.7)	914 (1.0)	719 (0.6)	933 (0.8)
Vaginal breech delivery births – no. (%)†	1,346 (1.4)	1,564 (1.6)	1,576 (1.3)	1,572 (1.3)
Neonatal deaths – no.	2334 (2.5)	2456 (2.6)	2691 (2.2)	2761 (2.4)

†Percentages were calculated from the number of live births.

Table S7: Characteristics of women who died, had a near miss, or had a severe infection

	Pre-randomization		Post-randomization	
	Intervention (n=30 clusters)	Usual Care (n=29 clusters)	Intervention (n=30 clusters)	Usual Care (n=29 clusters)
**All patients (death, near-miss or severe infection)**				
**Age**				
N	3201	2822	2543	2799
Mean (SD)	25.1 (6.7)	25.2 (6.8)	25.4 (6.8)	24.9 (6.7)
Median (IQR)	24 (10)	24 (10)	24 (11)	24 (11)
Min, Max	13, 50	13, 48	14, 48	13, 47
Missing	2	0	0	0
**Pregnancy/post-delivery status on admission, N (%)**				
Pregnant <12 weeks gestation	108 (3.4%)	74 (2.6%)	52 (2.0%)	70 (2.5%)
Pregnant 12-28 weeks gestation	174 (5.4%)	135 (4.8%)	87 (3.4%)	133 (4.8%)
Pregnant 28+ weeks gestation	1187 (37.1%)	938 (33.2%)	914 (35.9%)	765 (27.3%)
Postnatal following livebirth or stillbirth (in the last 6 weeks)	1456 (45.5%)	1338 (47.4%)	1289 (50.7%)	1499 (53.6%)
Early pregnancy loss in the last 6 weeks	276 (8.6%)	337 (11.9%)	201 (7.9%)	332 (11.9 %)
Missing	2	0	0	0
**Patients with infection-related death, near-miss or severe infection**				
**Age**				
N	2282	2159	1752	2208
Mean (SD)	24.2 (6.4)	24.4 (6.5)	24.4 (6.7)	24.2 (6.4)
Median (IQR)	23 (9)	23 (10)	23 (10)	23 (9)
Min, Max	13, 48	13,48	14, 48	13, 47
Missing	2	0	0	0

	Pre-randomization		Post-randomization	
	Intervention (n=30 clusters)	Usual Care (n=29 clusters)	Intervention (n=30 clusters)	Usual Care (n=29 clusters)
**Pregnancy/post-delivery status on admission, N (%)**				
Pregnant <12 weeks gestation	78 (3.4%)	45 (2.1%)	39 (2.2%)	49 (2.2%)
Pregnant 12-28 weeks gestation	125 (5.5%)	86 (4.0%)	49 (2.8%)	95 (4.3%)
Pregnant 28+ weeks gestation	598 (26.2%)	533 (24.7%)	402 (23.0%)	428 (19.4%)
Postnatal following livebirth or stillbirth (in the last 6 weeks)	1226 (53.7%)	1178 (54.6%)	1073 (61.2%)	1318 (59.7%)
Early pregnancy loss in the last 6 weeks	255 (11.2%)	317 (14.7%)	189 (10.8%)	318 (14.4%)
Missing	2	0	0	0
**Has the patient had a surgical procedure during the current pregnancy or post-delivery period? N (%)**				
Yes	1116 (51.3%)	1201 (59.3%)	1044 (62.9%)	1235 (59.1%)
No	1059 (48.7%)	825 (40.7%)	617 (37.2%)	854 (40.9%)
Missing	109	133	91	119

Table S8 Estimates of intracluster correlation and cluster autocorrelation

Outcome	Intracluster correlation coefficient (95% CI)	Cluster autocorrelation
**Primary outcome**		
Composite primary outcome	0.00402 (0.00296, 0.00546)	0.39798
**Components of the composite**		
Infection-related maternal mortality	0.00041 (0.00025, 0.00069)	1.00000
Infection-related near-miss	0.00082 (0.00053, 0.00128)	Not estimated[1]
Severe infection-related morbidity	0.00415 (0.00305, 0.00563)	0.41324
**Other secondary clinical outcomes**		
Stillbirth	0.00324 (0.00228, 0.00460)	0.75734
Neonatal death	0.00802 (0.00563, 0.01141)	0.84500
Neonatal death (infection-related)	0.01715 (0.01212, 0.02422)	0.85325
Maternal mortality (any cause)	0.00080 (0.00052, 0.00122)	1.00000
Maternal near miss (any cause)	0.00523 (0.00370, 0.00740)	0.76980

Estimates of the intracluster correlation coefficient and cluster autocorrelation were estimated by fitting a mixed-effects linear model to the data with random effect for cluster and for a cluster-period interaction. CI: Confidence interval

[1] Estimation of correlations from full model did not converge, ICC was estimated from model removing cluster period interaction.

Table S9: Health facility operating theatre and sterilization characteristics

		Pre-randomization		Post randomization	
**Characteristic**	**Category/Measure**	**APT-SEPSIS**	**Usual Care**	**APT-SEPSIS**	**Usual Care**
Proportion of weeks labor ward theatres in operation	Mean (Sd)<br>Median (LQ, UQ)<br>(Minimum, Maximum)	13.2 (28.8)<br>0.0 (0.0, 8.0)<br>0.0 to 96.9	6.7 (16.4)<br>2.6 (0.0, 4.9)<br>0.0 to 71.1	14.3 (33.3)<br>0.0 (0.0, 2.4)<br>0.0 to 100	7.4 (20.8)<br>2.2 (0.0, 2.3)<br>0.0 to 93.3
Proportion of weeks with functioning autoclave within the facility	Mean (Sd)<br>Median (LQ, UQ)<br>(Minimum, Maximum)	96.0 (8.4)<br>100 (97.1, 100)<br>66.7 to 100	93.4 (17.3)<br>100 (96.0, 100)<br>9.8 to 100	92.0 (12.7)<br>95.2 (91.1, 100)<br>36.6 to 100	92.8 (12.9)<br>95.3 (93.0, 100)<br>34.1 to 100

Table S10: Health facility diagnostic equipment availability

Characteristic	Category/Measure	Pre-randomization		Post randomization	
		APT-SEPSIS	Usual Care	APT-SEPSIS	Usual Care
Proportion of weeks X-ray facilities available	**Available**<br>Mean (Sd)<br>Median (LQ, UQ)<br>(Minimum, Maximum)	60.0 (39.0)<br>72.2 (16.7, 95.1)<br>0.0 to 100	62.1 (36.5)<br>69.4 (41.7, 97.2)<br>0.0 to 100	56.6 (41.5)<br>62.8 (9.8, 97.8)<br>0.0 to 100	66.8 (36.8)<br>86.0 (42.2, 95.3)<br>0.0 to 100
	**Limited**<br>Mean (Sd)<br>Median (LQ, UQ)<br>(Minimum, Maximum)	18.4 (26.4)<br>4.5 (0.0, 33.3)<br>0.0 to 94.1	14.8 (23.4)<br>0.0 (0.0, 26.8)<br>0.0 to 75.0	14.6 (25.4)<br>2.3 (0.0, 17.8)<br>0.0 to 97.8	13.5 (20.6)<br>4.9 (0.0, 11.1)<br>0.0 to 84.4
	**None**<br>Mean (Sd)<br>Median (LQ, UQ)<br>(Minimum, Maximum)	21.7 (34.1)<br>5.9 (0.0, 16.7)<br>0.0 to 100	23.1 (33.8)<br>5.6 (0.0, 41.7)<br>0.0 to 100	28.9 (36.4)<br>6.7 (0.0, 53.5)<br>0.0 to 100	19.7 (31.0)<br>4.7 (0.0, 27.9)<br>0.0 to 97.8
Proportion of weeks ultrasound facilities available	**Available**<br>Mean (Sd)<br>Median (LQ, UQ)<br>(Minimum, Maximum)	78.9 (32.7)<br>97.2 (63.9, 100)<br>0.0 to 100	83.7 (24.8)<br>97.4 (79.4, 100)<br>0.0 to 100	81.3 (33.6)<br>97.7 (86.7, 100)<br>0.0 to 100	80.2 (32.1)<br>97.8 (81.4, 100)<br>2.4 to 100
	**Limited**<br>Mean (Sd)<br>Median (LQ, UQ)<br>(Minimum, Maximum)	10.3 (22.1)<br>0.0 (0.0, 5.9)<br>0.0 to 97.1	13.8 (20.0)<br>2.4 (0.0, 20.6)<br>0.0 to 64.0	11.3 (24.7)<br>0.0 (0.0, 6.7)<br>0.0 to 97.8	14.9 (25.7)<br>2.2 (0.0, 13.3)<br>0.0 to 95.3
	**None**<br>Mean (Sd)	10.8 (25.5)	2.5 (7.5)	7.4 (23.3)	4.9 (14.6)

Characteristic	Category/Measure	Pre-randomization		Post randomization	
		APT-SEPSIS	Usual Care	APT-SEPSIS	Usual Care
	Median (LQ, UQ)<br>(Minimum, Maximum)	0.0 (0.0, 5.9)<br>0.0 to 100	0.0 (0.0, 0.0)<br>0.0 to 36.0	0.0 (0.0, 2.3)<br>0.0 to 93.3	0.0 (0.0, 0.0)<br>0.0 to 60.0
Proportion of weeks full blood count testing available	**Available**<br>Mean (Sd)<br>Median (LQ, UQ)<br>(Minimum, Maximum)	 68.7 (29.8)<br>74.1 (55.9, 94.1)<br>0.0 to 100	 78.2 (19.0)<br>81.6 (61.1, 97.1)<br>43.9 to 100	 74.0 (30.2)<br>88.0 (58.1, 100)<br>2.2 to 100	 79.1 (24.1)<br>86.7 (70.7, 93.3)<br>0.0 to 100
	**Limited**<br>Mean (Sd)<br>Median (LQ, UQ)<br>(Minimum, Maximum)	 10.8 (14.5)<br>5.9 (0.0, 14.6)<br>0.0 to 55.9	 7.9 (11.8)<br>2.9 (0.0, 8.3)<br>0.0 to 44.7	 10.7 (21.6)<br>0.0 (0.0, 8.9)<br>0.0 to 97.8	 7.1 (12.0)<br>2.4 (0.0, 11.1)<br>0.0 to 62.2
	**None**<br>Mean (Sd)<br>Median (LQ, UQ)<br>(Minimum, Maximum)	 20.5 (26.0)<br>14.8 (0.0, 30.6)<br>0.0 to 100	 14.0 (14.9)<br>7.9 (2.6, 26.5)<br>0.0 to 48.8	 15.3 (24.2)<br>3.5 (0.0, 24.4)<br>0.0 to 87.8	 13.8 (22.8)<br>4.7 (2.2, 12.2)<br>0.0 to 100
Proportion of weeks renal function testing available	**Available**<br>Mean (Sd)<br>Median (LQ, UQ)<br>(Minimum, Maximum)	 49.8 (37.6)<br>50.1 (8.0, 91.7)<br>0.0 to 100	 62.5 (29.6)<br>66.7 (40.0, 87.5)<br>5.9 to 100	 50.8 (38.8)<br>47.9 (17.1, 93.3)<br>0.0 to 100	 62.2 (31.3)<br>67.4 (41.9, 88.9)<br>2.2 to 97.8
	**Limited**<br>Mean (Sd)<br>Median (LQ, UQ)<br>(Minimum, Maximum)	 18.9 (25.9)<br>8.3 (2.8, 26.5)<br>0.0 to 94.1	 14.6 (20.6)<br>5.6 (0.0, 21.9)<br>0.0 to 88.9	 17.5 (26.0)<br>4.6 (0.0, 30.2)<br>0.0 to 95.6	 12.3 (19.8)<br>4.7 (0.0, 11.1)<br>0.0 to 77.8

Characteristic	Category/Measure	Pre-randomization		Post randomization	
		APT-SEPSIS	Usual Care	APT-SEPSIS	Usual Care
	**None**<br>Mean (Sd)<br>Median (LQ, UQ)<br>(Minimum, Maximum)	31.3 (32.7)<br>16.5 (3.1, 58.3)<br>0.0 to 100	22.9 (22.9)<br>19.4 (2.8, 36.8)<br>0.0 to 78.0	31.7 (36.9)<br>12.2 (0.0, 69.8)<br>0.0 to 100	25.5 (26.4)<br>19.5 (2.4, 41.5)<br>0.0 to 97.8
Proportion of weeks blood culture testing available	**Available**<br>Mean (Sd)<br>Median (LQ, UQ)<br>(Minimum, Maximum)	28.1 (40.2)<br>4.7 (0.0, 55.9)<br>0.0 to 100	16.4 (27.0)<br>2.4 (0.0, 19.5)<br>0.0 to 100	30.2 (38.1)<br>8.3 (0.0, 48.8)<br>0.0 to 100	21.9 (31.4)<br>6.7 (2.2, 31.1)<br>0.0 to 97.8
	**Limited**<br>Mean (Sd)<br>Median (LQ, UQ)<br>(Minimum, Maximum)	10.7 (14.0)<br>4.3 (0.0, 14.7)<br>0.0 to 46.9	10.7 (12.6)<br>7.3 (2.4, 12.2)<br>0.0 to 42.1	8.7 (16.0)<br>2.2 (0.0, 9.8)<br>0.0 to 69.8	6.6 (13.8)<br>2.2 (0.0, 7.3)<br>0.0 to 64.4
	**None**<br>Mean (Sd)<br>Median (LQ, UQ)<br>(Minimum, Maximum)	61.2 (38.8)<br>67.1 (11.1, 97.2)<br>0.0 to 100	72.9 (29.9)<br>87.8 (61.8, 94.4)<br>0.0 to 100	61.1 (38.9)<br>78.6 (30.2, 97.6)<br>0.0 to 100	71.5 (34.8)<br>90.2 (46.3, 97.6)<br>0.0 to 100
Proportion of weeks wound swab testing available	**Available**<br>Mean (Sd)<br>Median (LQ, UQ)<br>(Minimum, Maximum)	33.6 (41.6)<br>1.5 (0.0, 87.5)<br>0.0 to 100	25.1 (30.3)<br>11.8 (0.0, 41.5)<br>0.0 to 95.1	35.2 (39.7)<br>5.8 (0.0, 63.4)<br>0.0 to 100	30.0 (38.0)<br>6.7 (0.0, 55.6)<br>0.0 to 100
	**Limited**<br>Mean (Sd)<br>Median (LQ, UQ)<br>(Minimum, Maximum)	7.8 (9.2)<br>4.3 (0.0, 12.5)<br>0.0 to 31.7	13.2 (13.0)<br>8.3 (2.7, 24.0)<br>0.0 to 44.7	6.9 (10.8)<br>1.1 (0.0, 14.0)<br>0.0 to 35.6	7.6 (14.7)<br>2.3 (0.0, 8.9)<br>0.0 to 73.3

Characteristic	Category/Measure	Pre-randomization		Post randomization	
		APT-SEPSIS	Usual Care	APT-SEPSIS	Usual Care
	**None**<br>Mean (Sd)<br>Median (LQ, UQ)<br>(Minimum, Maximum)	58.7 (41.4)<br>73.5 (8.3, 97.6)<br>0.0 to 100	61.7 (30.9)<br>68.0 (51.2, 90.2)<br>0.0 to 100	57.9 (41.7)<br>69.8 (15.6, 97.8)<br>0.0 to 100	62.4 (38.8)<br>76.7 (34.9, 97.7)<br>0.0 to 100
Proportion of weeks urine microscopy available	**Available**<br>Mean (Sd)<br>Median (LQ, UQ)<br>(Minimum, Maximum)	89.6 (24.8)<br>100 (93.8, 100)<br>2.9 to 100	93.4 (10.4)<br>97.2 (92.0, 100)<br>60.5 to 100	90.2 (25.3)<br>100 (97.6, 100)<br>2.3 to 100	93.5 (15.8)<br>100 (92.7, 100)<br>20.0 to 100
	**Limited**<br>Mean (Sd)<br>Median (LQ, UQ)<br>(Minimum, Maximum)	5.9 (17.8)<br>0.0 (0.0, 2.8)<br>0.0 to 93.8	5.1 (10.2)<br>0.0 (0.0, 5.9)<br>0.0 to 39.5	4.9 (17.0)<br>0.0 (0.0, 2.3)<br>0.0 to 90.7	5.3 (14.4)<br>0.0 (0.0, 2.3)<br>0.0 to 71.1
	**None**<br>Mean (Sd)<br>Median (LQ, UQ)<br>(Minimum, Maximum)	4.5 (13.4)<br>0.0 (0.0, 3.1)<br>0.0 to 70.6	1.5 (2.4)<br>0.0 (0.0, 2.8)<br>0.0 to 8.0	4.9 (14.3)<br>0.0 (0.0, 2.2)<br>0.0 to 60.5	1.2 (2.4)<br>0.0 (0.0, 2.2)<br>0.0 to 8.9
Proportion of weeks pregnancy tests available	**Available**<br>Mean (Sd)<br>Median (LQ, UQ)<br>(Minimum, Maximum)	83.7 (23.0)<br>93.4 (80.6, 100)<br>17.6 to 100	82.2 (24.1)<br>97.1 (69.4, 100)<br>16.7 to 100	92.3 (15.1)<br>100 (91.1, 100)<br>42.2 to 100	94.4 (14.6)<br>100 (97.8, 100)<br>28.9 to 100
	**Limited**<br>Mean (Sd)<br>Median (LQ, UQ)	5.0 (8.4)	3.8 (8.9)	2.8 (5.9)	4.7 (13.0)

Characteristic	Category/Measure	Pre-randomization		Post randomization	
		APT-SEPSIS	Usual Care	APT-SEPSIS	Usual Care
	(Minimum, Maximum)	0.0 (0.0, 7.3)<br>0.0 to 31.3	0.0 (0.0, 2.8)<br>0.0 to 36.8	0.0 (0.0, 2.4)<br>0.0 to 25.6	0.0 (0.0, 0.0)<br>0.0 to 62.2
	**None**<br>Mean (Sd)<br>Median (LQ, UQ)<br>(Minimum, Maximum)	 11.2 (17.1)<br>2.9 (0.0, 17.6)<br>0.0 to 61.0	 14.0 (21.4)<br>0.0 (0.0, 24.4)<br>0.0 to 80.6	 4.8 (11.0)<br>0.0 (0.0, 4.9)<br>0.0 to 48.9	 0.9 (2.2)<br>0.0 (0.0, 0.0)<br>0.0 to 8.9

Table S11: Health facility human resource availability

Characteristic	Category/Measure	Pre-randomization		Post randomization	
		APT-SEPSIS	Usual Care	APT-SEPSIS	Usual Care
Mean total beds from weekly report (all areas)	Mean (Sd)<br>Median (LQ, UQ)<br>(Minimum, Maximum)	96.9 (46.1)<br>90.9 (57.4, 125.0)<br>33.7 to 226.2	101.6 (39.8)<br>95.7 (69.2, 136.0)<br>34.4 to 178.3	98.2 (45.1)<br>90.8 (62.5, 126.6)<br>38.8 to 225.8	100.1 (41.6)<br>93.3 (66.7, 136.1)<br>30.1 to 181.7
Mean number of patients managed across all areas, from weekly report	Mean (Sd)<br>Median (LQ, UQ)<br>(Minimum, Maximum)	68.9 (37.1)<br>67.7 (32.8, 91.7)<br>20.6 to 168.2	64.3 (25.7)<br>59.6 (46.1, 74.8)<br>26.9 to 123.0	74.5 (42.4)<br>66.7 (35.2, 99.0)<br>20.0 to 174.9	66.6 (30.8)<br>61.3 (43.2, 83.4)<br>15.0 to 154.3
Mean number of nurses/midwives (not including students) working across all areas, from weekly report	Mean (Sd)<br>Median (LQ, UQ)<br>(Minimum, Maximum)	9.9 (5.3)<br>8.5 (5.8, 12.9)<br>3.4 to 29.4	10.0 (4.6)<br>9.7 (6.2, 12.3)<br>3.6 to 22.0	11.1 (5.9)<br>9.2 (6.5, 13.4)<br>4.3 to 25.3	10.8 (5.6)<br>10.9 (6.0, 14.5)<br>3.2 to 24.7

Table S12: Equipment availability over all areas in health facility

		Pre-randomization		Post randomization	
**Characteristic**	**Category/Measure**	**APT-SEPSIS**	**Usual Care**	**APT-SEPSIS**	**Usual Care**
Proportion of weeks equipment for IV-line insertion available	**Available (all areas)**<br>Mean (Sd)<br>Median (LQ, UQ)<br>(Minimum, Maximum)	73.3 (34.5)<br>89.6 (64.0, 100)<br>0.0 to 100	67.3 (37.0)<br>78.1 (48.8, 100)<br>0.0 to 100	80.8 (22.4)<br>87.8 (65.9, 100)<br>24.4 to 100	73.4 (35.8)<br>93.3 (46.3, 100)<br>2.2 to 100
	**Limited**<br>Mean (Sd)<br>Median (LQ, UQ)<br>(Minimum, Maximum)	23.4 (31.5)<br>10.4 (0.0, 30.6)<br>0.0 to 97.1	30.6 (34.3)<br>20.0 (0.0, 51.2)<br>0.0 to 97.2	17.2 (20.8)<br>10.4 (0.0, 30.2)<br>0.0 to 75.6	25.9 (35.1)<br>6.7 (0.0, 53.7)<br>0.0 to 95.6
	**None**<br>Mean (Sd)<br>Median (LQ, UQ)<br>(Minimum, Maximum)	3.2 (8.8)<br>0.0 (0.0, 0.0)<br>0.0 to 40.0	2.1 (4.8)<br>0.0 (0.0, 0.0)<br>0.0 to 16.0	2.0 (5.5)<br>0.0 (0.0, 0.0)<br>0.0 to 24.4	0.7 (2.1)<br>0.0 (0.0, 0.0)<br>0.0 to 9.8
Proportion of weeks working thermometers available	**Available (all areas)**<br>Mean (Sd)<br>Median (LQ, UQ)<br>(Minimum, Maximum)	45.9 (35.8)<br>44.4 (4.0, 80.5)<br>0.0 to 100	36.0 (34.9)<br>19.5 (5.6, 71.9)<br>0.0 to 100	78.4 (25.1)<br>88.6 (67.4, 97.6)<br>2.3 to 100	47.1 (34.5)<br>44.4 (15.6, 75.6)<br>0.0 to 100
	**Limited**<br>Mean (Sd)<br>Median (LQ, UQ)<br>(Minimum, Maximum)	53.9 (35.5)<br>55.6 (19.5, 92.0)<br>0.0 to 100	62.6 (34.0)<br>78.1 (28.1, 91.2)<br>0.0 to 100	21.6 (25.1)<br>11.4 (2.4, 32.6)<br>0.0 to 97.7	52.5 (34.4)<br>55.6 (22.0, 82.2)<br>0.0 to 100
	**None**<br>Mean (Sd)	0.2 (0.8)	1.4 (4.7)	0.0 (0.0)	0.4 (1.1)

		Pre-randomization		Post randomization	
**Characteristic**	**Category/Measure**	**APT-SEPSIS**	**Usual Care**	**APT-SEPSIS**	**Usual Care**
	Median (LQ, UQ)<br>(Minimum, Maximum)	0.0 (0.0, 0.0)<br>0.0 to 4.0	0.0 (0.0, 0.0)<br>0.0 to 19.4	0.0 (0.0, 0.0)<br>0.0 to 0.0	0.0 (0.0, 0.0)<br>0.0 to 4.9
Proportion of weeks working Blood Pressure device available	**Available (all areas)**<br>Mean (Sd)<br>Median (LQ, UQ)<br>(Minimum, Maximum)	15.8 (24.2)<br>6.8 (0.0, 16.7)<br>0.0 to 87.5	13.8 (22.1)<br>2.6 (0.0, 19.5)<br>0.0 to 100	29.9 (32.6)<br>14.8 (4.7, 41.9)<br>0.0 to 97.8	14.2 (22.7)<br>4.4 (2.2, 13.3)<br>0.0 to 97.6
	**Limited**<br>Mean (Sd)<br>Median (LQ, UQ)<br>(Minimum, Maximum)	84.2 (24.2)<br>93.2 (83.3, 100)<br>12.5 to 100	86.0 (22.0)<br>96.0 (80.5, 100)<br>0.0 to 100	70.1 (32.6)<br>85.2 (58.1, 95.3)<br>2.2 to 100	85.8 (22.7)<br>95.6 (86.7, 97.8)<br>2.4 to 100
	**None**<br>Mean (Sd)<br>Median (LQ, UQ)<br>(Minimum, Maximum)	0.0 (0.0)<br>0.0 (0.0, 0.0)<br>0.0 to 0.0	0.1 (0.7)<br>0.0 (0.0, 0.0)<br>0.0 to 4.0	0.0 (0.0)<br>0.0 (0.0, 0.0)<br>0.0 to 0.0	0.0 (0.0)<br>0.0 (0.0, 0.0)<br>0.0 to 0.0
Proportion of weeks working pulse oximetry machines available	**Available (all areas)**<br>Mean (Sd)<br>Median (LQ, UQ)<br>(Minimum, Maximum)	37.4 (29.8)<br>43.9 (5.6, 58.8)<br>0.0 to 100	30.9 (33.7)<br>13.2 (2.6, 58.8)<br>0.0 to 96.0	64.8 (35.0)<br>75.0 (37.2, 93.3)<br>0.0 to 100	39.7 (36.8)<br>31.1 (7.0, 72.1)<br>0.0 to 100
	**Limited**<br>Mean (Sd)<br>Median (LQ, UQ)<br>(Minimum, Maximum)	62.4 (29.5)<br>56.1 (41.2, 94.4)<br>0.0 to 100	68.3 (33.5)<br>82.4 (41.2, 97.4)<br>4.0 to 100	35.2 (35.0)<br>25.0 (6.7, 62.8)<br>0.0 to 100	60.1 (36.9)<br>68.9 (27.9, 93.0)<br>0.0 to 100

		Pre-randomization		Post randomization	
**Characteristic**	**Category/Measure**	**APT-SEPSIS**	**Usual Care**	**APT-SEPSIS**	**Usual Care**
	**None**<br>Mean (Sd)<br>Median (LQ, UQ)<br>(Minimum, Maximum)	0.2 (1.0)<br>0.0 (0.0, 0.0)<br>0.0 to 5.6	0.8 (4.5)<br>0.0 (0.0, 0.0)<br>0.0 to 24.0	0.0 (0.0)<br>0.0 (0.0, 0.0)<br>0.0 to 0.0	0.3 (1.4)<br>0.0 (0.0, 0.0)<br>0.0 to 7.3
Proportion of weeks fetoscopes / pinards / fetal stethoscopes available	**Available (all areas)**<br>Mean (Sd)<br>Median (LQ, UQ)<br>(Minimum, Maximum)	12.9 (25.5)<br>0.0 (0.0, 13.9)<br>0.0 to 93.8	8.4 (15.5)<br>0.0 (0.0, 8.3)<br>0.0 to 52.0	29.6 (33.1)<br>15.1 (2.2, 53.3)<br>0.0 to 97.6	11.4 (21.6)<br>0.0 (0.0, 4.9)<br>0.0 to 82.2
	**Limited**<br>Mean (Sd)<br>Median (LQ, UQ)<br>(Minimum, Maximum)	87.0 (25.5)<br>100 (86.1, 100)<br>6.3 to 100	91.0 (15.3)<br>100 (91.7, 100)<br>48.0 to 100	70.3 (33.2)<br>84.1 (46.7, 97.8)<br>2.4 to 100	87.6 (21.5)<br>97.8 (82.9, 100)<br>17.8 to 100
	**None**<br>Mean (Sd)<br>Median (LQ, UQ)<br>(Minimum, Maximum)	0.1 (0.5)<br>0.0 (0.0, 0.0)<br>0.0 to 2.8	0.6 (1.8)<br>0.0 (0.0, 0.0)<br>0.0 to 8.0	0.2 (0.6)<br>0.0 (0.0, 0.0)<br>0.0 to 2.4	0.9 (3.2)<br>0.0 (0.0, 0.0)<br>0.0 to 17.1
Proportion of weeks clock watches available	**Available (all areas)**<br>Mean (Sd)<br>Median (LQ, UQ)<br>(Minimum, Maximum)	43.8 (40.8)<br>33.2 (0.0, 85.4)<br>0.0 to 100	25.0 (34.5)<br>2.8 (0.0, 48.0)<br>0.0 to 100	56.2 (35.6)<br>54.8 (35.6, 95.3)<br>0.0 to 100	26.1 (34.4)<br>11.1 (2.2, 34.9)<br>0.0 to 100
	**Limited**<br>Mean (Sd)<br>Median (LQ, UQ)<br>(Minimum, Maximum)	54.2 (39.1)	70.1 (35.3)	42.2 (34.3)	72.7 (34.1)

		Pre-randomization		Post randomization	
**Characteristic**	**Category/Measure**	**APT-SEPSIS**	**Usual Care**	**APT-SEPSIS**	**Usual Care**
		65.4 (14.6, 97.1)<br>0.0 to 100	91.7 (38.2, 100)<br>0.0 to 100	40.7 (4.7, 62.8)<br>0.0 to 100	87.8 (65.1, 97.8)<br>0.0 to 100
	**None**<br>Mean (Sd)<br>Median (LQ, UQ)<br>(Minimum, Maximum)	2.0 (6.1)<br>0.0 (0.0, 0.0)<br>0.0 to 27.8	4.9 (15.8)<br>0.0 (0.0, 0.0)<br>0.0 to 75.0	1.6 (5.6)<br>0.0 (0.0, 0.0)<br>0.0 to 24.4	1.2 (6.5)<br>0.0 (0.0, 0.0)<br>0.0 to 34.9
Proportion of weeks spare batteries available	**Available (all areas)**<br>Mean (Sd)<br>Median (LQ, UQ)<br>(Minimum, Maximum)	13.4 (25.7)<br>0.0 (0.0, 12.5)<br>0.0 to 100	9.5 (17.1)<br>0.0 (0.0, 11.8)<br>0.0 to 60.0	44.9 (33.4)<br>44.1 (6.7, 70.7)<br>0.0 to 97.8	4.4 (10.3)<br>0.0 (0.0, 2.3)<br>0.0 to 48.8
	**Limited**<br>Mean (Sd)<br>Median (LQ, UQ)<br>(Minimum, Maximum)	50.4 (29.6)<br>50.0 (35.3, 72.0)<br>0.0 to 100	42.5 (31.8)<br>39.0 (11.1, 75.0)<br>0.0 to 100	32.9 (21.8)<br>39.3 (12.2, 46.7)<br>2.2 to 67.4	42.2 (30.3)<br>41.5 (15.6, 64.4)<br>0.0 to 100
	**None**<br>Mean (Sd)<br>Median (LQ, UQ)<br>(Minimum, Maximum)	36.1 (32.8)<br>29.3 (3.1, 52.9)<br>0.0 to 100	48.0 (37.5)<br>37.5 (12.0, 88.9)<br>0.0 to 100	22.2 (30.8)<br>3.4 (0.0, 35.6)<br>0.0 to 97.7	53.4 (32.0)<br>53.5 (33.3, 79.1)<br>0.0 to 97.7
Proportion of weeks urine dipsticks available	**Available (all areas)**<br>Mean (Sd)<br>Median (LQ, UQ)<br>(Minimum, Maximum)	1.4 (7.1)<br>0.0 (0.0, 0.0)<br>0.0 to 38.9	3.3 (10.1)<br>0.0 (0.0, 0.0)<br>0.0 to 52.0	3.7 (10.9)<br>0.0 (0.0, 2.2)<br>0.0 to 44.4	0.4 (1.3)<br>0.0 (0.0, 0.0)<br>0.0 to 4.9

		Pre-randomization		Post randomization	
**Characteristic**	**Category/Measure**	**APT-SEPSIS**	**Usual Care**	**APT-SEPSIS**	**Usual Care**
	**Limited**<br>Mean (Sd)<br>Median (LQ, UQ)<br>(Minimum, Maximum)	68.4 (34.8)<br>83.5 (52.0, 97.2)<br>4.0 to 100	66.1 (35.5)<br>81.3 (44.0, 94.4)<br>0.0 to 100	62.9 (35.5)<br>72.7 (32.6, 97.6)<br>4.4 to 100	63.5 (36.8)<br>76.7 (27.9, 97.7)<br>2.2 to 100
	**None**<br>Mean (Sd)<br>Median (LQ, UQ)<br>(Minimum, Maximum)	30.2 (35.3)<br>9.7 (0.0, 48.0)<br>0.0 to 96.0	30.6 (33.7)<br>14.6 (2.8, 44.0)<br>0.0 to 100	33.4 (36.4)<br>16.3 (2.2, 67.4)<br>0.0 to 95.6	36.1 (36.8)<br>23.3 (2.3, 72.1)<br>0.0 to 97.8
Proportion of weeks malaria rapid tests available	**Available (all areas)**<br>Mean (Sd)<br>Median (LQ, UQ)<br>(Minimum, Maximum)	2.5 (8.5)<br>0.0 (0.0, 0.0)<br>0.0 to 41.7	3.0 (9.9)<br>0.0 (0.0, 0.0)<br>0.0 to 48.0	4.0 (12.4)<br>0.0 (0.0, 0.0)<br>0.0 to 60.5	0.6 (2.0)<br>0.0 (0.0, 0.0)<br>0.0 to 8.9
	**Limited**<br>Mean (Sd)<br>Median (LQ, UQ)<br>(Minimum, Maximum)	42.5 (35.6)<br>41.0 (6.3, 81.3)<br>0.0 to 100	36.0 (37.1)<br>23.7 (3.1, 73.7)<br>0.0 to 100	39.7 (34.7)<br>25.6 (7.0, 62.2)<br>0.0 to 100	28.9 (34.1)<br>12.2 (2.4, 51.2)<br>0.0 to 97.7
	**None**<br>Mean (Sd)<br>Median (LQ, UQ)<br>(Minimum, Maximum)	55.0 (37.5)<br>57.2 (12.5, 93.8)<br>0.0 to 100	61.0 (38.2)<br>75.0 (26.3, 96.9)<br>0.0 to 100	56.3 (37.3)<br>65.1 (16.3, 93.0)<br>0.0 to 100	70.4 (35.3)<br>87.8 (48.8, 97.6)<br>0.0 to 100
Proportion of weeks working oxygen concentrators available	**Available (all areas)**<br>Mean (Sd)<br>Median (LQ, UQ)<br>(Minimum, Maximum)	47.9 (35.7)	45.1 (38.2)	58.7 (37.0)	48.2 (37.5)

		Pre-randomization		Post randomization	
**Characteristic**	**Category/Measure**	**APT-SEPSIS**	**Usual Care**	**APT-SEPSIS**	**Usual Care**
		54.6 (7.3, 83.3)<br>0.0 to 100	44.4 (2.6, 84.4)<br>0.0 to 97.6	71.7 (20.0, 92.7)<br>0.0 to 100	51.2 (11.1, 86.0)<br>0.0 to 100
	**Limited**<br>Mean (Sd)<br>Median (LQ, UQ)<br>(Minimum, Maximum)	51.6 (35.1)<br>45.4 (16.7, 91.2)<br>0.0 to 100	53.9 (37.4)<br>55.6 (15.6, 97.2)<br>2.4 to 100	41.2 (36.9)<br>28.3 (7.3, 80.0)<br>0.0 to 100	49.6 (36.3)<br>44.4 (14.0, 85.4)<br>0.0 to 100
	**None**<br>Mean (Sd)<br>Median (LQ, UQ)<br>(Minimum, Maximum)	0.5 (2.1)<br>0.0 (0.0, 0.0)<br>0.0 to 11.1	1.1 (5.2)<br>0.0 (0.0, 0.0)<br>0.0 to 28.0	0.2 (0.6)<br>0.0 (0.0, 0.0)<br>0.0 to 2.4	2.2 (10.5)<br>0.0 (0.0, 0.0)<br>0.0 to 56.1
Proportion of weeks bottled or piped oxygen available	**Available (all areas)**<br>Mean (Sd)<br>Median (LQ, UQ)<br>(Minimum, Maximum)	26.4 (32.6)<br>9.9 (0.0, 46.9)<br>0.0 to 100	15.2 (26.6)<br>0.0 (0.0, 25.0)<br>0.0 to 92.0	29.9 (38.4)<br>6.8 (0.0, 74.4)<br>0.0 to 100	14.8 (28.4)<br>0.0 (0.0, 18.6)<br>0.0 to 100
	**Limited**<br>Mean (Sd)<br>Median (LQ, UQ)<br>(Minimum, Maximum)	52.7 (32.2)<br>54.7 (30.6, 76.0)<br>0.0 to 100	52.1 (34.4)<br>46.9 (23.5, 87.8)<br>2.8 to 100	46.8 (35.7)<br>43.3 (13.3, 82.9)<br>0.0 to 100	56.8 (36.4)<br>65.1 (17.8, 93.0)<br>0.0 to 100
	**None**<br>Mean (Sd)<br>Median (LQ, UQ)<br>(Minimum, Maximum)	21.0 (28.8)<br>4.3 (0.0, 33.3)<br>0.0 to 100	32.6 (35.1)<br>12.2 (0.0, 71.9)<br>0.0 to 96.9	23.3 (31.1)<br>3.4 (0.0, 46.7)<br>0.0 to 97.6	28.3 (36.6)<br>2.3 (0.0, 57.8)<br>0.0 to 100

		Pre-randomization		Post randomization	
**Characteristic**	**Category/Measure**	**APT-SEPSIS**	**Usual Care**	**APT-SEPSIS**	**Usual Care**
Proportion of weeks gloves available	**Available (all areas)**<br>Mean (Sd)<br>Median (LQ, UQ)<br>(Minimum, Maximum)	71.6 (30.2)<br>85.1 (48.0, 97.2)<br>0.0 to 100	60.7 (39.4)<br>82.9 (18.4, 96.9)<br>0.0 to 100	82.3 (20.4)<br>92.9 (71.1, 97.7)<br>26.7 to 100	66.1 (37.3)<br>90.7 (46.3, 97.6)<br>0.0 to 100
	**Limited**<br>Mean (Sd)<br>Median (LQ, UQ)<br>(Minimum, Maximum)	24.2 (25.7)<br>14.9 (2.8, 44.0)<br>0.0 to 79.4	33.1 (33.4)<br>17.1 (3.1, 64.0)<br>0.0 to 97.2	15.9 (18.9)<br>7.1 (2.3, 26.7)<br>0.0 to 71.1	32.2 (36.0)<br>9.3 (2.4, 53.7)<br>0.0 to 97.8
	**None**<br>Mean (Sd)<br>Median (LQ, UQ)<br>(Minimum, Maximum)	4.2 (9.3)<br>0.0 (0.0, 2.9)<br>0.0 to 44.0	6.2 (11.7)<br>0.0 (0.0, 5.3)<br>0.0 to 41.7	1.7 (4.8)<br>0.0 (0.0, 0.0)<br>0.0 to 22.0	1.6 (3.9)<br>0.0 (0.0, 0.0)<br>0.0 to 15.6

Table S13: Water and handwashing availability over all areas in facility

		Pre-randomization		Post randomization	
**Characteristic**	**Category/Measure**	**APT-SEPSIS**	**Usual Care**	**APT-SEPSIS**	**Usual Care**
Proportion of weeks soap available	**Available**<br>Mean (Sd)<br>Median (LQ, UQ)<br>(Minimum, Maximum)	65.9 (36.0)<br>80.9 (43.8, 96.9)<br>0.0 to 100	62.4 (34.1)<br>71.9 (38.2, 94.1)<br>0.0 to 100	84.0 (24.4)<br>97.6 (71.1, 97.8)<br>2.2 to 100	61.7 (36.3)<br>73.3 (31.1, 95.1)<br>0.0 to 100
	**Limited**<br>Mean (Sd)<br>Median (LQ, UQ)<br>(Minimum, Maximum)	34.1 (36.0)<br>19.1 (3.1, 56.3)<br>0.0 to 100	36.2 (33.3)<br>26.5 (5.9, 58.3)<br>0.0 to 100	15.8 (24.2)<br>2.4 (2.2, 28.9)<br>0.0 to 97.8	38.1 (36.1)<br>26.7 (4.9, 68.9)<br>0.0 to 100
	**None**<br>Mean (Sd)<br>Median (LQ, UQ)<br>(Minimum, Maximum)	0.0 (0.0)<br>0.0 (0.0, 0.0)<br>0.0 to 0.0	1.4 (3.7)<br>0.0 (0.0, 0.0)<br>0.0 to 14.6	0.1 (0.8)<br>0.0 (0.0, 0.0)<br>0.0 to 4.4	0.2 (0.6)<br>0.0 (0.0, 0.0)<br>0.0 to 2.3
Proportion of weeks running/flowing water for handwashing available	**Available (all areas)**<br>Mean (Sd)<br>Median (LQ, UQ)<br>(Minimum, Maximum)	62.5 (41.5)<br>83.7 (8.8, 97.1)<br>0.0 to 100	65.6 (37.6)<br>85.4 (35.3, 96.9)<br>0.0 to 100	76.3 (32.4)<br>93.2 (63.4, 97.6)<br>0.0 to 100	66.2 (34.0)<br>79.1 (34.1, 95.3)<br>0.0 to 100
	**Limited**<br>Mean (Sd)<br>Median (LQ, UQ)<br>(Minimum, Maximum)	32.1 (37.4)<br>12.5 (2.9, 67.6)<br>0.0 to 100	26.6 (31.2)<br>12.2 (3.1, 53.7)<br>0.0 to 100	20.4 (28.9)<br>5.7 (2.4, 28.9)<br>0.0 to 100	28.1 (29.6)<br>18.6 (4.7, 42.2)<br>0.0 to 100
	**None**<br>Mean (Sd)	5.4 (17.6)	7.8 (24.0)	3.3 (14.3)	5.7 (15.5)

		Pre-randomization		Post randomization	
**Characteristic**	**Category/Measure**	**APT-SEPSIS**	**Usual Care**	**APT-SEPSIS**	**Usual Care**
	Median (LQ, UQ)<br>(Minimum, Maximum)	0.0 (0.0, 0.0)<br>0.0 to 76.0	0.0 (0.0, 0.0)<br>0.0 to 92.0	0.0 (0.0, 0.0)<br>0.0 to 78.0	0.0 (0.0, 0.0)<br>0.0 to 62.2
Proportion of weeks hand drying facilities available	**Available (all areas)**<br>Mean (Sd)<br>Median (LQ, UQ)<br>(Minimum, Maximum)	15.6 (28.7)<br>0.0 (0.0, 28.1)<br>0.0 to 100	19.7 (30.3)<br>0.0 (0.0, 27.8)<br>0.0 to 100	19.5 (31.6)<br>0.0 (0.0, 32.6)<br>0.0 to 100	14.3 (28.9)<br>0.0 (0.0, 7.0)<br>0.0 to 100
	**Limited**<br>Mean (Sd)<br>Median (LQ, UQ)<br>(Minimum, Maximum)	48.4 (31.2)<br>45.4 (20.6, 86.1)<br>0.0 to 93.8	42.9 (30.7)<br>44.4 (19.4, 72.0)<br>0.0 to 100	45.9 (32.6)<br>47.7 (13.3, 71.1)<br>0.0 to 100	46.1 (31.5)<br>44.2 (18.6, 69.8)<br>0.0 to 100
	**None**<br>Mean (Sd)<br>Median (LQ, UQ)<br>(Minimum, Maximum)	36.1 (32.4)<br>28.6 (8.3, 72.0)<br>0.0 to 86.1	37.4 (35.8)<br>28.0 (2.4, 67.6)<br>0.0 to 100	34.6 (36.7)<br>16.2 (0.0, 71.1)<br>0.0 to 97.6	39.6 (33.8)<br>37.8 (11.6, 67.4)<br>0.0 to 95.6
Proportion of weeks alcohol rub for hand cleaning available	**Available (all areas)**<br>Mean (Sd)<br>Median (LQ, UQ)<br>(Minimum, Maximum)	56.8 (34.3)<br>66.1 (31.7, 88.9)<br>0.0 to 100	61.4 (33.6)<br>68.8 (36.1, 92.7)<br>0.0 to 100	82.7 (18.6)<br>90.7 (68.3, 97.6)<br>34.9 to 100	52.4 (36.5)<br>46.7 (19.5, 97.6)<br>0.0 to 100
	**Limited**<br>Mean (Sd)<br>Median (LQ, UQ)<br>(Minimum, Maximum)	34.4 (26.7)<br>32.2 (11.1, 50.0)<br>0.0 to 100	34.4 (30.4)<br>31.3 (7.3, 61.1)<br>0.0 to 91.7	16.1 (16.8)<br>9.1 (2.4, 30.2)<br>0.0 to 55.8	41.2 (34.9)<br>39.0 (2.4, 77.8)<br>0.0 to 100

		Pre-randomization		Post randomization	
**Characteristic**	**Category/Measure**	**APT-SEPSIS**	**Usual Care**	**APT-SEPSIS**	**Usual Care**
	**None**<br>Mean (Sd)<br>Median (LQ, UQ)<br>(Minimum, Maximum)	8.8 (19.7)<br>0.0 (0.0, 9.8)<br>0.0 to 82.4	4.2 (8.0)<br>0.0 (0.0, 2.9)<br>0.0 to 28.0	1.2 (3.7)<br>0.0 (0.0, 0.0)<br>0.0 to 17.8	6.4 (15.2)<br>0.0 (0.0, 6.7)<br>0.0 to 79.1

Table S14: Pharmacy supply availability – oral antibiotics

Antibiotic (proportion of weeks available)	Category/Measure	Pre-randomization		Post randomization	
		APT-SEPSIS	Usual Care	APT-SEPSIS	Usual Care
Azithromycin	**Available**<br>Mean (Sd)<br>Median (LQ, UQ)<br>(Minimum, Maximum)	42.6 (27.0)<br>41.2 (19.4, 64.7)<br>4.0 to 94.4	47.5 (29.0)<br>46.9 (28.0, 70.6)<br>0.0 to 94.4	45.1 (23.6)<br>43.0 (31.1, 62.2)<br>2.2 to 90.7	52.5 (31.7)<br>53.7 (28.9, 79.1)<br>0.0 to 97.6
	**Limited**<br>Mean (Sd)<br>Median (LQ, UQ)<br>(Minimum, Maximum)	10.9 (9.4)<br>8.8 (4.9, 15.6)<br>0.0 to 31.3	11.3 (13.1)<br>6.3 (2.6, 17.1)<br>0.0 to 52.8	14.3 (15.7)<br>10.2 (2.3, 17.1)<br>0.0 to 55.6	10.9 (13.9)<br>7.0 (0.0, 14.6)<br>0.0 to 60.5
	**None**<br>Mean (Sd)<br>Median (LQ, UQ)<br>(Minimum, Maximum)	46.5 (26.0)<br>45.7 (27.8, 61.0)<br>0.0 to 94.4	41.2 (29.7)<br>35.3 (11.1, 65.8)<br>0.0 to 97.1	40.6 (23.2)<br>38.7 (22.0, 56.1)<br>9.3 to 97.8	36.6 (29.2)<br>30.2 (12.2, 60.0)<br>0.0 to 100
Amoxicillin	**Available**<br>Mean (Sd)<br>Median (LQ, UQ)<br>(Minimum, Maximum)	63.5 (24.4)<br>63.9 (52.8, 82.9)<br>12.5 to 100	69.0 (20.8)<br>68.8 (55.9, 82.4)<br>29.3 to 100	80.3 (18.4)<br>86.7 (67.4, 95.1)<br>34.9 to 100	82.6 (20.2)<br>90.2 (71.1, 97.7)<br>35.6 to 100
	**Limited**<br>Mean (Sd)<br>Median (LQ, UQ)<br>(Minimum, Maximum)	12.9 (17.0)<br>5.4 (2.4, 16.7)<br>0.0 to 68.8	10.0 (12.6)<br>4.9 (0.0, 19.5)<br>0.0 to 38.9	7.7 (12.4)<br>3.4 (0.0, 7.0)<br>0.0 to 58.1	8.7 (12.8)<br>2.3 (0.0, 13.3)<br>0.0 to 44.4

Antibiotic (proportion of weeks available)	Category/Measure	Pre-randomization		Post randomization	
		APT-SEPSIS	Usual Care	APT-SEPSIS	Usual Care
	**None**<br>Mean (Sd)<br>Median (LQ, UQ)<br>(Minimum, Maximum)	23.6 (20.6)<br>16.3 (9.4, 36.1)<br>0.0 to 84.0	21.0 (15.4)<br>24.4 (8.3, 31.3)<br>0.0 to 53.7	12.0 (12.8)<br>6.7 (2.3, 22.0)<br>0.0 to 42.2	8.7 (11.7)<br>2.4 (0.0, 14.6)<br>0.0 to 40.0
Augmentin	**Available**<br>Mean (Sd)<br>Median (LQ, UQ)<br>(Minimum, Maximum)	23.7 (29.6)<br>7.8 (0.0, 37.5)<br>0.0 to 100	26.1 (29.2)<br>14.7 (2.6, 46.9)<br>0.0 to 100	22.5 (28.7)<br>6.8 (0.0, 42.2)<br>0.0 to 93.0	31.5 (32.9)<br>17.1 (0.0, 53.5)<br>0.0 to 90.7
	**Limited**<br>Mean (Sd)<br>Median (LQ, UQ)<br>(Minimum, Maximum)	6.1 (8.8)<br>2.8 (0.0, 9.4)<br>0.0 to 38.2	9.0 (15.2)<br>2.9 (0.0, 13.9)<br>0.0 to 77.8	9.9 (13.7)<br>2.3 (0.0, 19.5)<br>0.0 to 41.9	9.2 (18.7)<br>2.4 (0.0, 9.8)<br>0.0 to 97.6
	**None**<br>Mean (Sd)<br>Median (LQ, UQ)<br>(Minimum, Maximum)	70.2 (30.7)<br>80.6 (50.0, 96.0)<br>0.0 to 100	64.9 (30.4)<br>78.9 (44.4, 88.2)<br>0.0 to 100	67.6 (32.1)<br>74.4 (39.5, 97.8)<br>4.7 to 100	59.3 (36.5)<br>62.2 (20.9, 97.6)<br>2.4 to 100
Cephalosporin	**Available**<br>Mean (Sd)<br>Median (LQ, UQ)<br>(Minimum, Maximum)	21.0 (24.4)<br>10.6 (0.0, 40.6)<br>0.0 to 80.6	19.9 (21.9)<br>11.8 (5.6, 27.8)<br>0.0 to 88.9	21.7 (23.5)<br>12.5 (2.2, 37.2)<br>0.0 to 76.7	19.1 (18.7)<br>14.0 (2.2, 26.8)<br>0.0 to 62.2
	**Limited**<br>Mean (Sd)<br>Median (LQ, UQ)<br>(Minimum, Maximum)	6.7 (10.7)	6.2 (7.6)	6.6 (12.4)	6.2 (9.9)

Antibiotic (proportion of weeks available)	Category/Measure	Pre-randomization		Post randomization	
		APT-SEPSIS	Usual Care	APT-SEPSIS	Usual Care
		2.8 (0.0, 7.3)<br>0.0 to 40.6	3.1 (0.0, 8.3)<br>0.0 to 26.3	1.1 (0.0, 4.9)<br>0.0 to 44.4	2.2 (0.0, 7.0)<br>0.0 to 37.8
	**None**<br>Mean (Sd)<br>Median (LQ, UQ)<br>(Minimum, Maximum)	72.3 (28.2)<br>84.7 (47.1, 96.9)<br>18.8 to 100	73.9 (22.9)<br>81.6 (61.1, 90.2)<br>11.1 to 100	71.7 (30.4)<br>86.4 (39.5, 97.7)<br>15.6 to 100	74.8 (22.1)<br>77.8 (55.6, 97.6)<br>22.2 to 100
Ciprofloxacin	**Available**<br>Mean (Sd)<br>Median (LQ, UQ)<br>(Minimum, Maximum)	60.7 (27.4)<br>68.0 (41.7, 85.4)<br>8.0 to 94.4	65.1 (22.3)<br>63.9 (47.4, 85.3)<br>25.0 to 100	59.7 (24.1)<br>61.9 (46.3, 76.7)<br>4.7 to 100	69.0 (23.8)<br>78.0 (48.9, 86.0)<br>13.3 to 100
	**Limited**<br>Mean (Sd)<br>Median (LQ, UQ)<br>(Minimum, Maximum)	10.7 (11.8)<br>6.3 (3.1, 14.6)<br>0.0 to 56.3	11.3 (14.0)<br>5.6 (0.0, 19.4)<br>0.0 to 50.0	13.9 (18.7)<br>4.4 (2.2, 20.9)<br>0.0 to 79.1	10.7 (16.9)<br>4.7 (0.0, 12.2)<br>0.0 to 64.4
	**None**<br>Mean (Sd)<br>Median (LQ, UQ)<br>(Minimum, Maximum)	28.6 (23.1)<br>20.5 (12.2, 36.1)<br>0.0 to 88.0	23.6 (16.0)<br>19.5 (11.8, 32.0)<br>0.0 to 58.8	26.3 (21.7)<br>22.6 (11.6, 34.1)<br>0.0 to 93.3	20.2 (14.7)<br>18.6 (9.3, 27.9)<br>0.0 to 51.1
Clindamycin	**Available**<br>Mean (Sd)<br>Median (LQ, UQ)<br>(Minimum, Maximum)	4.3 (7.3)<br>0.0 (0.0, 3.1)<br>0.0 to 25.0	2.9 (4.3)<br>0.0 (0.0, 5.3)<br>0.0 to 19.4	3.8 (7.7)<br>0.0 (0.0, 2.4)<br>0.0 to 30.2	2.6 (4.5)<br>0.0 (0.0, 4.7)<br>0.0 to 20.9

**Antibiotic (proportion of weeks available)**	**Category/Measure**	**Pre-randomization**		**Post randomization**	
		**APT-SEPSIS**	**Usual Care**	**APT-SEPSIS**	**Usual Care**
	**Limited**<br>Mean (Sd)<br>Median (LQ, UQ)<br>(Minimum, Maximum)	3.7 (6.9)<br>0.0 (0.0, 4.0)<br>0.0 to 32.4	1.9 (2.9)<br>0.0 (0.0, 2.8)<br>0.0 to 11.8	3.9 (10.0)<br>0.0 (0.0, 2.3)<br>0.0 to 40.0	2.8 (7.9)<br>0.0 (0.0, 2.4)<br>0.0 to 42.2
	**None**<br>Mean (Sd)<br>Median (LQ, UQ)<br>(Minimum, Maximum)	92.0 (12.6)<br>97.2 (92.7, 100)<br>50.0 to 100	95.3 (4.9)<br>96.0 (93.8, 100)<br>80.6 to 100	92.3 (15.0)<br>97.7 (91.1, 100)<br>33.3 to 100	94.6 (9.3)<br>97.7 (95.1, 100)<br>57.8 to 100
Doxycycline	**Available**<br>Mean (Sd)<br>Median (LQ, UQ)<br>(Minimum, Maximum)	69.7 (31.0)<br>86.8 (44.4, 96.0)<br>3.1 to 100	78.0 (21.3)<br>85.3 (61.8, 97.6)<br>32.4 to 100	78.6 (23.4)<br>88.5 (64.4, 97.7)<br>7.0 to 100	80.5 (22.7)<br>90.7 (60.0, 100)<br>17.8 to 100
	**Limited**<br>Mean (Sd)<br>Median (LQ, UQ)<br>(Minimum, Maximum)	11.4 (22.0)<br>2.9 (0.0, 8.3)<br>0.0 to 96.9	9.7 (13.5)<br>3.1 (0.0, 12.2)<br>0.0 to 44.4	9.1 (19.5)<br>1.1 (0.0, 8.9)<br>0.0 to 93.0	9.0 (16.8)<br>2.4 (0.0, 9.8)<br>0.0 to 80.0
	**None**<br>Mean (Sd)<br>Median (LQ, UQ)<br>(Minimum, Maximum)	18.9 (25.6)<br>6.9 (0.0, 29.3)<br>0.0 to 94.1	12.3 (15.5)<br>3.1 (0.0, 22.2)<br>0.0 to 58.8	12.3 (16.2)<br>4.7 (0.0, 17.1)<br>0.0 to 55.6	10.5 (15.2)<br>2.3 (0.0, 17.8)<br>0.0 to 53.3
Erythromycin	**Available**<br>Mean (Sd)<br>Median (LQ, UQ)<br>(Minimum, Maximum)	48.2 (28.8)	58.8 (27.4)	48.2 (25.9)	58.8 (29.0)

Antibiotic (proportion of weeks available)	Category/Measure	Pre-randomization		Post randomization	
		APT-SEPSIS	Usual Care	APT-SEPSIS	Usual Care
		51.8 (25.0, 75.0)<br>0.0 to 92.7	63.2 (39.0, 79.4)<br>8.3 to 100	44.0 (28.9, 68.3)<br>4.7 to 93.3	67.4 (37.8, 86.0)<br>11.1 to 100
	**Limited**<br>Mean (Sd)<br>Median (LQ, UQ)<br>(Minimum, Maximum)	13.1 (17.2)<br>6.1 (2.4, 16.7)<br>0.0 to 78.0	10.9 (11.2)<br>5.9 (2.6, 19.4)<br>0.0 to 31.7	15.7 (16.7)<br>9.3 (2.4, 22.2)<br>0.0 to 55.8	11.8 (15.4)<br>4.7 (0.0, 17.8)<br>0.0 to 62.2
	**None**<br>Mean (Sd)<br>Median (LQ, UQ)<br>(Minimum, Maximum)	38.7 (26.9)<br>31.3 (17.6, 52.9)<br>2.4 to 100	30.3 (26.4)<br>22.2 (10.5, 51.2)<br>0.0 to 91.7	36.1 (22.3)<br>30.7 (18.6, 53.7)<br>0.0 to 73.3	29.4 (24.6)<br>20.9 (11.6, 43.9)<br>0.0 to 80.5
Flucloxacillin	**Available**<br>Mean (Sd)<br>Median (LQ, UQ)<br>(Minimum, Maximum)	23.1 (22.0)<br>20.0 (2.4, 38.2)<br>0.0 to 72.2	25.0 (24.5)<br>22.0 (2.9, 40.6)<br>0.0 to 80.6	22.6 (23.6)<br>18.2 (2.2, 41.9)<br>0.0 to 95.3	34.2 (32.9)<br>31.7 (2.2, 60.5)<br>0.0 to 100
	**Limited**<br>Mean (Sd)<br>Median (LQ, UQ)<br>(Minimum, Maximum)	8.5 (13.4)<br>0.0 (0.0, 12.0)<br>0.0 to 53.1	5.6 (6.9)<br>2.8 (0.0, 8.3)<br>0.0 to 29.4	11.7 (15.4)<br>2.4 (0.0, 20.9)<br>0.0 to 51.2	6.4 (8.6)<br>2.2 (0.0, 8.9)<br>0.0 to 26.8
	**None**<br>Mean (Sd)<br>Median (LQ, UQ)<br>(Minimum, Maximum)	68.4 (25.9)<br>73.5 (43.8, 97.1)<br>27.8 to 100	69.5 (24.6)<br>68.4 (52.9, 92.0)<br>19.4 to 100	65.7 (30.2)<br>69.3 (44.2, 97.6)<br>0.0 to 100	59.4 (33.7)<br>53.5 (34.9, 97.8)<br>0.0 to 100

Antibiotic (proportion of weeks available)	Category/Measure	Pre-randomization		Post randomization	
		APT-SEPSIS	Usual Care	APT-SEPSIS	Usual Care
Metronidazole	**Available**<br>Mean (Sd)<br>Median (LQ, UQ)<br>(Minimum, Maximum)	71.8 (24.8)<br>79.3 (58.8, 93.8)<br>6.3 to 100	74.4 (18.8)<br>78.0 (61.1, 90.6)<br>27.8 to 100	78.3 (22.2)<br>86.4 (64.4, 93.3)<br>14.0 to 100	84.6 (20.6)<br>91.1 (80.0, 100)<br>15.6 to 100
	**Limited**<br>Mean (Sd)<br>Median (LQ, UQ)<br>(Minimum, Maximum)	11.1 (17.1)<br>3.6 (0.0, 11.1)<br>0.0 to 68.8	9.8 (12.7)<br>3.1 (0.0, 13.9)<br>0.0 to 41.2	9.8 (18.6)<br>2.3 (0.0, 7.0)<br>0.0 to 69.8	7.6 (15.2)<br>2.2 (0.0, 8.9)<br>0.0 to 77.8
	**None**<br>Mean (Sd)<br>Median (LQ, UQ)<br>(Minimum, Maximum)	17.1 (16.7)<br>11.7 (5.9, 25.0)<br>0.0 to 76.0	15.9 (12.8)<br>15.6 (5.6, 28.0)<br>0.0 to 44.0	11.9 (13.4)<br>6.8 (2.3, 16.3)<br>0.0 to 55.6	7.8 (10.5)<br>2.3 (0.0, 12.2)<br>0.0 to 37.8

Table S15: Pharmacy supply availability – Intravenous antibiotics

Antibiotic (proportion of weeks available)	Category/Measure	Pre-randomization		Post randomization	
		APT-SEPSIS	Usual Care	APT-SEPSIS	Usual Care
IV Ampicillin	**Available**<br>Mean (Sd)<br>Median (LQ, UQ)<br>(Minimum, Maximum)	47.8 (29.5)<br>50.0 (33.3, 68.0)<br>0.0 to 97.1	47.3 (32.5)<br>44.4 (18.8, 80.0)<br>0.0 to 100	47.4 (31.3)<br>50.0 (23.3, 65.9)<br>0.0 to 100	45.7 (23.5)<br>46.7 (34.9, 61.0)<br>2.3 to 97.8
	**Limited**<br>Mean (Sd)<br>Median (LQ, UQ)<br>(Minimum, Maximum)	7.6 (8.1)<br>6.8 (0.0, 15.6)<br>0.0 to 29.4	7.8 (13.5)<br>2.4 (0.0, 8.0)<br>0.0 to 50.0	7.8 (11.0)<br>2.4 (0.0, 12.2)<br>0.0 to 37.2	9.3 (11.6)<br>4.4 (0.0, 17.1)<br>0.0 to 39.5
	**None**<br>Mean (Sd)<br>Median (LQ, UQ)<br>(Minimum, Maximum)	44.6 (30.5)<br>41.7 (18.8, 58.5)<br>0.0 to 100	44.8 (34.0)<br>41.2 (16.7, 75.0)<br>0.0 to 100	44.8 (32.9)<br>39.3 (14.6, 64.4)<br>0.0 to 100	45.0 (27.5)<br>39.0 (22.2, 60.5)<br>0.0 to 97.6
IV Benzylpenicillin	**Available**<br>Mean (Sd)<br>Median (LQ, UQ)<br>(Minimum, Maximum)	64.4 (21.9)<br>68.1 (50.0, 78.0)<br>12.5 to 100	72.7 (18.0)<br>75.6 (63.2, 85.4)<br>25.0 to 100	77.9 (23.7)<br>87.5 (61.0, 95.3)<br>24.4 to 100	81.2 (20.7)<br>87.8 (73.3, 97.7)<br>35.6 to 100
	**Limited**<br>Mean (Sd)<br>Median (LQ, UQ)<br>(Minimum, Maximum)	10.4 (14.6)<br>6.8 (0.0, 12.2)<br>0.0 to 65.6	9.7 (10.1)<br>8.0 (0.0, 19.4)<br>0.0 to 35.3	7.9 (13.7)<br>2.4 (0.0, 9.3)<br>0.0 to 69.8	6.3 (10.1)<br>2.2 (0.0, 9.3)<br>0.0 to 44.4

Antibiotic (proportion of weeks available)	Category/Measure	Pre-randomization		Post randomization	
		APT-SEPSIS	Usual Care	APT-SEPSIS	Usual Care
	**None**<br>Mean (Sd)<br>Median (LQ, UQ)<br>(Minimum, Maximum)	25.2 (18.6)<br>22.0 (11.8, 36.1)<br>0.0 to 72.2	17.6 (12.9)<br>17.1 (7.9, 23.5)<br>0.0 to 52.8	14.2 (18.9)<br>4.7 (0.0, 24.4)<br>0.0 to 66.7	12.5 (16.7)<br>4.4 (0.0, 20.0)<br>0.0 to 62.2
IV Cefazolin	**Available**<br>Mean (Sd)<br>Median (LQ, UQ)<br>(Minimum, Maximum)	5.9 (11.7)<br>0.0 (0.0, 8.3)<br>0.0 to 50.0	2.5 (6.0)<br>0.0 (0.0, 2.8)<br>0.0 to 32.4	6.5 (11.7)<br>0.0 (0.0, 6.7)<br>0.0 to 46.5	3.9 (13.0)<br>0.0 (0.0, 2.3)<br>0.0 to 69.8
	**Limited**<br>Mean (Sd)<br>Median (LQ, UQ)<br>(Minimum, Maximum)	5.8 (12.9)<br>0.0 (0.0, 4.0)<br>0.0 to 50.0	2.1 (4.5)<br>0.0 (0.0, 2.8)<br>0.0 to 23.5	3.8 (9.4)<br>0.0 (0.0, 2.2)<br>0.0 to 35.6	1.9 (4.0)<br>0.0 (0.0, 2.3)<br>0.0 to 20.0
	**None**<br>Mean (Sd)<br>Median (LQ, UQ)<br>(Minimum, Maximum)	88.3 (21.2)<br>97.6 (88.9, 100)<br>28.1 to 100	95.4 (10.2)<br>97.2 (94.7, 100)<br>44.1 to 100	89.7 (17.3)<br>97.8 (86.7, 100)<br>39.5 to 100	94.1 (14.7)<br>97.8 (95.1, 100)<br>25.6 to 100
IV Cephalosporin (e.g. Ceftriaxone etc)	**Available**<br>Mean (Sd)<br>Median (LQ, UQ)<br>(Minimum, Maximum)	62.7 (30.4)<br>66.6 (33.3, 92.7)<br>4.0 to 100	67.0 (29.3)<br>75.6 (58.8, 87.5)<br>4.0 to 100	68.1 (24.8)<br>74.4 (46.7, 90.7)<br>14.6 to 100	75.3 (27.4)<br>82.9 (70.7, 95.3)<br>6.7 to 100
	**Limited**<br>Mean (Sd)<br>Median (LQ, UQ)<br>(Minimum, Maximum)	10.4 (14.8)	7.1 (8.3)	10.4 (14.1)	7.6 (12.4)

Antibiotic (proportion of weeks available)	Category/Measure	Pre-randomization		Post randomization	
		APT-SEPSIS	Usual Care	APT-SEPSIS	Usual Care
		5.9 (0.0, 16.0)<br>0.0 to 68.8	4.0 (0.0, 9.4)<br>0.0 to 30.6	4.5 (0.0, 15.6)<br>0.0 to 58.1	2.3 (0.0, 6.7)<br>0.0 to 46.7
	**None**<br>Mean (Sd)<br>Median (LQ, UQ)<br>(Minimum, Maximum)	26.9 (25.1)<br>22.9 (3.1, 47.1)<br>0.0 to 96.0	25.9 (25.1)<br>19.5 (3.1, 41.2)<br>0.0 to 96.0	21.5 (20.7)<br>17.4 (4.4, 31.1)<br>0.0 to 78.0	17.0 (20.3)<br>9.3 (2.3, 20.0)<br>0.0 to 73.3
IV Chloramphenicol	**Available**<br>Mean (Sd)<br>Median (LQ, UQ)<br>(Minimum, Maximum)	19.4 (23.7)<br>9.1 (2.9, 32.4)<br>0.0 to 91.2	24.4 (28.9)<br>5.6 (0.0, 52.6)<br>0.0 to 78.9	22.6 (24.8)<br>10.7 (2.4, 35.6)<br>0.0 to 84.4	28.5 (29.9)<br>15.6 (2.3, 57.8)<br>0.0 to 90.2
	**Limited**<br>Mean (Sd)<br>Median (LQ, UQ)<br>(Minimum, Maximum)	4.8 (8.7)<br>0.0 (0.0, 5.6)<br>0.0 to 35.3	6.9 (10.9)<br>2.4 (0.0, 8.3)<br>0.0 to 36.1	4.8 (8.0)<br>2.2 (0.0, 4.7)<br>0.0 to 37.8	6.2 (13.8)<br>2.2 (0.0, 4.7)<br>0.0 to 68.9
	**None**<br>Mean (Sd)<br>Median (LQ, UQ)<br>(Minimum, Maximum)	75.8 (28.6)<br>89.4 (61.1, 96.9)<br>8.0 to 100	68.8 (34.5)<br>91.7 (44.1, 97.6)<br>2.8 to 100	72.6 (28.3)<br>85.2 (53.3, 95.3)<br>6.7 to 100	65.2 (33.0)<br>73.3 (34.1, 97.7)<br>4.9 to 100
IV Ciprofloxacin	**Available**<br>Mean (Sd)<br>Median (LQ, UQ)<br>(Minimum, Maximum)	15.0 (21.0)<br>5.9 (0.0, 19.4)<br>0.0 to 63.9	19.4 (24.4)<br>6.3 (2.8, 24.0)<br>0.0 to 88.2	16.1 (23.8)<br>4.5 (0.0, 24.4)<br>0.0 to 83.7	20.0 (26.6)<br>6.7 (2.3, 27.9)<br>0.0 to 80.5

Antibiotic (proportion of weeks available)	Category/Measure	Pre-randomization		Post randomization	
		APT-SEPSIS	Usual Care	APT-SEPSIS	Usual Care
	**Limited**<br>Mean (Sd)<br>Median (LQ, UQ)<br>(Minimum, Maximum)	3.9 (6.7)<br>0.0 (0.0, 4.9)<br>0.0 to 21.9	4.4 (9.3)<br>0.0 (0.0, 4.0)<br>0.0 to 41.2	3.3 (8.6)<br>0.0 (0.0, 2.2)<br>0.0 to 40.0	6.2 (14.5)<br>0.0 (0.0, 2.3)<br>0.0 to 64.4
	**None**<br>Mean (Sd)<br>Median (LQ, UQ)<br>(Minimum, Maximum)	81.1 (25.4)<br>93.8 (73.5, 97.6)<br>15.6 to 100	76.2 (27.3)<br>92.7 (63.2, 97.1)<br>2.9 to 100	80.6 (28.2)<br>94.4 (73.3, 100)<br>6.7 to 100	73.7 (31.6)<br>93.0 (51.2, 97.6)<br>17.1 to 100
IV Clindamycin	**Available**<br>Mean (Sd)<br>Median (LQ, UQ)<br>(Minimum, Maximum)	2.5 (5.9)<br>0.0 (0.0, 0.0)<br>0.0 to 22.2	1.2 (2.7)<br>0.0 (0.0, 0.0)<br>0.0 to 12.5	4.3 (8.8)<br>0.0 (0.0, 4.4)<br>0.0 to 36.6	3.0 (6.0)<br>0.0 (0.0, 2.3)<br>0.0 to 23.3
	**Limited**<br>Mean (Sd)<br>Median (LQ, UQ)<br>(Minimum, Maximum)	3.9 (9.1)<br>0.0 (0.0, 2.8)<br>0.0 to 44.1	1.0 (2.4)<br>0.0 (0.0, 0.0)<br>0.0 to 8.8	4.6 (11.6)<br>0.0 (0.0, 2.2)<br>0.0 to 42.2	2.5 (8.5)<br>0.0 (0.0, 0.0)<br>0.0 to 44.4
	**None**<br>Mean (Sd)<br>Median (LQ, UQ)<br>(Minimum, Maximum)	93.6 (12.7)<br>100 (94.4, 100)<br>50.0 to 100	97.8 (3.7)<br>100 (97.1, 100)<br>87.5 to 100	91.1 (16.7)<br>100 (95.1, 100)<br>44.4 to 100	94.5 (10.6)<br>100 (97.6, 100)<br>55.6 to 100
IV Co-amoxiclav	**Available**<br>Mean (Sd)<br>Median (LQ, UQ)<br>(Minimum, Maximum)	4.5 (10.0)	3.9 (12.0)	3.2 (7.7)	5.5 (16.0)

Antibiotic (proportion of weeks available)	Category/Measure	Pre-randomization		Post randomization	
		**APT-SEPSIS**	**Usual Care**	**APT-SEPSIS**	**Usual Care**
		0.0 (0.0, 2.8)<br>0.0 to 37.5	0.0 (0.0, 2.6)<br>0.0 to 59.4	0.0 (0.0, 2.2)<br>0.0 to 32.6	2.2 (0.0, 2.4)<br>0.0 to 86.0
	**Limited**<br>Mean (Sd)<br>Median (LQ, UQ)<br>(Minimum, Maximum)	3.8 (8.5)<br>0.0 (0.0, 4.0)<br>0.0 to 44.1	1.8 (5.2)<br>0.0 (0.0, 0.0)<br>0.0 to 26.5	2.9 (8.4)<br>0.0 (0.0, 2.2)<br>0.0 to 35.6	2.0 (3.9)<br>0.0 (0.0, 2.3)<br>0.0 to 15.6
	**None**<br>Mean (Sd)<br>Median (LQ, UQ)<br>(Minimum, Maximum)	91.8 (14.6)<br>97.2 (93.8, 100)<br>46.9 to 100	94.3 (13.0)<br>100 (96.9, 100)<br>40.6 to 100	93.8 (13.0)<br>100 (95.6, 100)<br>51.1 to 100	92.5 (17.9)<br>97.7 (95.1, 100)<br>4.7 to 100
IV Gentamycin	**Available**<br>Mean (Sd)<br>Median (LQ, UQ)<br>(Minimum, Maximum)	65.6 (23.8)<br>72.1 (50.0, 82.9)<br>6.3 to 100	69.1 (23.8)<br>65.9 (55.6, 93.8)<br>26.8 to 100	65.8 (22.7)<br>69.5 (53.3, 82.2)<br>7.0 to 100	71.7 (22.5)<br>74.4 (58.5, 85.4)<br>17.8 to 100
	**Limited**<br>Mean (Sd)<br>Median (LQ, UQ)<br>(Minimum, Maximum)	12.0 (13.4)<br>8.3 (2.4, 17.1)<br>0.0 to 47.1	9.7 (12.1)<br>3.1 (0.0, 14.6)<br>0.0 to 41.2	9.2 (13.3)<br>3.5 (0.0, 11.1)<br>0.0 to 51.2	10.1 (14.8)<br>4.4 (0.0, 16.3)<br>0.0 to 62.2
	**None**<br>Mean (Sd)<br>Median (LQ, UQ)<br>(Minimum, Maximum)	22.4 (17.2)<br>17.6 (8.3, 30.6)<br>0.0 to 68.0	21.2 (18.6)<br>18.8 (3.1, 34.1)<br>0.0 to 68.0	25.1 (17.1)<br>23.9 (9.3, 36.6)<br>0.0 to 62.8	18.3 (15.1)<br>15.6 (4.7, 28.9)<br>0.0 to 56.1

Antibiotic (proportion of weeks available)	Category/Measure	Pre-randomization		Post randomization	
		APT-SEPSIS	Usual Care	APT-SEPSIS	Usual Care
IV Metronidazole	**Available**<br>Mean (Sd)<br>Median (LQ, UQ)<br>(Minimum, Maximum)	57.8 (27.7)<br>60.6 (36.6, 82.4)<br>8.8 to 100	64.0 (22.9)<br>63.2 (44.1, 81.3)<br>32.0 to 100	65.5 (22.7)<br>69.5 (53.3, 88.4)<br>2.3 to 95.6	71.0 (20.7)<br>73.3 (62.2, 87.8)<br>22.2 to 100
	**Limited**<br>Mean (Sd)<br>Median (LQ, UQ)<br>(Minimum, Maximum)	10.8 (12.1)<br>7.5 (2.8, 12.0)<br>0.0 to 46.9	9.1 (10.7)<br>4.0 (0.0, 14.6)<br>0.0 to 38.2	9.5 (12.1)<br>5.9 (2.2, 11.6)<br>0.0 to 46.7	7.5 (11.7)<br>4.4 (0.0, 9.8)<br>0.0 to 55.6
	**None**<br>Mean (Sd)<br>Median (LQ, UQ)<br>(Minimum, Maximum)	31.4 (24.9)<br>23.2 (12.2, 58.3)<br>0.0 to 91.2	26.9 (19.7)<br>26.8 (9.4, 41.7)<br>0.0 to 64.0	25.0 (19.3)<br>19.8 (8.9, 39.5)<br>4.4 to 76.7	21.5 (14.5)<br>24.4 (12.2, 31.1)<br>0.0 to 60.0
IV Penicillin G	**Available**<br>Mean (Sd)<br>Median (LQ, UQ)<br>(Minimum, Maximum)	34.4 (32.6)<br>29.4 (0.0, 67.6)<br>0.0 to 97.2	29.4 (27.8)<br>24.4 (5.6, 43.9)<br>0.0 to 97.2	34.7 (35.1)<br>20.0 (2.4, 61.0)<br>0.0 to 100	37.7 (33.9)<br>27.9 (7.3, 55.8)<br>2.2 to 100
	**Limited**<br>Mean (Sd)<br>Median (LQ, UQ)<br>(Minimum, Maximum)	4.0 (5.6)<br>0.0 (0.0, 8.0)<br>0.0 to 23.5	3.9 (7.1)<br>0.0 (0.0, 4.9)<br>0.0 to 29.4	3.8 (9.7)<br>0.0 (0.0, 2.3)<br>0.0 to 44.4	3.3 (6.8)<br>0.0 (0.0, 2.4)<br>0.0 to 31.1
	**None**<br>Mean (Sd)<br>Median (LQ, UQ)<br>(Minimum, Maximum)	61.6 (33.2)	66.7 (27.9)	61.5 (36.7)	59.0 (34.1)

Antibiotic (proportion of weeks available)	Category/Measure	Pre-randomization		Post randomization	
		APT-SEPSIS	Usual Care	APT-SEPSIS	Usual Care
		62.9 (31.3, 100)<br>2.8 to 100	75.0 (56.1, 88.9)<br>2.8 to 100	75.6 (33.3, 97.6)<br>0.0 to 100	60.0 (44.2, 92.7)<br>0.0 to 97.8
IV Vancomycin	**Available**<br>Mean (Sd)<br>Median (LQ, UQ)<br>(Minimum, Maximum)	3.4 (6.5)<br>0.0 (0.0, 3.1)<br>0.0 to 23.5	2.2 (6.1)<br>0.0 (0.0, 2.4)<br>0.0 to 31.3	3.5 (13.4)<br>0.0 (0.0, 0.0)<br>0.0 to 72.1	0.8 (1.4)<br>0.0 (0.0, 2.2)<br>0.0 to 4.9
	**Limited**<br>Mean (Sd)<br>Median (LQ, UQ)<br>(Minimum, Maximum)	3.8 (9.5)<br>0.0 (0.0, 0.0)<br>0.0 to 40.6	1.1 (4.4)<br>0.0 (0.0, 0.0)<br>0.0 to 23.5	4.4 (11.4)<br>0.0 (0.0, 2.2)<br>0.0 to 44.4	2.5 (7.8)<br>0.0 (0.0, 0.0)<br>0.0 to 33.3
	**None**<br>Mean (Sd)<br>Median (LQ, UQ)<br>(Minimum, Maximum)	92.8 (14.7)<br>100 (96.0, 100)<br>47.1 to 100	96.7 (8.2)<br>100 (97.6, 100)<br>67.6 to 100	92.1 (17.8)<br>100 (95.6, 100)<br>23.3 to 100	96.7 (8.3)<br>100 (97.6, 100)<br>66.7 to 100
IV Meropenem	**Available**<br>Mean (Sd)<br>Median (LQ, UQ)<br>(Minimum, Maximum)	5.8 (10.8)<br>0.0 (0.0, 7.3)<br>0.0 to 38.2	6.6 (13.2)<br>0.0 (0.0, 4.9)<br>0.0 to 44.7	7.4 (17.3)<br>0.0 (0.0, 2.3)<br>0.0 to 71.1	2.0 (4.5)<br>0.0 (0.0, 2.2)<br>0.0 to 16.3
	**Limited**<br>Mean (Sd)<br>Median (LQ, UQ)<br>(Minimum, Maximum)	4.7 (10.2)<br>0.0 (0.0, 5.6)<br>0.0 to 43.8	2.7 (9.4)<br>0.0 (0.0, 0.0)<br>0.0 to 47.4	5.7 (12.5)<br>0.0 (0.0, 2.3)<br>0.0 to 53.3	4.7 (15.3)<br>0.0 (0.0, 0.0)<br>0.0 to 66.7

Antibiotic (proportion of weeks available)	Category/Measure	Pre-randomization		Post randomization	
		APT-SEPSIS	Usual Care	APT-SEPSIS	Usual Care
	**None**<br>Mean (Sd)<br>Median (LQ, UQ)<br>(Minimum, Maximum)	89.5 (19.6)<br>100 (86.1, 100)<br>29.4 to 100	90.7 (20.2)<br>100 (95.1, 100)<br>7.9 to 100	87.0 (25.7)<br>100 (92.7, 100)<br>8.9 to 100	93.3 (17.1)<br>100 (97.6, 100)<br>31.1 to 100
IV Tazobactam (Tazocin) or Piperacillin/Tazobactam	**Available**<br>Mean (Sd)<br>Median (LQ, UQ)<br>(Minimum, Maximum)	4.0 (8.0)<br>0.0 (0.0, 3.1)<br>0.0 to 26.5	4.8 (11.9)<br>0.0 (0.0, 2.6)<br>0.0 to 47.4	3.5 (6.6)<br>0.0 (0.0, 2.4)<br>0.0 to 24.4	3.2 (8.7)<br>0.0 (0.0, 2.4)<br>0.0 to 46.7
	**Limited**<br>Mean (Sd)<br>Median (LQ, UQ)<br>(Minimum, Maximum)	4.0 (8.5)<br>0.0 (0.0, 2.8)<br>0.0 to 32.4	2.6 (8.3)<br>0.0 (0.0, 0.0)<br>0.0 to 39.5	5.0 (14.0)<br>0.0 (0.0, 2.2)<br>0.0 to 68.9	3.8 (13.5)<br>0.0 (0.0, 0.0)<br>0.0 to 62.2
	**None**<br>Mean (Sd)<br>Median (LQ, UQ)<br>(Minimum, Maximum)	91.9 (15.3)<br>100 (96.0, 100)<br>41.2 to 100	92.6 (19.2)<br>100 (97.1, 100)<br>13.2 to 100	91.5 (17.8)<br>100 (92.7, 100)<br>13.3 to 100	92.9 (16.5)<br>100 (95.3, 100)<br>33.3 to 100

Table S16: Availability of other pharmacy supplies

Proportion of weeks available	Category/Measure	Pre-randomization		Post randomization	
		APT-SEPSIS	Usual Care	APT-SEPSIS	Usual Care
Intravenous fluids (0.9% Saline or Hartmanns or Ringers lactate)	**Available**<br>Mean (Sd)<br>Median (LQ, UQ)<br>(Minimum, Maximum)	76.6 (23.4)<br>84.7 (61.0, 95.1)<br>28.0 to 100	73.7 (26.6)<br>81.6 (50.0, 96.9)<br>11.1 to 100	79.6 (19.1)<br>85.7 (68.9, 95.3)<br>22.2 to 100	83.8 (14.8)<br>85.4 (75.6, 97.7)<br>46.7 to 100
	**Limited**<br>Mean (Sd)<br>Median (LQ, UQ)<br>(Minimum, Maximum)	13.9 (15.2)<br>11.8 (3.1, 16.0)<br>0.0 to 68.8	17.1 (19.3)<br>8.8 (2.8, 29.3)<br>0.0 to 80.6	14.1 (17.2)<br>10.4 (2.3, 14.6)<br>0.0 to 64.4	10.8 (10.7)<br>8.9 (0.0, 17.8)<br>0.0 to 35.6
	**None**<br>Mean (Sd)<br>Median (LQ, UQ)<br>(Minimum, Maximum)	9.5 (14.8)<br>2.6 (0.0, 13.9)<br>0.0 to 56.0	9.2 (11.9)<br>2.4 (0.0, 16.0)<br>0.0 to 32.4	6.3 (8.6)<br>2.2 (0.0, 13.3)<br>0.0 to 28.9	5.4 (9.1)<br>2.2 (0.0, 6.7)<br>0.0 to 33.3

Table S17: Labor ward equipment availability

Characteristic (proportion of weeks available)	Category/Measure	Pre-randomization		Post-randomization	
		APT-SEPSIS	Usual Care	APT-SEPSIS	Usual Care
Antiseptic for skin preparation	**Available**<br>Mean (Sd)<br>Median (LQ, UQ)<br>(Minimum, Maximum)	78.5 (23.1)<br>85.8 (72.0, 94.4)<br>11.8 to 100	74.7 (20.9)<br>73.5 (61.1, 93.8)<br>13.9 to 100	89.5 (15.6)<br>95.5 (82.9, 100)<br>41.9 to 100	77.8 (18.8)<br>82.2 (65.1, 95.1)<br>40.0 to 100
	**Limited**<br>Mean (Sd)<br>Median (LQ, UQ)<br>(Minimum, Maximum)	10.6 (15.9)<br>5.6 (0.0, 11.1)<br>0.0 to 75.0	12.1 (11.6)<br>11.8 (0.0, 16.7)<br>0.0 to 42.1	4.4 (8.6)<br>1.1 (0.0, 4.7)<br>0.0 to 44.2	11.0 (14.0)<br>7.0 (0.0, 13.3)<br>0.0 to 55.6
	**None**<br>Mean (Sd)<br>Median (LQ, UQ)<br>(Minimum, Maximum)	10.9 (14.6)<br>4.8 (0.0, 14.7)<br>0.0 to 58.8	13.2 (15.9)<br>8.3 (2.6, 19.4)<br>0.0 to 69.4	6.1 (11.2)<br>2.2 (0.0, 7.3)<br>0.0 to 51.1	11.2 (13.1)<br>7.0 (0.0, 17.8)<br>0.0 to 48.9
Surgical gloves available	**Available**<br>Mean (Sd)<br>Median (LQ, UQ)<br>(Minimum, Maximum)	76.3 (22.7)<br>83.4 (61.1, 96.9)<br>12.5 to 100	64.6 (32.3)<br>72.0 (39.0, 91.7)<br>0.0 to 100	86.2 (16.3)<br>93.0 (82.2, 97.7)<br>40.0 to 100	80.0 (24.4)<br>85.4 (77.8, 97.7)<br>2.2 to 100
	**Limited**<br>Mean (Sd)<br>Median (LQ, UQ)<br>(Minimum, Maximum)	14.2 (16.2)<br>7.5 (2.4, 25.0)<br>0.0 to 62.5	20.5 (23.8)<br>12.0 (2.8, 34.1)<br>0.0 to 83.3	10.1 (14.7)<br>4.9 (2.2, 11.1)<br>0.0 to 53.5	14.3 (22.7)<br>4.7 (0.0, 11.6)<br>0.0 to 91.1

Characteristic (proportion of weeks available)	Category/Measure	Pre-randomization		Post-randomization	
	**None**<br>Mean (Sd)<br>Median (LQ, UQ)<br>(Minimum, Maximum)	9.5 (11.4)<br>4.9 (0.0, 14.7)<br>0.0 to 44.0	14.8 (19.1)<br>13.9 (0.0, 16.7)<br>0.0 to 86.1	3.7 (6.0)<br>2.2 (0.0, 7.0)<br>0.0 to 26.8	5.7 (6.6)<br>4.4 (0.0, 7.3)<br>0.0 to 22.0
Sterile (clean) linen packs	**Available**<br>Mean (Sd)<br>Median (LQ, UQ)<br>(Minimum, Maximum)	42.5 (33.4)<br>41.6 (8.8, 69.4)<br>0.0 to 100	33.2 (32.4)<br>19.4 (5.3, 61.1)<br>0.0 to 93.8	44.6 (32.4)<br>46.6 (12.2, 69.8)<br>0.0 to 100	34.8 (28.0)<br>31.7 (9.3, 58.5)<br>0.0 to 87.8
	**Limited**<br>Mean (Sd)<br>Median (LQ, UQ)<br>(Minimum, Maximum)	12.2 (14.6)<br>8.6 (0.0, 19.4)<br>0.0 to 56.3	11.6 (13.8)<br>5.6 (0.0, 15.8)<br>0.0 to 47.4	11.2 (16.8)<br>4.4 (0.0, 13.3)<br>0.0 to 55.8	13.6 (17.1)<br>7.3 (2.2, 24.4)<br>0.0 to 80.0
	**None**<br>Mean (Sd)<br>Median (LQ, UQ)<br>(Minimum, Maximum)	45.3 (32.1)<br>46.9 (11.8, 77.8)<br>0.0 to 100	55.2 (32.1)<br>56.0 (29.4, 88.2)<br>3.1 to 100	44.2 (29.9)<br>40.0 (20.9, 64.4)<br>0.0 to 100	51.6 (32.8)<br>55.8 (20.9, 75.6)<br>2.3 to 100
Sufficient sterilized delivery sets	**Available**<br>Mean (Sd)<br>Median (LQ, UQ)<br>(Minimum, Maximum)	61.8 (36.4)<br>76.4 (33.3, 93.8)<br>0.0 to 100	60.7 (35.2)<br>63.2 (28.1, 94.4)<br>0.0 to 100	63.4 (38.5)<br>79.8 (17.8, 100)<br>4.4 to 100	59.7 (33.8)<br>62.8 (33.3, 91.1)<br>2.2 to 100
	**Limited**<br>Mean (Sd)<br>Median (LQ, UQ)<br>(Minimum, Maximum)	22.2 (24.3)<br>14.7 (2.8, 40.0)<br>0.0 to 93.8	29.6 (29.5)<br>26.5 (2.8, 52.6)<br>0.0 to 88.9	21.1 (28.6)<br>6.7 (0.0, 26.7)<br>0.0 to 86.0	32.1 (29.5)<br>24.4 (4.9, 46.7)<br>0.0 to 93.3

Characteristic (proportion of weeks available)	Category/Measure	Pre-randomization		Post-randomization	
	**None**<br>Mean (Sd)<br>Median (LQ, UQ)<br>(Minimum, Maximum)	16.0 (26.8)<br>2.9 (0.0, 16.7)<br>0.0 to 100	9.7 (14.7)<br>2.6 (0.0, 13.2)<br>0.0 to 52.0	15.5 (26.4)<br>2.3 (0.0, 17.1)<br>0.0 to 91.1	8.3 (14.5)<br>2.4 (0.0, 11.1)<br>0.0 to 70.7
Sterilized gowns	**Available**<br>Mean (Sd)<br>Median (LQ, UQ)<br>(Minimum, Maximum)	15.5 (22.5)<br>5.4 (0.0, 22.2)<br>0.0 to 96.0	9.1 (13.9)<br>2.6 (0.0, 12.0)<br>0.0 to 47.1	19.5 (26.4)<br>5.7 (0.0, 30.2)<br>0.0 to 100	9.9 (15.4)<br>2.4 (0.0, 13.3)<br>0.0 to 64.4
	**Limited**<br>Mean (Sd)<br>Median (LQ, UQ)<br>(Minimum, Maximum)	11.7 (17.7)<br>4.4 (0.0, 13.9)<br>0.0 to 78.1	8.0 (12.5)<br>2.4 (0.0, 12.0)<br>0.0 to 47.1	5.8 (14.1)<br>2.2 (0.0, 4.9)<br>0.0 to 76.7	11.6 (20.4)<br>0.0 (0.0, 13.3)<br>0.0 to 67.4
	**None**<br>Mean (Sd)<br>Median (LQ, UQ)<br>(Minimum, Maximum)	72.8 (29.8)<br>86.8 (56.3, 95.1)<br>0.0 to 100	82.9 (23.5)<br>96.0 (65.6, 100)<br>20.6 to 100	74.7 (29.5)<br>89.7 (53.3, 95.6)<br>0.0 to 100	78.5 (27.3)<br>95.1 (58.1, 100)<br>17.8 to 100
Sterilized forceps set	**Available**<br>Mean (Sd)<br>Median (LQ, UQ)<br>(Minimum, Maximum)	57.1 (33.1)<br>57.1 (30.6, 92.0)<br>0.0 to 100	58.8 (29.3)<br>58.8 (43.8, 80.0)<br>0.0 to 100	61.0 (37.0)<br>75.0 (24.4, 97.8)<br>2.2 to 100	57.5 (28.8)<br>55.8 (41.5, 80.0)<br>0.0 to 100
	**Limited**<br>Mean (Sd)<br>Median (LQ, UQ)<br>(Minimum, Maximum)	21.3 (21.8)<br>20.6 (2.4, 33.3)<br>0.0 to 90.6	17.3 (19.8)<br>7.3 (0.0, 27.8)<br>0.0 to 63.9	19.0 (26.3)<br>4.5 (0.0, 31.1)<br>0.0 to 86.0	20.3 (23.6)<br>13.3 (0.0, 31.7)<br>0.0 to 97.8

Characteristic (proportion of weeks available)	Category/Measure	Pre-randomization		Post-randomization	
	**None**<br>Mean (Sd)<br>Median (LQ, UQ)<br>(Minimum, Maximum)	21.6 (22.8)<br>16.3 (2.4, 34.4)<br>0.0 to 79.4	23.9 (28.8)<br>12.2 (0.0, 41.7)<br>0.0 to 100	20.0 (28.4)<br>3.5 (0.0, 44.2)<br>0.0 to 97.8	22.1 (26.1)<br>8.9 (2.3, 37.2)<br>0.0 to 86.0
Working vacuum extractor	**Available**<br>Mean (Sd)<br>Median (LQ, UQ)<br>(Minimum, Maximum)	56.0 (43.1)<br>76.2 (5.9, 97.2)<br>0.0 to 100	59.2 (37.3)<br>64.7 (28.9, 96.9)<br>0.0 to 100	59.2 (40.6)<br>69.4 (15.6, 100)<br>0.0 to 100	66.8 (40.6)<br>88.4 (22.2, 100)<br>0.0 to 100
	**Limited**<br>Mean (Sd)<br>Median (LQ, UQ)<br>(Minimum, Maximum)	14.6 (22.7)<br>4.3 (0.0, 17.6)<br>0.0 to 93.8	10.9 (19.3)<br>2.8 (0.0, 12.2)<br>0.0 to 85.3	14.6 (27.3)<br>0.0 (0.0, 20.0)<br>0.0 to 95.3	8.6 (18.1)<br>0.0 (0.0, 7.0)<br>0.0 to 68.9
	**None**<br>Mean (Sd)<br>Median (LQ, UQ)<br>(Minimum, Maximum)	29.4 (34.2)<br>9.2 (0.0, 52.8)<br>0.0 to 100	29.9 (36.5)<br>8.8 (0.0, 44.0)<br>0.0 to 100	26.2 (32.9)<br>7.1 (0.0, 46.7)<br>0.0 to 97.8	24.6 (36.3)<br>4.7 (0.0, 31.1)<br>0.0 to 100
Suction apparatus with suction tube	**Available**<br>Mean (Sd)<br>Median (LQ, UQ)<br>(Minimum, Maximum)	66.0 (38.0)<br>85.7 (36.0, 97.2)<br>0.0 to 100	68.2 (34.7)<br>86.1 (52.8, 97.2)<br>0.0 to 100	67.2 (38.5)<br>91.1 (28.9, 100)<br>2.2 to 100	71.2 (36.7)<br>95.1 (55.8, 97.7)<br>0.0 to 100
	**Limited**<br>Mean (Sd)<br>Median (LQ, UQ)<br>(Minimum, Maximum)	11.2 (15.0)<br>3.0 (0.0, 16.0)<br>0.0 to 61.8	10.6 (13.4)<br>4.9 (0.0, 15.8)<br>0.0 to 42.1	14.0 (23.1)<br>2.4 (0.0, 22.0)<br>0.0 to 76.7	12.5 (22.8)<br>0.0 (0.0, 15.6)<br>0.0 to 88.9

Characteristic (proportion of weeks available)	Category/Measure	Pre-randomization		Post-randomization	
	**None**<br>Mean (Sd)<br>Median (LQ, UQ)<br>(Minimum, Maximum)	22.8 (32.6)<br>3.5 (0.0, 43.9)<br>0.0 to 100	21.2 (32.1)<br>3.1 (0.0, 26.3)<br>0.0 to 100	18.8 (31.3)<br>2.3 (0.0, 19.5)<br>0.0 to 97.8	16.3 (28.4)<br>2.3 (0.0, 11.1)<br>0.0 to 100
Laceration repair pack	**Available**<br>Mean (Sd)<br>Median (LQ, UQ)<br>(Minimum, Maximum)	55.9 (34.2)<br>61.5 (27.8, 87.8)<br>0.0 to 100	50.9 (36.2)<br>52.8 (19.4, 88.0)<br>0.0 to 100	57.1 (41.5)<br>76.3 (9.3, 97.7)<br>0.0 to 100	49.3 (35.5)<br>46.3 (18.6, 86.7)<br>0.0 to 100
	**Limited**<br>Mean (Sd)<br>Median (LQ, UQ)<br>(Minimum, Maximum)	16.0 (18.6)<br>11.1 (0.0, 20.6)<br>0.0 to 56.3	19.6 (24.7)<br>12.2 (0.0, 29.4)<br>0.0 to 78.9	17.0 (26.7)<br>2.3 (0.0, 22.2)<br>0.0 to 86.0	21.7 (26.7)<br>4.9 (0.0, 39.5)<br>0.0 to 95.6
	**None**<br>Mean (Sd)<br>Median (LQ, UQ)<br>(Minimum, Maximum)	28.2 (30.1)<br>16.1 (2.9, 50.0)<br>0.0 to 97.1	29.5 (31.4)<br>19.5 (4.9, 47.1)<br>0.0 to 100	26.0 (31.0)<br>9.3 (0.0, 51.1)<br>0.0 to 95.6	29.0 (35.2)<br>7.3 (2.2, 58.1)<br>0.0 to 100
Emergency drugs (within expiration limits)	**Available**<br>Mean (Sd)<br>Median (LQ, UQ)<br>(Minimum, Maximum)	71.5 (27.0)<br>76.5 (61.8, 94.4)<br>0.0 to 100	61.6 (32.6)<br>56.1 (30.6, 95.1)<br>0.0 to 100	74.9 (30.4)<br>92.2 (55.6, 97.7)<br>0.0 to 100	62.6 (31.9)<br>63.4 (41.9, 93.0)<br>0.0 to 100
	**Limited**<br>Mean (Sd)<br>Median (LQ, UQ)<br>(Minimum, Maximum)	21.0 (22.4)<br>19.1 (2.4, 29.4)<br>0.0 to 84.4	30.1 (32.1)<br>11.1 (2.4, 62.5)<br>0.0 to 100	20.3 (27.9)<br>4.5 (0.0, 34.1)<br>0.0 to 100	30.2 (31.5)<br>31.7 (0.0, 55.6)<br>0.0 to 97.8

Characteristic (proportion of weeks available)	Category/Measure	Pre-randomization		Post-randomization	
	**None**<br>Mean (Sd)<br>Median (LQ, UQ)<br>(Minimum, Maximum)	7.5 (13.9)<br>1.4 (0.0, 8.8)<br>0.0 to 55.9	8.3 (13.1)<br>2.8 (0.0, 9.8)<br>0.0 to 46.9	4.8 (13.4)<br>0.0 (0.0, 2.2)<br>0.0 to 55.6	7.1 (13.4)<br>2.2 (0.0, 7.3)<br>0.0 to 51.2
Mucus extractor for neonates	**Available**<br>Mean (Sd)<br>Median (LQ, UQ)<br>(Minimum, Maximum)	86.1 (23.3)<br>94.8 (87.5, 100)<br>0.0 to 100	83.9 (22.0)<br>94.1 (75.0, 97.4)<br>12.0 to 100	86.0 (22.1)<br>97.7 (77.8, 100)<br>23.3 to 100	85.6 (24.2)<br>95.6 (85.4, 100)<br>4.4 to 100
	**Limited**<br>Mean (Sd)<br>Median (LQ, UQ)<br>(Minimum, Maximum)	11.0 (20.1)<br>2.8 (0.0, 11.8)<br>0.0 to 96.9	10.3 (17.1)<br>2.4 (0.0, 12.0)<br>0.0 to 55.3	12.5 (21.2)<br>0.0 (0.0, 15.6)<br>0.0 to 74.4	8.8 (18.3)<br>0.0 (0.0, 8.9)<br>0.0 to 75.6
	**None**<br>Mean (Sd)<br>Median (LQ, UQ)<br>(Minimum, Maximum)	2.9 (7.9)<br>0.0 (0.0, 2.4)<br>0.0 to 33.3	5.9 (15.5)<br>2.6 (0.0, 4.0)<br>0.0 to 84.0	1.5 (5.3)<br>0.0 (0.0, 0.0)<br>0.0 to 28.9	5.5 (13.7)<br>0.0 (0.0, 4.9)<br>0.0 to 70.7
Bag Valve mask	**Available**<br>Mean (Sd)<br>Median (LQ, UQ)<br>(Minimum, Maximum)	89.8 (20.6)<br>100 (88.9, 100)<br>0.0 to 100	90.0 (17.5)<br>100 (90.6, 100)<br>41.7 to 100	91.0 (18.4)<br>100 (88.9, 100)<br>25.6 to 100	90.9 (21.5)<br>100 (95.3, 100)<br>4.4 to 100
	**Limited**<br>Mean (Sd)<br>Median (LQ, UQ)<br>(Minimum, Maximum)	8.2 (19.7)<br>0.0 (0.0, 8.3)<br>0.0 to 100	8.8 (17.5)<br>0.0 (0.0, 3.1)<br>0.0 to 58.3	7.6 (17.9)<br>0.0 (0.0, 6.7)<br>0.0 to 74.4	8.2 (21.3)<br>0.0 (0.0, 2.4)<br>0.0 to 95.6

Characteristic (proportion of weeks available)	Category/Measure	Pre-randomization		Post-randomization	
	**None**<br>Mean (Sd)<br>Median (LQ, UQ)<br>(Minimum, Maximum)	2.0 (5.8)<br>0.0 (0.0, 0.0)<br>0.0 to 23.5	1.2 (3.2)<br>0.0 (0.0, 0.0)<br>0.0 to 16.0	1.4 (3.9)<br>0.0 (0.0, 0.0)<br>0.0 to 17.8	0.9 (1.7)<br>0.0 (0.0, 2.2)<br>0.0 to 7.0

References

1) World Health Organization. A Guide to the Implementation of the WHO multimodal hand hygiene improvement strategy. Geneva, Switzerland. February 9, 2009. Accessed September 19, 2025. https://www.who.int/publications/i/item/a-guide-to-the-implementation-of-the-who-multimodal-hand-hygiene-improvement-strategy.
2) World Health Organization. WHO guidelines on hand hygiene in health care. Geneva, Switzerland. January 15, 2009. Accessed September 19, 2025. https://www.who.int/publications/i/item/9789241597906.
3) Dunlop CL, Kilpatrick C, Jones L, et al. Adapting the WHO hand hygiene 'reminders in the workplace' to improve acceptability for healthcare workers in maternity settings worldwide: a mixed methods study. BMJ Open 2024 Sep 17;14(9):e083132. 10.1136/bmjopen-2023-083132.
4) Dunlop C. Improving the prevention of maternal infection in global settings. Doctoral dissertation, University of Birmingham. 2022. Accessed September 19, 2025. https://etheses.bham.ac.uk/id/eprint/12367/.
5) World Health Organization. WHO recommendations for prevention and treatment of maternal peripartum infections. Geneva, Switzerland. September 28, 2015. Accessed September 19, 2025. https://www.who.int/publications/i/item/9789241549363.
6) World Health Organization. WHO recommendation on routine antibiotic prophylaxis for women undergoing operative vaginal birth. Geneva, Switzerland. June 18, 2021. Accessed September 19, 2025. https://www.who.int/publications/i/item/9789240027992
7) World Health Organization. WHO recommendations on choice of antiseptic agent and method of application for preoperative skin preparation for caesarean section. Geneva, Switzerland. June 18, 2021. Accessed September 19, 2025. https://www.who.int/publications/i/item/9789240028036
8) World Health Organization. WHO recommendation on vaginal preparation with antiseptic agents for woman undergoing caesarean section. Geneva, Switzerland. June 18, 2021. Accessed September 19, 2025. https://www.who.int/publications/i/item/9789240028067
9) World Health Organization. WHO recommendation on prophylactic antibiotics for woman undergoing caesarean section. Geneva, Switzerland. June 18, 2021. Accessed September 19, 2025. https://www.who.int/publications/i/item/9789240028012
10) Lissauer D, Cheshire J, Dunlop C, et al. Development of the FAST-M maternal sepsis bundle for use in low-resource settings: a modified Delphi process. BJOG 2020 Feb;127(3):416-423. 10.1111/1471-0528.16005.
11) Cheshire J, Jones L, Munthali L, et al. The FAST-M complex intervention for the detection and management of maternal sepsis in low-resource settings: a multi-site evaluation. BJOG 2021 Jul;128(8):1324-1333. 10.1111/1471-0528.16658.
12) Ahmed SI, Rind GK, Sikandar R, et al. Early recognition and management of maternal sepsis in Pakistan: a feasibility study of the implementation of FAST-M intervention. BMJ Open 2023 Jul 30;13(7):e069135. 10.1136/bmjopen-2022-069135.
13) Ahmed SI, Khowaja BMH, Barolia R, et al. Adapting the FAST-M maternal sepsis intervention for implementation in Pakistan: a qualitative exploratory study. BMJ Open 2022 Sep 9;12(9):e059273. 10.1136/bmjopen-2021-059273.

14) Ahmed SI, Khowaja BMH, Barolia R, et al. Evaluation of the FAST-M maternal sepsis intervention in Pakistan: A qualitative exploratory study. PLoS One 2023 Apr 24;18(4):e0284530. 10.1371/journal.pone.0284530.
15) Michie S, van Stralen M, West R. The behaviour change wheel: a new method for characterising and designing behaviour change interventions. Implement Sci 2011 Apr 23:6:42. 10.1186/1748-5908-6-42.
